# The risk of tooth loss in patients with diabetes: A systematic review and meta‐analysis

**DOI:** 10.1111/idh.12512

**Published:** 2021-08-24

**Authors:** Lotte P. M. Weijdijk, Laura Ziukaite, G. A. (Fridus) Van der Weijden, Eric W. P. Bakker, Dagmar Else Slot

**Affiliations:** ^1^ Department of Oral and Maxillofacial Surgery Amsterdam UMC and Academic Center for Dentistry Amsterdam (ACTA) ACTA is a joint venture between the Faculty of Dentistry of the University of Amsterdam and the Faculty of Dentistry of the Vrije Universiteit Amsterdam Amsterdam The Netherlands; ^2^ Clinic for Dentistry Dental Care Utrecht (DCU) Utrecht The Netherlands; ^3^ Department of Periodontology Academic Center for Dentistry Amsterdam (ACTA) ACTA is a joint venture between the Faculty of Dentistry of the University of Amsterdam and the Faculty of Dentistry of the Vrije Universiteit Amsterdam Amsterdam The Netherlands; ^4^ Division Clinical Methods and Public Health Amsterdam UMC University of Amsterdam Amsterdam The Netherlands

**Keywords:** diabetes mellitus, number of teeth, oral health, risk ratio, systematic review, tooth loss

## Abstract

**Aim:**

The aim of this systematic review was to comprehensively and critically summarize and synthesize the risk of losing teeth among with diabetes mellitus (DM) compared to those without DM, as established in observational studies.

**Materials and methods:**

MEDLINE‐PubMed and Cochrane databases were searched through a period from their inception through October 2020 to identify eligible studies. Papers that primarily evaluate the number of teeth in DM patients compared to non‐DM individuals were included. A descriptive analysis of the selected studies was conducted, and when feasible, a meta‐analysis was performed. The quality of the studies was assessed.

**Results:**

A total of 1087 references were generated, and screening of the papers resulted in 10 eligible publications. A descriptive analysis demonstrated that six of these studies indicate a significantly higher risk of tooth loss in DM patients. This was confirmed by the meta‐analysis risk ratio of 1.63 95% CI (1.33; 2.00, *p* < 0.00001). Subgroup analysis illustrates that this is irrespective of the risk‐of‐bias assessment. The higher risk of tooth loss in DM patients was also higher when only DM type II patients or studies with a cross‐sectional design were considered. Patients with a poor DM control status presented a significantly increased risk of tooth loss. When the data were separated by the world continent where the study was performed, Asia and South America had numerically higher risks and a 95% CI that did not overlap with Europe and North America.

**Conclusion:**

There is moderate certainty for a small but significantly higher risk of tooth loss in DM patients as compared to those without DM.

## 1 INTRODUCTION

Tooth loss considerably affects oral health–related quality of life (OHRQoL), causing chewing difficulty, poor dietary intake and functional disorders.^1^ A predominant reason for tooth loss is periodontitis, which is an inflammation of periodontal tissues. Damage from periodontal disease can lead to loosening of teeth and, in a final stage, to tooth loss.^2^, ^3^ The manifestation and progression are influenced by a wide variety of determinants and factors that have been linked with general health. Notably, the association between periodontitis and diabetes mellitus (DM) has been highlighted in the literature. Periodontal disease is considered the sixth complication of DM.^4^ Another primary cause of tooth loss is dental caries. Its development of which is presumably enhanced in DM patients.^5^, ^6^

Due to the ageing population, DM is a growing public health problem, and it likely contributes to a greater demand for health care.^7^ The negative effects of elevated blood sugars on the immune system result in an increased susceptibility to infections.^8^ The risk for development and progression of periodontitis is increased approximately threefold in DM patients as compared to non‐diabetic individuals (non‐DM).^9^, ^10^ Furthermore, DM is associated with increased severity of periodontal disease.^11^ The increased risk of dental caries in DM patients can likely be explained by decreased salivary flow rates^12^ and expanded levels of glucose in the saliva.^13^ The American Diabetes Association and International Diabetes Federation have published DM care guidelines,^7^, ^14^ of which the main goal is prevention and treatment of DM complications, thereby optimizing quality of life (QoL).^14^

Periodontal pocket depth and clinical attachment loss are commonly utilized to define a patient with periodontitis.^15^ However, these outcome measurements are surrogate endpoints of disease. A true endpoint (e.g., tooth loss) would directly assess patients’ experience on the onset of periodontitis.

Moreover, tooth loss also affects QoL.^1^ A recent systematic review (SR) and meta‐analysis assesses predictors of tooth loss, including DM, in periodontitis patients.^16^ However, no SR with a specific focus on the risk of tooth loss in DM patients has yet been performed. In the light of the increasingly available evidence, the aim of this SR is to comprehensively and critically summarize and synthesize the available scientific evidence emerging from observational studies on the number of teeth among DM patients as compared to non‐DM patients.

## 2 METHODS AND MATERIALS

The preparation and presentation of this SR is in accordance with the *Cochrane Handbook for Systematic Reviews* ^17^ and the guideline for Meta‐Analysis of Observational Studies in Epidemiology (MOOSE).^18^

A protocol was developed a priori following the initial discussion between the members of the research team. This study is registered at the ACTA University Ethical Committee by number 2021‐71228.

### 2.1 Focused question

A precise review question was formulated utilizing the population, exposure, comparison, outcomes and study (PECOS) framework as follows^19^:

‐ Is there a higher risk, loosing teeth among patients with DM compared to those without DM, as it was established in observational studies?
‐ Due do a potential link between DM and both caries and periodontitis, it is hypothesized that DM patients are at higher risk, loosing teeth.

### 2.2 Search strategy

A structured search strategy was designed to retrieve all relevant studies that evaluate the number of missing teeth among patients with DM as compared to non‐DM individuals. After consultation with a clinical librarian, the search was designed by two reviewers (L.P.M.W. and D.E.S.). The National Library of Medicine in Washington, DC (MEDLINE‐PubMed), and Cochrane Central were searched from the inception of this study through October 2020 for appropriate papers that answer the focused question. Table 1 provides details regarding the search approach employed. For the search, no limitation was applied on language or date of publication.

**TABLE 1:** Search terms used for PubMed‐MEDLINE. The search strategy was customized according to the database being searched. The following strategy was used in the search: **{[<exposure>] AND [<outcome>]}**

{[ <exposure >] AND [ <outcome >] }
{ [ <exposure> (“diabetes mellitus” [Mesh] OR diabetes OR (diabetes mellitus)[textwords])]
AND
[<outcome> (tooth loss) OR (toothloss) OR (teeth loss) OR (teethloss) OR (teethless) OR (toothless) OR (missing teeth) OR (missing tooth) OR (loss of teeth) OR (loss of tooth) OR (number of teeth) OR number of tooth)))) OR tooth loss [MeSH Terms]) OR number of teeth [MeSH Terms])]}

The reference lists of the studies included in this review were hand‐searched to identify additional potentially relevant studies. Moreover, national (http://www.trialregister.nl) and international trial registries (http://apps.who.int/trialsearch, http://www.ClinicalTrials.gov) were searched for relevant unpublished or ongoing studies. Furthermore, the following database sources were searched for possible relevant studies that have not reached full publications: OpenGrey (http://www.opengrey.eu/), British Library Inside (http://www.bl.uk/inside), the European Federation of Periodontology (http://www.epf.net), the International Association for Dental Research (http://www.iadr.org), Web of Science, BIOSIS Previews and OVID (http://www.ovid.com).

The conference proceedings of the International Association for Dental Research and the European Organization for Caries Research were searched through October 2020. Additionally, the previous 12 months of the following journals were hand‐searched to eliminate potential delay in indexing journals at the National Library of Medicine: *Journal of Operative Dentistry, Journal of Clinical Dentistry, Journal of Dental Research, Journal of Caries Research, International Journal of Dental Hygiene, The Journal of Dental Hygiene, Journal of Clinical Periodontology, The Journal of Periodontology, Periodontology 2000,* *Oral Health and Preventive Dentistry*.

### 2.3 Screening and selection

A two‐stage, electronic data search and selection was performed. First, titles and abstracts (when available) of all studies identified through the searches were screened. Second, details of the selected studies that potentially met the inclusion criteria were further assessed. This process was independently performed by two reviewers (L.P.M.W. and D.E.S.). If the information relevant to the screening criteria was not available in the title or abstract, or if the full text was not retrievable, then the paper was excluded.

Predetermined inclusion criteria for the first screening of titles and abstract were as follows:

● Mentioned in the aim or title of the study:
  ○ The number of teeth present, tooth loss, missing teeth, extracted teeth, decayed‐missed‐filled teeth (DMFT number).
  ○ Diabetes mellitus or any other synonym, such as impaired glucose tolerance, glucose metabolism, glycaemic control or metabolic syndrome, as a single disease (no comorbidities by other systemic diseases).
● Participants were ≥18 years old.

After this phase, full‐text versions were obtained. For the studies that appeared to meet the first set of screening criteria or for which the title and abstract provided insufficient information to make a clear decision, full‐text papers were retrieved. These were read independently by the two review authors, L.P.M.W. and D.E.S.

A full‐text review of all the pertinent articles was completed utilizing the following eligibility criteria:

● Full‐text paper available in English.
● Observational studies: cohort, case‐controlled or cross‐sectional studies. Data should be presented as a cross‐sectional design.
● Studies conducted with human subjects who were:
  ○ ≥18 years.
  ○ In satisfactory general health (no systemic disorders or comorbidities).
  ○ Evaluating a group of patients with DM as well as a group of people without DM.
● DM status:
  ○ Either self‐reported or clinically assessed.
  ○ Type of DM: undefined, type I and/or type II. Prediabetes and gestational diabetes were excluded.
● Reported outcomes:
  ○ Based on a full‐mouth assessment.
  ○ Clinically determined number of teeth (no radiographs).
  ○ Number of missing teeth or number of teeth present as an absolute number of teeth or as a population mean.
  ○ Tooth loss presented as cross‐sectional data for an individual over the lifetime until the moment of assessment (not for the duration of a specific period).

Any disagreement between the two reviewers about the eligibility of studies was resolved after additional discussion. If disagreement persisted, a third reviewer, G.A.W., was consulted, whose judgement was considered to be decisive. Thereafter, the selected full‐text papers that fulfilled all eligibility criteria were identified and included in this SR for data extraction and estimation of the risk of bias. At this stage, the reasons for exclusion were recorded (see online Appendix S1).

### 2.4 Methodological quality assessment

Two reviewers (L.P.M.W. and D.E.S.) independently scored the individual methodological qualities of the included studies utilizing the risk of bias in observational studies of exposures (ROBINS‐E) instrument. This tool assesses risk of bias in non‐randomized studies of exposures and is under development by researchers from University of Bristol (UK), McMaster University (Canada) and the Environmental Protection Agency (USA). The preliminary draft tool version July 2017 was utilized; this instrument is modelled on the risk of bias in non‐randomized studies of interventions (ROBINS‐I) instrument.^20^, ^21^, ^22^

The application of the ROBINS‐E tool consists of the following steps:

‐ Step I: framing the review question, describing potential confounders, co‐interventions and exposure and outcome measurement accuracy information.
‐ Step II: describing each eligible study, including specific confounders and co‐interventions for each study.
‐ Step III: determining risk‐of‐bias consideration through seven items regarding the strengths and limitations of studies.

Quality was assigned as low risk of bias, moderate risk of bias, serious risk of bias, critical risk of bias or no information with the following domains: bias due to confounding, bias in selection, bias in classification, bias due to departures from intended exposures, bias due to missing data, bias in measurement of outcomes and bias in selection of reported results.

The judgements within each domain are carried forward to an overall risk of bias. A study was classified as having a low risk of bias when all domains were judged to be at low risk of bias. Moderate risk of bias was assigned when, for one or more domains, the study was judged not to be higher than moderate risk of bias. A study was classified as having serious risk of bias when, for one or more domains at the most, serious risk of bias was scored. An overall critical risk of bias was scored when at least one domain was judged to be at critical risk of bias. No information was assigned if the study was judged to be at serious or critical risk of bias and there was a lack of information in one or more key domains.^20^, ^21^, ^22^

### 2.5 Data extraction

For those papers that provided insufficient data to be included in the analysis, the first or corresponding authors were contacted by email to query whether additional data could be provided.

Independent data extraction was performed by two reviewers (L.P.M.W. and D.E.S.) utilizing a custom‐designed standardized data extraction form. Disagreement between the reviewers was resolved through discussion and consensus. If disagreement persisted, a third reviewer (G.A.W.) was consulted; this judgement was decisive. Data extraction of all included studies having either an observational, cohort or case‐controlled design was approached as cross‐sectional studies. From the eligible papers, details on study design, demographics, details of the DM status and number of missing teeth or teeth present were extracted. The latter was determined by utilizing the following parameters:

● Total number of evaluated teeth, reference point, either 28 (excluding evaluation of wisdom teeth) or 32 (including wisdom teeth) per included study.
● Number of missing teeth, as an absolute number of teeth or as a population mean of tooth loss.
● Number of teeth present, as an absolute number of teeth or as a population mean. If only the number of currently present teeth is provided, then the number of missing teeth was calculated based on the number of evaluated teeth being either 28 or 32 for each participant.
● The DMFT number; data concerning the number of missing teeth were extracted from this parameter.

When an included study provided multiple age groups of individuals 18 years and older, data were merged so that these were considered as one group. If a DM group was specified in the categories of prediabetes and DM, then the prediabetic data were excluded. When DM types I and II are presented separately in the original included papers, these groups were merged for the overall analysis. If possible, a subgroup analysis on DM types I and II was performed if the original group data allowed for separation of these two groups.

### 2.6 Data analysis

#### 2.6.1 Assessment of clinical and methodological heterogeneity

The factors utilized to assess the clinical heterogeneity of the outcomes of the various studies are as follows:

‐ Characteristics of participants: age, gender and continent.
‐ Evaluable number of teeth.
‐ DM type: I or II.
‐ Method of assessment: professionally diagnosed or self‐reported DM.^23^

Factors employed to assess the methodological heterogeneity were study design details and the total number of evaluated teeth, reference point (28 or 32).

When clinical or methodological heterogeneity was presented across studies, sources of heterogeneity were investigated with subgroup or sensitivity analyses.^17^

As the total number of evaluable teeth (28 or 32) has a direct influence on the relative ratio of the missing teeth to the total number of teeth, this was defined a priori as a reason for subgroup analysis. Other potentially relevant subgroup analyses were study design (studies originally designed as cross‐sectional evaluations), participant demographics, potential risk of bias and the world continent where the study was performed and data were obtained. For DM‐related details, a sub‐analysis was also conducted with respect to DM control (poor or well regulated), insulin dependence (yes or no) and DM duration.

#### 2.6.2 Descriptive methods

As a summary, a descriptive data presentation is utilized for all studies.

#### 2.6.3 Quantitative methods

A meta‐analysis was performed comparing the number of missing teeth among patients with DM to those without DM. For a subsequent subgroup analysis, a meta‐analysis was performed if more than one study could be included. Analysis was performed utilizing Review Manager version 5.3^24^ according to the Preferred Reporting Items for Systematic Reviews and Meta‐Analysis (PRISMA) and MOOSE guidelines^18^, ^25^ as well as the Cochrane handbook.^17^ From the data, the relative risk or risk ratio (RR) with its associated 95% confidence interval and p‐value were calculated for the number of missing teeth among DM patients as compared to non‐DM individuals. *p*‐values ≤ 0.05 were considered to be significant.

The absolute number of teeth per group in a study was utilized so that the data were weighed according to the study population. If the absolute numbers were not provided, then the number of teeth for the entire group was calculated based on the population mean multiplied by the number of participants in each group (DM or non‐DM).

The RR between DM patients and non‐DM individuals was calculated utilizing both random‐ and fixed‐effects models where appropriate. When there was heterogeneity that could not readily be explained, the analytical approach was conducted according to a random‐effects model. If there were less than four studies, then a fixed‐effects analysis was performed because it may be impossible to estimate the between‐study variance with any precision. In such a case, the fixed‐effects model is the only option.^17^

It was expected that there would be considerable heterogeneity among the included studies, as study designs and details presumably differ. Moreover, DM is not likely to be the single cause for tooth loss. Clinically, DM can vary in its features, which is likely and was the case in the DM population of the included studies. This variance was considered by primarily utilizing the random‐effects model, the exception being when less than four studies were eligible for meta‐analysis. Otherwise, the fixed‐effects model was utilized, as advised by the Cochrane Oral health group.^26^

Sensitivity analyses were undertaken to evaluate the effect of excluding studies based on specific aspects in the domain of clinical or methodological heterogeneity. The testing for publication bias per outcome was utilized as proposed by Egger et al.^27^ If the meta‐analysis involved a sufficient number of trials to make visual inspection of the funnel plot meaningful (a minimum of 10 trials), then these plots were employed as tools to assess publication bias. The presence of asymmetry in the inverted funnel is suggestive of publication bias.^17^, ^25^

#### 2.6.4 Assessment of statistical heterogeneity

Statistically, heterogeneity was tested by the chi‐square test and *I* ^2^ statistic. A chi‐square test resulting in a *p* < 0.1 was considered an indication of significant statistical heterogeneity. As a rough guide to assess the possible magnitude of inconsistency across studies, an *I* ^2^ statistic of 0%–40% was interpreted to indicate unimportant levels of heterogeneity. An *I* ^2^ statistic of 30%–60% may represent moderate heterogeneity, and *I* ^2^ statistic of 50%–90% may represent substantial heterogeneity. An *I* ^2^ statistic of greater than 75% was interpreted to indicate considerable heterogeneity and was further assessed with subgroup or sensitivity analysis.^28^, ^29^

### 2.7 Grading the body of evidence

Two reviewers (L.P.M.W. and D.E.S.) rated the quality of the evidence and the strength of the recommendations according to the following aspects: study limitations, inconsistency of results, indirectness of evidence, imprecision and publication bias by utilizing the Grading of Recommendations Assessment, Development and Evaluation (GRADE),^30^, ^31^ which provides a systematic approach for considering and reporting each of these factors. An overall rating of confidence in effect estimates was considered critical for the final recommendation.^32^ Any disagreement between the two reviewers was resolved after additional discussion. If a disagreement persisted, then the judgement of a third reviewer (G.A.W.) was decisive.

## 3 RESULTS

### 3.1 Search and selection process

**FIGURE 1:**
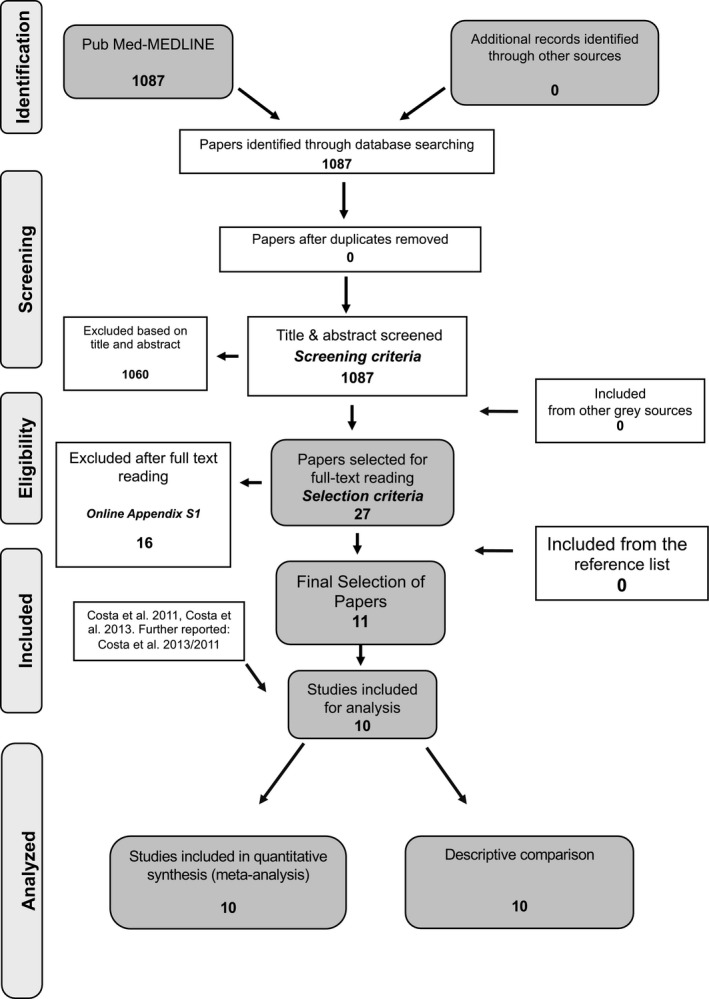
Search and selection results

Searching the MEDLINE‐PubMed and Cochrane databases resulted in 1087 unique papers, as Figure 1 illustrates.

The first screening of the titles and abstracts resulted in 27 papers for which the full papers were obtained. In the second phase, after full‐text reading and contact with the corresponding authors, 16 studies were excluded the reasons for which are presented in online Appendix S1. Three papers do not provide necessary data regarding the overall number of missing teeth, and after contacting the authors, this information could not be retrieved (Wiener et al 2017,^33^ Kapp et al 2007,^34^ Jung et al 2010).^35^ Oliver and Tervonen (1993)^36^ performed only half‐mouth assessments. Three papers that present the number of missing teeth over a period of time were not included (Yoo et al 2019,^37^ Mayard‐Pons et al 2015^38^ and Jimenez et al 2012).^39^ Other reasons for exclusion are found in the table in online Appendix S1. Hand‐searching of the reference list did not reveal any additional papers. Consequently, 11 papers were identified which presented 10 different studies, as data from the paper of Costa et al (2013)^40^ and Costa et al (2011)^41^ concern the same study population.

### 3.2 Assessment of clinical heterogeneity

Considerable heterogeneity was observed among the 10 included studies. Characteristics of study design, study population and diagnostic as well as assessment methods are presented in Table 2. The total number of subjects included in this SR is 29.278, which varies from 92 enrolled participants in Study III^40^ to 12.131 in Study I.^42^ The female gender is more prevalent in seven studies (I, II, IV, VI, VII, VIII and X), and two studies include more males (V and IX).

**TABLE 2:** Overview of the studies processed for data extraction

Selection ID Authors, year, study design, country Risk of bias (Appendix S2)	*N* Type of population	Gender (*N* males, *N* females) Mean age (SD) Range in years	Type of DM and type of assessment	#teeth in patients with DM Total *N* of teeth used for calculations	#teeth in people non‐DM Total *N* of teeth used for calculations
I: Shin et al. 2017 Cross‐sectional Korea RoB: Moderate	Total: 12.131 ♦ DM: 1295 ♦ Non‐DM: 10.836 ♦ Selected from KNHANES, a study periodically conducted by the Korea Centre for Disease Control and Prevention (KCDC), in 2012–2014.	Total ♂ 5342 ♦ ♀ 6789 ♦ Mean age: ? DM ♂:? ♀:? Mean age: ? Non‐DM ♂:? ♀:? Mean age: ?	DM type II *Type of assessment: Prof‐D*	Missing: 7356 ◊ Total teeth: 36.260 ◊ Patient level: 22,3 T+ ♦ 5,7 T‐ ◊ Based on 28 teeth ♦	Missing: 26.331 ◊ Total teeth: 303.408 ◊ Patient level: 25,6 T+ ♦ 2,4 T‐ ◊ Based on 28 teeth ♦
II: Greenblatt et al. 2016 Prospective cohort United States RoB: Low	Total: 9271 DM: 2792 ◊ Uncontrolled: 1324 Controlled: 1468 Non‐DM: 6479 ♦ *Hispanic/Latino population; from the* *Hispanic* *Community Health Study*/*Study of Latinos (HCHS*/*SOL)*.	Total ♂ 6089 ♦ ♀ 9043 ♦ Mean age: ? (18–74) DM ♂:? ♀:? Mean age: ? Non‐DM ♂: ? ♀: ? Mean age: ?	DM type I/II *Type of assessment: Prof‐D*	Missing: 10.140 ◊ Total teeth: 78.176 ◊ Patient level: 24,4 T+ ◊ 3,6 T‐ ◊ Based on 28 teeth ♦ Uncontrolled Missing: 5296 ♦ Total teeth: 37.072 ♦ Patient level: 28 T+ ◊ 4 T‐ ♦ Controlled Missing: 4844 ♦ Total teeth: 41.104 ♦ Patient level: 27,7 T+ ◊ 3,3 T‐ ♦	Missing: 20.733 ◊ Total teeth: 181.412 ◊ Patient level: 24.8 T+ ◊ 3.2 T‐ ♦ Based on 28 teeth ♦
III: Costa et al. 2013/2011 Case‐controlled Brazil RoB: Moderate	Total: 92 ♦ DM: 46 ◊ Poor control: 23 ♦ Well control: 23 ♦ Non‐DM: 46 ♦ Cohort undergoing PMT	Total ♂: 40 ♀: 52 Mean age: ? (22–71) DM ♂ 20 ♀ 26 Mean age: ? Poor control: ♂ 10 ♀ 13 Well control: ♂ 10 ♀ 13 Non‐DM ♂ 20 ♀ 26 Mean age: ?	DM type II *Type of assessment: Prof‐D*	Missing: 225 ◊ Total teeth: 1288 ◊ Patient level: 23,1 T+ ◊ 4,9 T‐ ◊ Based on 28 teeth ♦ Well‐controlled Missing: 96 ◊ Total teeth: 644 ◊ Patient level: 23,8 T+ ◊ 4,2 T‐ ◊ Poorly controlled Missing: 129 ◊ Total teeth: 644 ◊ Patient level: 22,4 T+ ◊ 5,6 T‐ ◊	Missing: 183 ◊ Total teeth: 1288 ◊ Patient level: 24 T+ ◊ 4 T‐ ◊ Based on 28 teeth ♦
IV: Patel *et al*. 2013 Cross‐sectional United States RoB: Serious	Total: 2055 ♦ DM: 384 ♦ Non‐DM: 1671 ♦ *General population from the NHANES sample*	Total ♂ 992 ♦ ♀ 1063 ♦ Mean age: ? DM ♂: ? ♀: ? Mean age: ? Non‐DM ♂: ? ♀: ? Mean age: ?	DM type I/II *Type of assessment: Self‐R*	Missing: 3763 ◊ Total teeth: 10.752 ◊ Patient level: 18,2 T+ ◊ 9,8 T‐ ♦ Based on 28 teeth ♦	Missing: 11.196 ◊ Total teeth: 46.788 ◊ Patient level: 21,3 T+ ◊ 6,7 T ‐♦ Based on 28 teeth ♦
V: Botero et al. 2012 Cross‐sectional Colombia RoB: Moderate	Total: 124 ◊ With DM: 65 ♦ Selected from the Hospital Universitario San Vicente de Paul (Medellin, Colombia) Non‐DM: 59 ◊ Selected from the School of Dentistry at the Universidad del Valle	Total ♂: 67 ◊ ♀: 57 ◊ Mean age: ? DM ♂: 45 ♦ ♀: 20 ♦ Mean age: 57,4 ◊ Non‐DM ♂: 22 ◊ ♀: 37 ◊ Mean age: 44,1 ◊	DM type I/II *Type of assessment: Prof‐D*	Missing: 481 ◊ Total teeth: 1820 ◊ Patient level: 20,6 T+ ◊ 7,4 T‐ ◊ Based on 28 teeth ♦	Missing: 112 ◊ Total teeth: 1652 ◊ Patient level: 26.1 T+ ◊ 1.9 T‐ ◊ Based on 28 teeth ♦
VI: Sensorn et al. 2012 Cross‐sectional Thailand RoB: Serious	Total: 605 ♦ DM: 379 ♦ Non‐DM: 226 ♦ *General population living in Nachaluay district, Ubonratchathani,* *Thailand*.	Total ♂ 130 ◊ ♀ 475 ◊ Mean age: ? (20–86) ♦ DM ♂: 72 ♦ ♀: 307 ♦ Mean age: 54,7 ♦ Non‐DM ♂: 58 ♦ ♀: 168 ♦ Mean age: 43,6 ♦	DM type I/II *Type of assessment: Prof‐D*	Missing: 2414 ◊ Total teeth: 12.128 ◊ Patient level: 25,63 T+ ◊ 6,37 T‐ ◊ Based on 32 teeth ♦	Missing: 694◊ Total teeth: 7232◊ Patient level: 28,93 T+ ◊ 3,07 T‐ ◊ Based on 32 teeth ♦
VII: Kaur et al. 2009 Cross‐sectional Germany RoB: Low	Total: 4288 ◊ DM: 327 ◊ DM I: 145 ♦ DM II: 182 ♦ Non‐DM: 3961◊ *General population from the SHIP Trend study (population‐based survey in North‐Eastern Germany)* *TIDM: Centre of Cardiology and Diabetes,* *Karlsburg*	Total ♂: 2055 ◊ ♀: 2233 ◊ Mean age: ? DM ♂: 180 ◊ ♀: 147 ◊ Mean age: 52,5 ◊ Type I ♂: 76 ♦ ♀: 69 ◊ Mean age: 37,4 ♦ Type II ♂: 104 ♦ ♀: 78 ◊ Mean age; 64,5 ♦ Non‐DM ♂: 1875 ◊ ♀: 2086 ◊ Mean age: 46,8 ◊	DM type I/II (data are presented per type) *Type of assessment: T1DM Prof‐D* *SHIP/T2DM: Self‐R* *Type of assessment:* *Self‐R (T1DM) and/or Prof‐D (T2DM)*	Missing: 3414 ◊ Total teeth: 9156 ◊ Patient level: 18 T+ ◊ 10 T‐ ◊ Based on 28 teeth ♦ DM type I Missing: 885 ◊ Total teeth: 4060◊ Patient level: 21,9 present teeth ◊ 6,1 missing teeth ♦ DM type II Missing: 2530 ◊ Total teeth: 5096◊ Patient level: 14,1 T+ ◊ 13,9 T‐ ♦	Missing: 282.184◊ Total teeth: 110.908 ◊ Patient level: Non‐DM (compared DMI&II) 19,9 T+ ◊ 8,1 T ‐◊ Based on 28 teeth ♦ Non‐DM (compared DM I) Missing: 13.764 ◊ Total teeth ever: 74.116◊ Patient level: 22,8 present teeth ◊ 5,2 missing teeth ♦ Non‐DM (compared DM II) Missing: 14.454◊ Total teeth: 36.792◊ Patient level: 17,0 T+ ◊ 11,0 T‐ ♦
VIII: Patiño‐Marín et al. 2008 Cross‐sectional Mexico RoB: Moderate	Total: 70 ◊ DM: 35 ◊ Non‐DM: 35 ◊ *General population in Mexico*.	Total ♂: 33 ◊ ♀: 46 ◊ Mean age: ? DM ♂: 14 ◊ ♀: 21 ♦ Mean age: 45 ♦ Non‐DM ♂: 19 ◊ ♀: 25 ♦ Mean age: 42 ♦	DM type II *Type of assessment: Prof‐D*	Missing: 200 ◊ Total teeth: 980 ◊ Patient level: 22,3 T+ ◊ 5,7 T‐ ♦ Based on 28 teeth ♦	Missing: 123 ◊ Total teeth: 980 ◊ Patient level: 24,5 T+ ◊ 3,5 T‐ ♦ Based on 28 teeth ♦
IX: Bacic et al. 1989 Cross‐sectional Croatia RoB: Serious	Total: 411 ◊ DM: 222 ♦ Insulin dependent (IDDM): 109 ♦ Non‐insulin dependent (NIDDM): 113 ♦ *DM patients*: *selected from the Vuk Vrhovac Institute of Diabetes*, *Endocrinology and Metabolic Diseases in Zagreb referred from all parts of Croatia*. Non‐DM: 189 ♦ *Non*‐*DM patients*: *general population of Croatia during a survey on the prevalence of periodontal disease and caries in Croatia*.	Total ♂: 245 ◊ ♀: 166 ◊ Mean age: ? DM: ♂: 130 ♦ ♀: 92 ♦ Mean age: 49,6 ♦ Non‐DM: ♂: 115 ♦ ♀: 74 ♦ Mean age: 43,9 ♦	DM type I/II *Type of assessment: Prof‐D*	Missing: 2731 ◊ Total teeth: 7104 ◊ Patient level: 19,7 T+ ◊ 12,3 T‐ ♦ Based on 32 teeth ♦	Missing: 1833 ◊ Total teeth: 6048 ◊ Patient level: 22,3 T+ ◊ 9,7 T‐ ♦ Based on 32 teeth ♦
X: Falk et al. 1989 Cross‐sectional Sweden RoB: Moderate	Total: 231 ◊ DM: 154 ◊ Long duration 82 (28,9 years) Short duration: 72 (5,2 years) *Selected from the Department of medicine at the central Hospital in Jönköping*, *Sweden*. Non‐DM: 77 ♦ *selected from the county council's register of persons residing in the borough of Jonkoping*. *General population*	Total ♂: 112 ◊ ♀: 119 ◊ Mean age: ? DM ♂: 78 ◊ ♀: 76 ◊ Mean age: ? (20–70) ♦ Long duration ♂: 40 ♦ ♀: 42 ♦ Short duration ♂: 38 ♦ ♀: 34 ♦ Non‐DM ♂ 34 ♀ 43 Mean age: ?	DM type I/II *Type of assessment:?*	Missing: 1007 ◊ Total teeth: 4312 ◊ Patient level: 21,4 T+ ◊ 6,6 T‐ ◊ Based on 28 teeth ♦ Long duration Missing: 467 ◊ Total teeth: 2296 ◊ Patient level: 22,3 T+ ♦ 5,7 T‐ ◊ Short duration Missing: 540 ◊ Total teeth: 2016 ◊ Patient level: 20,5 T+ ♦ 7,5 T‐ ◊	Missing: 416◊ Total teeth: 2156◊ Patient level: 22.6 T+ ♦ 5.4 T‐ ◊ Based on 28 teeth ♦

Abbreviations: ?, Is not reported/unknown; ◊, Calculated; ♦, Given by the original author; PMT, Periodontal maintenance therapy; PrDM, Previous known diabetes mellitus; Prof D, Professionally diagnosed; RoB, Risk of bias; ScDM, Screening detected diabetes mellitus; Self‐R, Self‐reported; T‐, Missing teeth; T+, Present teeth; T1DM, Type 1 diabetes; T2DM, Type 2 diabetes; Type I and/or II, Distinction is made between diabetes type I and II; Type I/II, No distinction is made between type of diabetes.

One case‐control study makes an effort to match the gender distribution (III). The population in Study II^43^ is a specific ethnic group (Hispanics or Latinos). Studies originating from the following world continents are present: Europe (VII,^44^ IX^45^ and X^46^), North America (II,^43^ IV,^47^ and VIII^48^), Asia (I^42^ and VI^49^) and South America (III^40^ and V ).^50^ All studies include a non‐DM group in satisfactory general health who were drawn from the population of the country where the study was performed. The DM participants in Studies IX^45^ and X^46^ were specifically selected from a central hospital or institute for metabolic diseases. For inclusion in the individual studies, criteria and diagnoses were utilized regarding DM status: self‐reported (IV^47^) and clinically assessed DM (I,^42^ II,^43^ III,^40^ V,^50^ VI,^49^ VIII^48^ and IX).^45^ The clinical assessments were performed by different methods, such as fasting plasma glucose (FPG), glucose or HbA1c levels. Study VII^44^ reports DM based on both clinical assessments and self‐reports. In one paper, it was unclear how the DM status had been assessed (X).^46^

In total, three studies specifically focus on DM type II (I,^42^ III^40^ and VIII).^48^ One paper differentiates between types I and II (VII).^44^ For the overall calculations, data from these groups were merged, while for the subgroup analysis, the original group data were employed. Originally, Study VIII^48^ made this distinction, but as the type I DM group included children, this group was consequently excluded from data extraction and only the data on type II DM patients were utilized. Two studies (II^43^ and III^40^) report data on the DM group about well‐ and poorly controlled individuals. Smokers among non‐DM individuals were separately analysed in Study V^50^, and as none of the DM patients reported smoking, only the non‐smoking, non‐DM individuals were considered as a control group. Other characteristics concerning DM include short or long duration of DM (X^46^), insulin independence (IX^45^) and diagnosis of DM known beforehand or assessed on the spot.

### 3.3 Assessment of methodological heterogeneity

Eight of the included observational studies utilize a cross‐sectional design (I,^42^ IV,^47^ V,^50^ VI,^49^ VII,^44^ VIII,^48^ IX^45^ and X^46^), one is a prospective cohort (II^43^), and one is a retrospective case‐control (III).^40^ Two included papers employ data from national databases: NHANES and KNHANES (I^42^ and IV^47^), and two papers utilize data from a national study: NFBC‐1966, SHIP and HCHS/SOL (VII^44^ and II).^43^ Study III^40^ includes patients who were enrolled in a periodontal maintenance programme. The number of evaluated teeth is 32 in two studies (VI^49^ and IX^45^) and 28 in eight studies (I,^42^ II,^43^ III,^40^ IV,^47^ V,^50^ VII,^44^ VIII^48^ and X).^46^

### 3.4 Methodological quality assessment

A summary of the methodological quality and potential risk‐of‐bias scores is presented in Table 3. Detailed quality assessment for each included study is provided in online Appendix S2.

**TABLE 3:** Summary of the risk‐of‐bias assessment using Robins‐E tool

Study ID	Bias due confounding	Bias in selection of participants into the study	Bias in classification of exposures	Bias due to departures from intended exposures	Bias due missing data	Bias in measurement of outcomes	Bias in selection of the reported result	Overall risk of bias	For details, see online appendix
I	Moderate	Low	Low	Moderate	Low	Low	Low	**Moderate**	S2‐2
II	Low	Low	Low	Low	Low	Low	Low	**Low**	S2‐3
III	Moderate	Moderate	Low	Low	Low	Low	Low	**Moderate**	S2‐4
IV	Low	Low	Moderate	Serious	Moderate	Low	Low	**Serious**	S2‐5
V	Moderate	Moderate	Moderate	Moderate	Low	Low	Low	**Moderate**	S2‐6
VI	Low	Serious	Serious	Moderate	Low	Low	Low	**Serious**	S2‐7
VII	Low	Low	Low	Low	Low	Low	Low	**Low**	S2‐8
VIII	Moderate	Moderate	Low	Low	No information	Low	Low	**Moderate**	S2‐9
IX	Serious	Moderate	Low	Moderate	Low	Low	Moderate	**Serious**	S2‐10
X	Moderate	Low	Moderate	Moderate	Low	Low	Low	**Moderate**	S2‐11

Based on a summary of the bias assessment domains, the estimated potential risk of bias is low for two studies: II^43^ and VII^44^; moderate for the majority of the studies: I,^42^ III,^40^ V,^50^ VIII^48^ and X^46^; and serious for the remaining three studies: IV,^47^ VI^49^ and IX.^45^

### 3.5 Study results

From the included studies, the overall DM population consisted of 5699 patients and the non‐DM controls of 23.579 individuals. The overall prevalence of DM in the included cross‐sectional studies is 16.8%.

#### 3.5.1 Description of findings

Table 4 describes and summarizes the statistical differences as reported in the original studies between DM patients and non‐DM individuals with regard to the number of missing teeth.

**TABLE 4:** A descriptive summary of statistical significance levels of the difference between DM patients compared to non‐DM with regard to number of teeth

Study	Exposure	Number of teeth significance	Comparison
1. Shin et al 2017	DM	?	non‐DM
2. Greenblatt et al 2016	DM	?	non‐DM
3. Costa et al 2011/2013	DM	+	non‐DM
4. Patel et al 2013	DM	+	non‐DM
5. Botero et al 2012	DM	+	non‐DM
6. Sensorn et al 2012	DM	+	non‐DM
7. Kaur et al 2009	DM	?	non‐DM
8. Patiño‐Marín et al 2008	DM	+	non‐DM
9. Bacic et al 1989	DM	+	non‐DM
10. Falk et al 1989	DM	?	non‐DM
**Total**		6/10 have significant less teeth 0/10 no significant difference 4/0 do not specified	

?, unclear/not specified; 0, no difference; +, DM patients have significantly less teeth than non‐DM.

From the 10 overall comparisons, six provide data and indicate significantly more tooth loss for the DM patients. Four of the included studies do not specify or are unclear whether any statistical differences between the DM and non‐DM controls were present.

#### 3.5.2 Meta‐analysis

**FIGURE 2:**
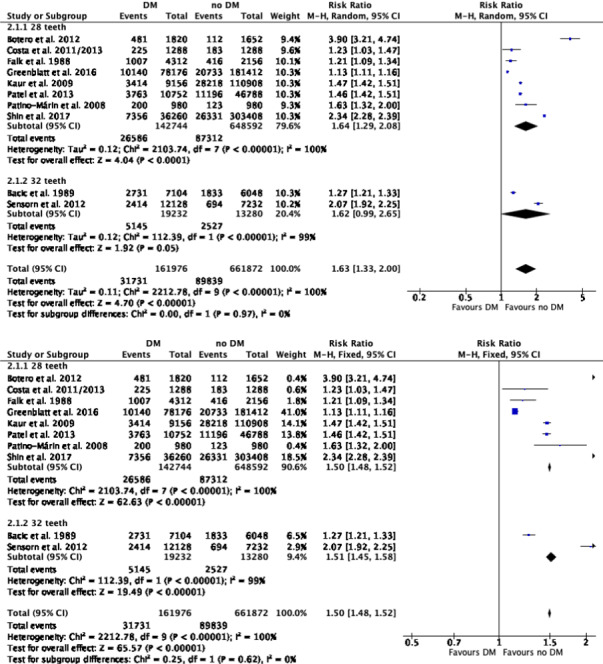
(2.1) Meta‐analysis evaluating the effect of DM compared to non‐DM on tooth loss using a random model: overall and evaluable number of teeth, 28/32 teeth. (2.2) Meta‐analysis evaluating the effect of DM compared to non‐DM on tooth loss using a fixed model: overall and evaluable number of teeth, 28/32 teeth

The results indicate a higher probability (RR = 1.63) of tooth loss for patients with DM as compared to non‐DM individuals. This is based on the 10 included studies with a 95% CI (1.33; 2.00, *p* < 0.00001) and shown in Figure 2. The subgroup analysis based on studies that provide data relative to 32 evaluable teeth reveals an RR of 1.51 with a 95% CI (1.45; 1.58, *p* < 0.00001), and for those evaluating 28 potential teeth, the RR was 1.64 with a 95% CI (1.29; 2.08, *p* < 0.0001).

Tables 5 and 6 summarize the detailed data of the outcomes of the meta‐analysis and the subgroup analysis including the RR, 95% CI and *p*‐value. Online Appendix S3 presents the corresponding forest plots. Due to a lack of data, it was not possible to perform further sub‐analysis on DM details such as insulin dependence and DM duration.

**TABLE 5:** Overview (sub) analysis overall and evaluable number of teeth (28/32)

	Included studies	Effect sizes				Heterogeneity		Funnel plot appendix	For details, see
		RR	Model	95% CI	*p*‐Value	*I* ^2^ value	*p*‐Value		
Overall	10 studies	1.63	Random	[1.33–2.00]	<0.00001	100%	<0.00001	S4	Figure 2
Number of teeth									
32 teeth	Bacic et al 1989 Sensorn et al 2012	1.51	Fixed	[1.45–1.58]	<0.00001	99%	<0.00001	S4	Figure 2
28 teeth	Botero et al 2012 Costa et al 2011/2013 Falk et al 1989 Greenblatt et al 2016 Kaur et al 2009 Patel et al 2013 Patiño‐Marín et al 2008 Shin et al 2017	1.64	Random	[1.29–2.08]	<0.0001	100%	<0.00001	S4	Figure 2

**TABLE 6:** Overview sub‐analysis: risk of bias, study design, world continent, DM type II and DM status

	Included studies		Effect sizes				Heterogeneity		Funnel plot	For details, see
			RR	Model	95% CI	*p*‐Value	*I* ^2^ value	*p*‐Value		
Risk of bias										
Low	Greenblatt et al. 2016 Kaur et al. 2009		1.22	Fixed	[1.20–1.24]	<0.00001	100%	<0.00001	S4	S3‐1
Moderate	Botero et al 2012 Falk et al. 1989 Patiño‐Marín et al. 2008 Costa et al. 2013/2011 Shin et al. 2017		1.85	Random	[1.27–2.71]	0.001	98%	<0.00001	S4	S3‐1
Serious	Patel et al. 2013 Bacic et al. 1989 Sensorn et al. 2012		1.48	Fixed	[1.45–1.52]	<0.00001	98%	<0.00001	S4	S3‐1
Study design										
Cross‐sectional	Botero et al 2012 Falk et al. 1989 Kaur et al. 2009 Patel et al. 2013 Patiño‐Marín et al. 2008 Shin et al. 2017 Bacic et al. 1989 Sensorn et al. 2012		1.77	Random	[1.44–2.17]	<0.00001	99%	<0.00001	S4	S3‐2
World continent										
Europe		Kaur et al. 2009 Bacic et al. 1989 Falk et al. 1989	1.39	Fixed	[1.35–1.42]	<0.00001	94%	<0.00001	S4	S3‐3
North America		Greenblatt et al. 2016 Patel et al. 2013 Patiño Marín et al. 2008	1.22	Fixed	[1.20–1.24]	<0.00001	99%	<0.00001	S4	S3‐3
Asia		Shin et al. 2017 Sensorn et al. 2012	2.30	Fixed	[2.25–2.36]	<0.00001	88%	0.004	S4	S3‐3
South America		Costa et al. 2013/2011 Botero et al. 2012	2.27	Fixed	[2.00–2.58]	<0.00001	99%	<0.00001	S4	S3‐3
Solely diagnosed as DM type II										
Type II		Shin et al. 2017 Costa et al. 2011/2013 Kaur et al. 2009 Patino‐Marin et al. 2008	1.56	Random	[1.02–2.39]	0.04	100%	<0.00001	S4	S3‐4
Diabetic status										
Well‐controlled vs. non‐DM		Greenblatt et al. 2016 Costa et al. 2011/2013	1.03	Fixed	[1.00–1.06]	0.04	0%	0.88	S4	S3‐5
Poorly controlled vs. non‐DM		Greenblatt et al. 2016 Costa et al. 2011/2013	1.25	Fixed	[1.22–1.29]	<0.00001	23%	0.25	S4	S3‐5
Poorly vs. well‐controlled DM		Greenblatt et al. 2016 Costa et al. 2011/2013	1.21	Fixed	[1.17–1.26]	<0.00001	0%	0.41	S4	S3‐5

The subgroup analysis on risk of bias for those studies revealed an estimated low risk with an RR of 1.22 and a 95% CI (1.20; 1.24, *p* < 0.00001), an RR of 1.85 with a 95% CI (1.27; 2.71, *p* = 0.001) for those with a moderate risk and an RR of 1.48 at a 95% CI (1.45; 1.52, *p* < 0.00001) for those with a serious risk (for details, see online Appendix S3.1). When only studies that were originally designed as cross‐sectional evaluations were considered, the RR was 1.77 at a 95% CI (1.44; 2.17, *p* < 0.00001; for details, see online Appendix S3.2).

A subgroup analysis on the world continent in which the study was performed resulted in a RR for Europe of 1.39 at a 95% CI (1.35; 1.42, *p* < 0.0001), North America 1.22 at a 95% CI (1.20; 1.24, *p* < 0.00001), Asia 2.30 at a 95% CI (2.25; 2.36, *p* < 0.00001) and South America 2.27 at a 95% CI (2.00; 2.58, *p* < 0.00001). For all continents, the risk for tooth loss in DM patients was higher as compared to non‐DM individuals (for details, see online Appendix S3.3).

Only Study VII^44^ presents usable data for a DM type I group, and therefore, no specific subgroup analysis could be performed.^17^ For the studies that solely evaluate DM type II, the RR for tooth loss was 1.56 at a 95% CI (1.02; 2.39, *p* = 0.04; for details, see online Appendix S3.4).

Furthermore, a subgroup analysis on DM status was performed. No significant difference was found regarding tooth loss when well‐controlled DM patients were compared to non‐DM individuals, as demonstrated by the RR: 1.03 with a 95% CI of 1.00 to 1.06 (*p* = 0.04). A higher risk of tooth loss in poorly controlled DM patients was found when compared to non‐DM individuals (RR = 1.25 with a 95% CI of 1.22 to 1.29 (*p* < 0.00001)) and also when compared to well‐controlled DM patients (RR = 1.21 with a 95% CI of 1.17 to 1.26 (*p* < 0.00001)); for details, see online Appendix S3.5.

Sensitivity analyses were performed by evaluating the effect of excluding studies based on specific aspects in the domain of clinical or methodological characteristics. Sensitivity analysis revealed no differences in the RR compared to the overall RR as judged based on overlapping 95% CIs, indicating that the overall analysis was robust.

#### 3.5.3 Statistical heterogeneity

Considerable heterogeneity was observed in the meta‐analyses; for details, see Tables 5 and 6.

This implies a variation between studies due to heterogeneity rather than chance. To explore heterogeneity, a subgroup analysis was performed to attempt to explain the variation in effects. Subgroup analysis on the evaluated number of teeth, either 28 or 32, revealed an overlap for the 95% CI and with the overall 95% CI. By performing the chi‐square test and *I* ^2^, considerable heterogeneity was apparent and varied between 99% and 100%. Subgroup analysis by world continent indicated considerable heterogeneity per continent, ranging from 88% to 99%. Additionally, the meta‐analysis of studies solely evaluating DM type II presented considerable (100%) heterogeneity. The three sub‐analyses on DM status did not demonstrate important heterogeneity, and the *I* ^2^ statistics were low (0%–23%). Subgroup analysis of only studies with an estimated low risk of bias or analyses of studies that were based on an original cross‐sectional design illustrates that the *I* ^2^ statistic remains high. It is therefore unclear based on the subgroup and sensitivity analysis what the driver of the high statistical heterogeneity is, although it provides an indication that DM status could be a factor.

### 3.6 Publication bias

Testing for publication bias was possible for the overall analysis, which is presented in Appendix S4. The funnel plot reveals that almost all outcomes are located at the top of the funnel, suggesting that no studies concerning small populations were included. Furthermore, the distribution is asymmetrical around the overall value. Consequently, it is presumed that a potential risk for publication bias may exist.

### 3.7 Evidence profile

Table 7 presents a summary of the factors employed to establish the body of evidence profile according to GRADE (2014)^20^ relative to the magnitude of the risk for tooth loss. In summary, this SR is based on 10 observational studies (Figure 1) and the potential risk of bias was estimated as low to serious (Table 3 and Appendix S2). Because data from studies were derived from different populations and world continents, the findings are considered to be generalizable. Based on the heterogeneity between the included studies, data were judged to be rather inconsistent (see Table 2). The data were considered to be rather precise, because all selected studies focused on tooth loss as a primary outcome and because the majority reveal an overlap in the overall 95% CI (see Figure 2, Tables 5 and 6 and online Appendix S3). As publication bias may be present and the funnel plots indicate that outcomes could be overestimated, the presence of reporting bias is likely. The interpretation of the overall RR being 1.63 is that it concerns a small effect.^51^ Considering all GRADE aspects, the evidence profile that emerges from this review is that the strength is moderate.

**TABLE 7:** GRADE evidence profile for the number of teeth and risk ratio among DM as compared to non‐DM

Summary of findings table on the body of the estimated evidence profile	
Determinants of quality	Risk ratio
Study design (Table 2)	Observational studies
#studies (Figure 1) #comparisons	#10 #10
Risk of bias (Table 3, Appendix S2)	Low to serious
Consistency (Table 2)	Rather inconsistent
Directness	Rather generalizable
Precision (Figure 2, Tables 5 and 6 Online Appendix S3)	Rather precise
Reporting bias	Likely
Magnitude of the effect (Figure 2, Tables 5 and 6 Online Appendix S3)	Small
Strength of the recommendation based on the quality and body of evidence	Moderate
Direction of recommendation	**With respect to tooth loss, there is moderate certainty for a small risk for DM over non‐DM**

## 4 DISCUSSION

The present review summarizes the available body of dental and medical literature with respect to an important question that examines the association between DM and tooth loss. The results of this study indicate a higher probability (RR = 1.63) of tooth loss for patients with DM as compared to non‐DM individuals. This appears to align with what is reported in other epidemiologic studies, as several have supported the link between DM, periodontal diseases and dental caries.^52^, ^53^ These are the two most common reasons for the endpoint parameter of tooth loss.

### 4.1 Selection choices made

The selection process of the included papers of this SR deviates from the traditional Cochrane approach.^17^ However, the foundation is based on similar principles. A two‐step approach was utilized: first, screening of titles and abstracts was performed; second, more specific inclusion criteria were implemented to ensure that the only studies included presented data about tooth loss among DM patients and non‐DM individuals as the primary outcome. The reviewers are aware that there may be additional information available where data on diabetic status and number of teeth are retrieved from reported demographic data and presented as an interesting result.^54^, ^55^, ^56^ Inclusion of these data may introduce a reporting bias that affects the conclusion drawn^57^; therefore, it was specifically prespecified that primary outcomes from the study protocol should be included in the final data presentation. The inclusion of reported outcomes should not be based on a selection of results that were not the primary focus of the study.^58^ From a statistical perspective, the sample size of the included studies should have been driven by the primary outcome, which positively affects the power. Consequently, for the present SR, only papers with tooth loss and DM as the primary focus of the original study were sought, and these two aspects had to be mentioned as the aim in the abstract or title. With this approach, it was considered that the most reliable and valid estimation of the RR was obtained.

### 4.2 Diabetes mellitus comorbidities

For this SR, only DM without reported comorbidities was considered. Papers on participants with other systemic diseases were excluded^59^, ^60^ to avoid bias in the observed association between DM and tooth loss. However, DM has many risk factors, such as age, overweight and obesity, inactivity, habitual smoking, food intake, socio‐economic status, family history of DM, geographical region and blood pressure.^61^ The included papers did not adjust for these factors. Only in one paper (V^50^) was smoking specifically mentioned: none of the DM patients reported being smokers, and only non‐smoking non‐DM individuals were considered as a control group. A range of predictors for tooth loss in periodontitis patients has been reported. A recent SR assesses the consistency and magnitude of different predictors, concluding that age, non‐compliance, smoking, DM, teeth with bone loss, high probing pocket depth, mobility and molars, especially with furcation involvement, demonstrate a higher risk of tooth loss.^16^ Considering the above, there appears to be an overlap of potential causal components for tooth loss in diabetics and periodontitis with the following factors: age, smoking habit and diabetic status. In future studies, it is recommended to include these factors in the analysis. Because the eligible studies of the present review did not report or take these into consideration, the reported outcome allows only for the interpretation of an unadjusted effect size. From the obtained observational data, it is also not possible to make causality claims. As stated earlier, geographical region, gender, type of DM and type of assessment may interfere in the DM and tooth loss association.

### 4.3 Reporting bias

The main origin of publication bias is failure to publish negative outcomes or null findings. Additionally, it is more difficult to publish papers in which no differences between groups are found.^29^, ^62^ The consequences are that this may lead to overestimation of exposure as deducted based on the meta‐analyses.^63^ The present funnel plot (see online Appendix S4) illustrates that almost all outcomes were located at the top of the funnel, suggesting that relatively few small studies were included. The usage of a strict inclusion criteria may explain this specific distribution. It is recognized that studies with small sample sizes that fail to establish a difference between groups either have not been published or have difficulties in being published in impact factor journals.^62^

### 4.4 Type of diabetes

As prediabetes may be reversible,^64^ data from these participants were not considered, as only one study (II^43^) was available. Gestational diabetes consists of high blood glucose only during pregnancy^65^ and was consequently not analysed in the present review. Type I diabetes can develop at any age but occurs most frequently in children and adolescents. However, type II DM is more common in adults and accounts for approximately 90% of all diabetes cases.^66^ Three of the included studies specifically focus on DM type II (I,^42^ III^40^ and VIII^48^). Only one paper (VII^44^) differentiates between types I and II. It was therefore not possible to perform a subgroup analysis to compare types I and II in this dataset. Analysis focused on DM type II, for which a RR of 1.56 for the risk of tooth loss was found. However, the relationship between DM type II and tooth loss is complicated by the fact that the disease onset generally occurs in middle and late ages, coinciding with the time that periodontitis becomes more prevalent.^44^ Nevertheless, studies focusing on type I DM patients also indicate an increased risk of periodontitis compared to non‐DM individuals. Study VIII^48^ includes children, and this group was consequently excluded because children can have temporary, mixed or permanent dentition.

Considerable heterogeneity was observed in the outcomes of most sub‐analyses; however, sub‐analysis on diabetes type II did not provide an explanation for the high level of heterogeneity. Only the subgroup analysis on diabetic status being either poorly or well‐controlled revealed a low level of statistical heterogeneity (0%–23%). This could indicate that diabetic control is an aspect that contributes to heterogeneity among study outcomes. However, this sub‐analysis was based on only two studies that had similar populations and study designs. Because this study's meta‐analyses indicated a heterogeneity in the outcome, the reader should exercise caution in utilizing the RR as the exact measure of the risk for tooth loss.

### 4.5 Type of assessment

The Centers for Disease Control and Prevention have estimated that among US individuals, DM is underdiagnosed, which implies that participants in the included studies may have been unaware of their positive DM status.^65^, ^67^ In that case, it would affect the non‐DM group, as these may potentially include DM patients, which thus could result in an underestimation of the effect size. Future research in relation to metabolic status should therefore preferably utilize only those participants who have been clinically diagnosed as DM or non‐DM. The majority of the included studies (8 of 10) performed a clinical assessment for DM. Two included studies employed a questionnaire or self‐report for DM status. The value of this self‐report of disease in relation to medical records has been demonstrated to have high (>90%) specificity but low sensitivity (66%) for DM.^68^

### 4.6 Evaluable number of teeth

The number of evaluable teeth was assessed by professionally performed oral examinations to obtain optimally reliable values. Two studies that report the number of teeth by utilizing a questionnaire were therefore, in the second phase, excluded.^69^, ^70^ However, both indicate numerically more missing teeth in the DM group as compared to healthy individuals.

Two of the included studies employ data based on 32 evaluable teeth and therefore include wisdom teeth (IX^45^ and VI^49^), while the other eight evaluate 28 teeth. A subgroup analysis was performed with regard to the number of evaluated teeth. There was a numerical difference in RR of tooth loss between those studies evaluating 28 and 32 teeth (1.64 and 1.51, respectively), although the 95% CIs overlap ([95% CI 1.29; 2.08] and [95% CI 1.45; 1.58], respectively; see Figure 2 and Table 5). Therefore, the difference of 0.13 between the RRs does not appear to be significant. Because of this lack of statistical difference for the other sub‐analyses, the data from studies with either 28 or 32 evaluable teeth were not separated (see Table 6 as well as online Appendices S3‐1 and S3‐5). In the cases in which wisdom teeth are included in the evaluation, prophylactic removal should be considered as a reason for extraction. This aspect was not analysed in the selected studies that evaluate 32 teeth. The numerically lower but non‐significant difference in the analyses of 32 and 28 teeth could be influenced by this. The RR in the sub‐analysis with 32 teeth was lower than those studies that evaluate 28 teeth. The lower association with DM could be, in part, the result of prophylactic removal.

### 4.7 Geographical region

From the included cross‐sectional studies, the prevalence of DM is 16.8%. The World Health Organization (WHO) published in 2016^71^ the global DM prevalence as 9.2% for adults ≥18 years. This indicates that the data derived from the included studies are skewed towards DM, which in effect may provide an overestimation of the risk of tooth loss. A recent SR reports the prevalence of DM among subjects with periodontitis by continent. It indicates that the highest prevalence of DM was observed in studies from Asian countries (17.2%) and the lowest for those from Europe (4.3%).^23^ In the present review, sub‐analysis of the risk of tooth loss due to DM by world continent also demonstrates numerical differences. Asia (RR: 2.30) had the highest risk, followed by South America (RR: 2.27). The 95% CI of the RR of these two continents did not overlap with those of North America (RR: 1.22) or Europe (RR: 1.39), as both have a lower risk. Apart from comparable differences in the prevalence of DM, the differences in RR per region cannot readily be explained. What could contribute to the findings is that Asians are particularly susceptible to periodontitis^72^ and that DM is found to be more prevalent compared to other ethnic groups.^73^, ^74^ The presumed relationship between DM and severity of periodontitis may then be seen as a possible explanation for the relatively high RR. However, no such explanation is available for the higher RR of tooth loss in South America. Study II^43^ evaluates a specific ethnic group (Hispanics or Latinos) and reports an RR that is lower than the overall RR of the present SR (1.13), which seems to be in line with Arora et al,^75^ who compared several ethnic groups in terms of oral health, lifestyle and usage of dental services in the United Kingdom. Individuals belonging to the non‐White groups were less likely to report dental extractions and to have fewer than 20 teeth. This may reflect genuinely better oral health. The latter appears to explain the majority of the reduced risk found in Study II.^43^ However, a study from the United States^76^ suggests that Black individuals are more likely to choose dental extractions. This is mainly explained by preference, treatment acceptability and ability to afford treatment. A recent SR reports no difference for mean annual tooth loss when comparing geographical groups of North America, Europa, Japan and Oceania versus South America and Asia.^77^ Altogether, the above suggests that racial disparities could influence the observed tooth loss, although no clear explanation can be provided for the range in results as observed in the sub‐analysis by geographical region.

### 4.8 Gender

Seven of the included papers feature more females than male participants, while DM type II is more common in males than females.^78^ Females generally have a greater knowledge and more positive attitude than males towards oral health behaviour.^79^ This is associated with a reduced risk for the progression and severity of periodontitis.^80^ The skewed gender distribution towards females could cause underestimation of the outcome for this SR.

### 4.9 Risk of bias

Assessment of risk of bias is a key step in conducting SRs and informs many other steps and decisions within the review. It also plays an important role in the final assessment of the strength of the evidence.^81^ Sub‐analysis based on the overall estimated risk of bias of the selected studies indicates that for low risk of bias, a smaller RR (1.22 and 95% CI [1.20; 1.24]) was found than for those with a serious risk (RR = 1.48 at a 95% CI [1.45; 1.52]). The confidence interval for both low and serious risk of bias was small, which suggests that the estimate is not flawed by imprecision. If the review was restricted to only high methodological quality and low‐risk‐of‐bias studies, then the synthesis of the data concerning the number of teeth in DM patients as compared to non‐DM individuals would indicate that the RR for tooth loss is rather small.

### 4.10 limitations & direction for further research

#### 4.10.1 Limitations

● The language restriction to English resulted in three potential studies that had to be excluded. Two were in Spanish,^82^, ^83^ and one was in Hungarian.^84^ Based on the information provided in the English abstract, it appears that in these three studies, tooth loss was greater among DM patients as compared to non‐DM individuals. These results corroborate the present findings.
● Caries and periodontitis are the predominant reasons for tooth loss. None of the included studies provided details that could help discern what the indications for extraction had been.
● Factors such as differentiation between DM types I and II, type of assessment (self‐report or professional), gender and age may have influenced the heterogeneity. This could not be further analysed due to a lack of complete descriptions of the population included in the original studies.
● To summarize data from different geographical regions, it was decided to perform subgroup analysis on world continents. The reader should be aware that the reported studies may not capture the true RR of a specific world continent. Some studies have sampled only from small geographical regions, which may not represent the population of the continent.^23^

#### 4.10.2 Directions for further research

Despite these limitations, this SR is meaningful and indicates a higher level of tooth loss in DM patients. However, outcomes on age and smoking habits shall be considered in future research.

## 5 CONCLUSION

There is moderate certainty evidence for a small but significant higher risk of tooth loss in DM patients as compared to those without DM. Subgroup analysis showed that this was also higher if only DM type II was considered. If the data were separated by the world continent where the study was performed, analysis showed that the magnitude of the risk was particularly higher in Asia and South America.

## 6 CLINICAL RELEVANCE

### 6.1 Scientific rationale for the study

Diabetes mellitus (DM) is a chronic inflammatory disease. Evidence supports an increased risk for periodontal diseases and incidence/severity of caries in DM patients. Both are primary sources of tooth loss. It has not been systematically being reviewed whether DM is associated with a higher risk of tooth loss compared to non‐DM individuals.

### 6.2 Principal findings

Diabetes mellitus patients have a significantly higher risk of tooth loss than in non‐DM individuals.

### 6.3 Practical implications

Diabetes mellitus patients shall get attention on oral disease prevention by the dental care practitioners. They are at increased risk of tooth loss, which in particular applies to DM patients from Asia and South America.

## CONFLICT OF INTEREST

The authors declare that they have no conflicts of interest.

This paper was prepared as part of the obligation of the first author to fulfil the requirements of the University of Amsterdam Academic Medical Centre (UvA/AMC) Master's programme in Evidence‐Based Practice in Health Care.

## AUTHOR CONTRIBUTION

L.P.M.W. contributed to design, search and selection, analysis and interpretation and drafted the manuscript. L.Z. contributed to design, analysis and interpretation and critically revised the manuscript. G.A.W. contributed to conception and design, analysis and interpretation and critically revised the manuscript. E.W.P.B. contributed to analysis and interpretation and critically revised the manuscript. D.E.S. contributed to conception and design, search and selection, analysis and interpretation and critically revised the manuscript. *All authors gave final approval and agreed to be accountable for all aspects of work ensuring integrity and accuracy*.

## ETHICAL APPROVAL

Ethical approval was not required. This study is registered at the ACTA University Ethical Committee by number 2021‐71228.

## Supporting information

Supplementary Material

## Data Availability

Data derived from public domain resources. The data that support the findings (the seven included studies) of this study are available from search databases PubMed/Medline or Cochrane‐CENTRAL. These data were derived from resources available in original papers that are published in the public domain. Some first or corresponding authors of inculded papers were contacted for additional data.
