# Supplementary material for: The risk of tooth loss in patients with diabetes: A systematic review and meta‐analysis
Source: Int J Dent Hyg. 2021 Aug 24;20(1):145–66. doi: 10.1111/idh.12512 (PMC9291053; doi:10.1111/idh.12512)
Supplement: Supplementary file 1 — Supplementary Material [file IDH-20-145-s001.pdf]

Online Appendix S1

Excluded studies (N=14) based after the seconds phase of the search and selection procedure. When based on the full-text papers were assessed for the defined eligibility criteria.

Full text not retrievable	1. Ilguy et al. 2007 #<br>2. Fett et al. 1965
No full text in the English language	3. Sampedro Abascal et al. 1996 (Spanish)<br>4. Lopez-Perez et al. 1996 (Spanish)<br>5. Albracht et al. 1991 (Hungarian)
No data available on DM and number of teeth	6. Eklund et al. 1994
Data presented in categories original authors could not provide overall number of teeth	7. Wiener et al. 2017 #<br>8. Jung et al. 2010 #<br>9. Kapp et al. 2007 #
No full-mouth assessment	10. Oliver et al. 1993 #
Number of missing teeth in a period, but not totally in life.	11. Yoo et al. 2019<br>12. Mayard Pons et al. 2015<br>13. Jimenez et al. 2012
The number of people evaluated for DM and non-DM is unclear.	14. Luo et al. 2015 #
Subjects reported number of teeth	15. Hastings et al. 2017<br>16. Similä et al. 2018

The corresponding authors were contacted for clarification by the authors of this review in order to check suitability.

References:

1. Ilgüy M, Ilgüy D, Bayirli G. Dental lesions in adult diabetic patients. New York State Dental Journal. 2007;73(1):58.
2. Fett K, Jutzi E. Dentition of diabetes. Effect of treatment methods on the number of teeth. II. Deutsche zahnärztliche Zeitschrift. 1965;20:902-6.
3. Sampedro CA, Segura JE, Lapetra JP, Llamas RC. Diabetes as a risk factor for tooth loss in the geriatric population. Atencion primaria. 1996;18(4):182-85.
4. López-López J, Jané-Salas E, Estrugo-Devesa A, Velasco-Ortega E, Martín-González J, Segura-Egea JJ. Periapical and endodontic status of type 2 diabetic patients in Catalonia, Spain: a cross-sectional study. Journal of endodontics. 2011;37(5):598-601.

5. Albrecht M, Banoczy J, Dinya E, Tamas Jr. G. Caries status in diabetic patients. Fogorvosi szemle. 1991;84(9):267.
6. Eklund SA, Burt BA. Risk factors for total tooth loss in the United States; longitudinal analysis of national data. Journal of public health dentistry. 1994; 54(1):5-14.
7. Wiener, RC, Shen C, Findley PA, Sambaoorthi U Tan X. The association between diabetes mellitus, sugar-sweetened beverages, and tooth loss in adults: Evidence from 18 states. The Journal of the American Dental Association. 2017;148(7):500-509.
8. Jung SH., Ryu JI, Jung DB. Association of total tooth loss with socio-behavioural health indicators in Korean elderly. Journal of oral rehabilitation. 2011;38(7):517-524.
9. Kapp JM, Boren SA, Yun S, LeMaster J. Peer Reviewed: Diabetes and Tooth Loss in a National Sample of Dentate Adults Reporting Annual Dental Visits. Preventing chronic disease. 2007; 4(3).
10. Oliver RC, Tervonen T. Periodontitis and tooth loss: comparing diabetics with the general population. The Journal of the American Dental Association. 1993;124(12):71-76.
11. Yoo JJ, Kim DW, Kim MY, Kim YT, Yoon JH. The effect of diabetes on tooth loss caused by periodontal disease: A nationwide population-based cohort study in South Korea. Journal of Periodontology. 2019;90(6):576-83.
12. Mayard-Pons ML, Rilliard F, Libersa JC, Musset AM, Farge P. Database analysis of a French type 2 diabetic population shows a specific age pattern of tooth extractions and correlates health care utilization. Journal of Diabetes and its Complications. 2015;29(8): 993-997.
13. Jimenez M, Hu FB, Marino M, Li Y, Joshipura KJ. Type 2 diabetes mellitus and 20 year incidence of periodontitis and tooth loss. Diabetes research and clinical practice. 2011; 98(3):494-500.
14. Luo H, Pan W, Sloan F, Feinglos, M, Wu B. Peer Reviewed: Forty-Year Trends in Tooth Loss Among American Adults With and Without Diabetes Mellitus: An Age-Period-Cohort Analysis. Preventing chronic disease. 2015;12.
15. Hastings JF, Vasquez E. Diabetes and Tooth Loss among Working-Age African Americans: A National Perspective. Social work in public health. 2017;32(7):443-51.
16. Similä T, Auvinen J, Puukka K, Keinänen-Kiukaanniemi S, Virtanen JI. Impaired glucose metabolism is associated with tooth loss in middle-aged adults: The Northern Finland Birth Cohort Study 1966. Diabetes research and clinical practice. 2018;142:110-19.

Online Appendix S2.1

Methodological quality and potential risk of bias scores of the individual included studies at protocol stage

Contains:

Online Appendix S2-2
Preliminary tool for risk of bias in exposure study: Shin et al. 2017
Online Appendix S2-3
Preliminary tool for risk of bias in exposure study: Greenblatt et al. 2016
Online Appendix S2-4
Preliminary tool for risk of bias in exposure study: Costa et al. 2013/2011
Online Appendix S2-5
Preliminary tool for risk of bias in exposure study: Patel et al. 2013
Online Appendix S2-6
Preliminary tool for risk of bias in exposure study: Botero et al. 2012
Online Appendix S2-7
Preliminary tool for risk of bias in exposure study: Sensorn et al. 2012
Online Appendix S2-8
Preliminary tool for risk of bias in exposure study: Kaur et al. 2009
Online Appendix S2-9
Preliminary tool for risk of bias in exposure study: Patiño-Marín et al. 2008
Online Appendix S2-10
Preliminary tool for risk of bias in exposure study: Bacic et al. 1989
Online Appendix S2-11
Preliminary tool for risk of bias in exposure study: Falk et al. 1986

Online Appendix S2.1

Methodological quality and potential risk of bias scores of the individual included studies at protocol stage

Preliminary tool for risk of bias in exposure studies (1): At protocol stage

The aim of the study: The aim of this systematic review is to comprehensively and critically summarize and synthesize the available scientific evidence of observational studies that have evaluated the number of teeth among patients with diabetes mellitus (DM), as compared to individuals without DM (non-DM).

Specify the outcome: Absolute numbers or a population mean of missing teeth, tooth loss as cross sectional data for a subject over lifetime, up to the moment of assessment (not for specific period) based on full-mouth assessment. More tooth loss among DM patients in comparison with the non-DM controls could mean that the presence of diabetes mellitus might harmful.

Specify the research question by defining a generic target experiment:

**Participants**<br>Human subjects ≥ 18 years with and without diabetes mellitus* (undefined, type I and/or type II) and clinically determined number of teeth.<br>P: subjects<br>I: with DM<br>C: without DM<br>O: number of teeth<br>S: observational studies
**Experimental Exposure**<br>Diabetes mellitus
**Control Exposure**<br>No diabetes mellitus

List the confounding domains relevant to all or most studies

Relevant confounding domains are the prognostic factors that predict whether an individual receives one or the other exposure of interest, in this case exposure of DM.

- Demographic factors: age, sex (gender)
- BMI, obesity
- Smoking
- Alcohol intake
- Type of DM (type I/type II; insulin dependent, duration) *
- Severity/control of DM*
- World continent
- Social/economic status (for example; health insurance eligibility status)
- Income level
- General health status, history of medical treatments, medication use (controlling DM)
- Setting: participants selected from the general population, diabetic centres or from specific databases.

**it could also be described as co-exposure.*

List the possible co-exposures that could differ between exposure groups and could have an impact on study outcomes

Relevant co-exposures are the exposures that individuals might receive after or with initiation of the exposure, which are related to the exposure received and which are prognostic for the outcome of interest. The outcome of interest is the number of (missing) teeth.

Additional exposures: co-exposures
Characteristics and exposures that are present at baseline: confounders

- Type of DM (type I/type II; insulin dependent) **
- Severity/control of DM**
- Starting point for calculation (wisdom teeth whether or not included): maximum 28 or 32 teeth.

*** it could also be described as confounders.*

List the criteria used to determine the accuracy of exposure measurement

DM status either self-reported or clinically assessed:

- Clinically assessed: professionally diagnosed diabetes mellitus, based on a reliable source (WHO classification, ICD, ADA guideline, diagnosis code, medication use (prescribed by a doctor)).
- Self-reported: diabetes questionnaire or survey.

Factors to consider when evaluating health outcome assessment

Number of (missing) teeth should be clinically determined.
Factors to consider:

- Reason for tooth loss. For example; tooth loss due to periodontitis, caries or trauma and excluding for surgical extractions and extractions of deciduous teeth.
- Starting point for calculation: third molars in or excluded, based on 28/32 teeth.

Online APPENDIX S2.2-S2.11

Methodological quality and potential risk of bias scores of the individual included studies.

Online Appendix S2-2
Preliminary tool for risk of bias in exposure study: Shin et al. 2017

Title: The number of Teeth is inversely Associated with Metabolic Syndrome: A Korean Nationwide Population-Based study.

Year: 2017

Authors: Hye-Sun Shin (a).

a: Department of Dental Hygiene, Eulji University College of Health Science, Seongnam, Korea.

PMID: **28452621**

AIM: *The purpose of this study was to explore the association between the number of existing permanent teeth and metabolic syndrome (MetS) in a representative sample of the Korean population (by examining socio-demographic factors, oral and general health, and oral and general health-related behaviours).*

Specify a target experiment specific to the study:

☐	The protocol-specified target experiment fully applies	***OR***	Participant	**Participant**<br>From the Korea National Health and Nutrition Examination Survey, a cross- sectional study was conducted on 13,066 participants over the age of 19 years. The participants were randomly selected by geographic area, age and gender based on the 2005 National Census Registry.
			Experimental exposure	**Experimental exposure**<br>Diabetes mellitus (Metabolic Syndrome)
			Control exposure	**Control exposure**<br>Healthy subjects

Specify the outcome

Specify which outcome is being assessed for risk of bias (typically from among those earmarked for the Summary of Findings table). Specify whether this is a proposed benefit or harm of exposure.

Assessment of the number of existing permanent teeth (after the use of exclusion criteria: missing teeth, impacted or implants and excluding wisdom teeth). The number of existing permanent teeth was divided into categories. Therefore, we request data to receive the prevalence in percentage of absolute numbers of teeth among diabetes mellitus patients and non-diabetes controls.

Is your aim for this study…?

- ■ to assess the effect of initiating intervention (as in an intention-to-treat analysis)
- ☐ to assess the effect of initiating and adhering to intervention (as in a per-protocol analysis)
- ☐ other (specify)

Specify the numerical result being assessed

In case of multiple alternative analyses being presented, specify the numeric result (e.g. RR = 1.52 (95% CI 0.83 to 2.77) and/or a reference (e.g. to a table, figure or paragraph) that uniquely defines the result being assessed.

Statistical analyses were performed according to the KNHANES guidelines for application of the complex survey design and sampling weights. In the analysis, the dependent variable was MetS and the independent variable was the number of teeth. The characteristics of the participants and the number of teeth were presented with frequency distributions for categorical variables using the chi-square test. They then presented the distribution of MetS and its components according to the number of teeth using the chi-square test. All data were presented as weighted percentage and standard error.<br><br>They calculated the means and standard errors for metabolic components using analysis of variance (ANOVA) and analysis of covariance (ANCOVA). Multivariable logistic regression was applied to evaluate the association between the number of teeth after controlling for confounders. The crude and adjusted odds ratio (AOR) and confidence intervals (CIs) as well as the p-values for each logistic model

were calculated.

- Model 1 consisted of a crude association.
- Model 2 was adjusted for demographic and socio-economic variables including age, gender, income, and education.
- Model 3 was adjusted for demographic and socio-economic variables, with oral health status and behaviours including tooth-brushing frequency and periodontitis.
- Model 4 was adjusted for Model 3 with general health status and behaviour variables including smoking, drinking, physical activity, and diabetes mellitus.
- Model 5 was adjusted for all the aforementioned variables and five metabolic syndromes accept itself in order to reveal the association the number of teeth and MetS components.

Finally, subgroup analyses were performed to identify specific risk groups. Statistical significance was determined at P<0.05.

Preliminary consideration of confounders

Complete a row for each important confounding area (i) listed in the review protocol; and (ii) relevant to the setting of this particular study, or which the study authors identified as potentially important. *"Important" confounding areas are those for which, in the context of this study, adjustment is expected to lead to a clinically important change in the estimated effect of the exposure. "Validity" refers to whether the confounding variable or variables fully measure the area, while "reliability" refers to the precision of the measurement (more measurement error means less reliability).*

**(i) Confounding areas listed in the review protocol**				
Confounding area	Measured variable(s)	Is there evidence that controlling for this variable was unnecessary? *	Is the confounding area measured validly and reliably by this variable (or these variables)?	OPTIONAL: Is adjusting for this variable (alone) expected to move the effect estimate up or down?

Demographic factors	Age, gender	No evidence that controlling was unnecessary.	Yes.	
Social/economic status	Income	No evidence that controlling was unnecessary.	Yes: income was measured as the household income and was categorized into quartiles.	
General health status and behaviours	Smoking, drinking	No evidence that controlling was unnecessary.	Yes: based on the response to questions about current smoking status, the participants were divided into two groups<br>- No: those who had never smoked before and past smokers,<br>- Yes: those who were current smokers<br><br>Alcohol consumption was categorized into five groups:<br>- non-drinker<br>- almost non-drinker (≤1 day per month)<br>- light drinker (2-4 days per month)<br>- moderate drinker (2-3 days per week)<br>- heavy drinker (≥4 days per week)<br><br>Drinking was dichotomized into two groups: no (none/almost non-drinker) and yes (light/moderate/heavy drinker).	

(ii) **Additional confounding areas relevant to the setting of this particular study, or which the study authors identified as important**

Confounding area	Measured variable(s)	Is there evidence that controlling for this variable was unnecessary? *	Is the confounding area measured validly and reliably by this variable (or these variables)?	OPTIONAL: Is adjusting for this variable (alone) expected to move the effect estimate up or down?
Social/economic status	Education	No evidence that controlling was unnecessary.	Yes: Education level was categorized into four groups: below primary school, middle school, high school, and college or higher.	
Oral health status and behaviours	Tooth brushing, frequency and periodontitis	No evidence that controlling was unnecessary.	Yes.<br><br>The daily tooth-brushing frequency was categorized into two groups:<br><br>- Less than three times a day and three<br>- More times a day.<br><br>Periodontal status and state of dentition were carefully assessed by dentists, and the Community Periodontal Index of Treatment Needs (CPITN) was used to measure periodontitis.<br><br>The selected teeth were numbers 11, 16, 17, 26, 27, 31, 36, 37, 46, and 47, according to the World Health Organization guidelines.<br><br>CPI was rated on a scale of 0 to 4:<br><br>- 0 (normal),<br>- 1 (gingivitis with bleeding	

			on probing),<br>- 2 (presence of calculus),<br>- 3 (pocket depth≥3.5mm), and 4 (pocket depth≥5.5mm).<br><br>Periodontal status was grouped into two categories:<br><br>- absence (CPI 1-2) periodontitis<br>- presence (CPI 3-4) periodontitis.	
General health status behaviours	Physical activity, diabetes mellitus	No evidence that controlling was unnecessary.	Yes.<br><br>Physical activity level was assessed as having walked for at least 10 minutes during the last week. Physical activity was dichotomized into two groups:<br><br>- less than three days a week<br>- four or more times a week<br><br>Diabetes mellitus was defined as having a fasting glucose level over 126 mg/dL or being medicated for diabetes.	

* In the context of a particular study, variables can be demonstrated not to be confounders and so not included in the analysis: (a) if they are not predictive of the outcome; (b) if they are not predictive of exposure; or (c) because adjustment makes no or minimal difference to the estimated effect of the primary parameter. Note that “no statistically significant association” is not the same as “not predictive”.

Preliminary consideration of criteria used to determine the accuracy of measurement of exposure and outcome

Complete a row for each measure listed in the study for the (i) exposure and (ii) outcome. Of the measures listed in the protocol, consider the sensitivity, specificity, and confidence in the methods used in the study.

(i) Exposure measurement method listed in the study		
Method of measurement	Measured exposure	Is the exposure measured validly and reliably by this method (or these methods)?
Interviews using structured questionnaires	Diabetes Mellitus	Yes: defined as having a fasting glucose level over 126 mg/dL of being medicated for diabetes.
The presence of at least three of the following components:<br>1. waist circumference<br>2. elevated triglycerides<br>3. reduced high-density lipoprotein<br>4. elevated blood pressure<br>5. elevated fasting glucose	Metabolic Syndrome	Yes: following the suggestion of the Joint Interim Statement of the International Diabetes Federation Task Force on Epidemiology and Prevention*.<br><br>**Alberti KG, Eckel RH, Grundy SM, et al. Harmonizing the metabolic syndrome: a joint interim statement of the International Diabetes Federation Task Force on Epidemiology and Prevention; National Heart, Lung, and Blood Institute; American Heart Association; World Heart Federation; International Atherosclerosis Society; and International Association for the Study of Obesity. Circulation 2009;120:1640-1645.*

(ii) Outcome measurement method listed in the study		
Method of measurement	Measured outcome	Is the outcome measured validly and reliably by this method (or these methods)?
Intra-oral examination	Number of existing permanent teeth	Yes, intra-oral examination performed by trained dentists. the number of teeth was obtained after the use of exclusion criteria: missing teeth, impacted or implants and excluding wisdom teeth.

		The number of teeth was divided into three categories:<br>- 0-19 teeth -> 20 teeth has been proposed as cut-off for severe tooth loss*<br>- 20-27 teeth<br>- 28 teeth<br><br>* Han DH, Khang YH, Lee HJ. Association between adult height and tooth loss in a representative sample of Koreans. *Community Dent Oral Epidemiol* 2015;43:479-488.<br><br>We request data to receive the prevalence in percentage of absolute numbers of teeth among diabetes mellitus patients and non-diabetes controls by emailing the corresponding authors. We liked to know the average number of teeth spread out of the full population and not presented in categories.<br><br>Data request:<br><br>The weighted mean number of teeth According to the Diabetes mellitus<br><br>Diabetes mellitus / the weighted mean number of teeth<br>Total: 25.29<br>No: 25.57<br>Yes: 22.32

Preliminary consideration of co-exposures

Complete a row for each important co-intervention (i) listed in the review protocol; and (ii) relevant to the setting of this particular study, or which the study authors identified as important.
"Important" co-interventions are those for which, in the context of this study, adjustment is expected to lead to a clinically important change in the estimated effect of the intervention.

**(i)**	**Co-exposures listed in the review protocol**	
Co-exposure	Is there evidence that controlling for this co-exposure was unnecessary (e.g., because it was not administered)?	Is presence of this co-exposure likely to favor outcomes in the experimental or the control group
		Favor experimental / Favor comparator / No information
		Favor experimental / Favor comparator / No information
		Favor experimental / Favor comparator / No information

**(ii)**	**Additional co-exposures relevant to the setting of this particular study, or which the study authors identified as important**	
Co-exposure	Is there evidence that controlling for this co-exposure was unnecessary (e.g., because it was not administered)?	Is presence of this co-exposure likely to favor outcomes in the experimental or the control group
		Favor experimental / Favor comparator / No information
		Favor experimental / Favor comparator / No information
		Favor experimental / Favor comparator / No information

Risk of bias assessment (cohort-type studies)

Bias due to confounding	1.1 **Is there potential for confounding of the effect of exposure in this study? If N or PN to 1.1:** the study can be considered to be at low risk of bias due to confounding and no further signalling questions need be considered	NA / **Y** / PY / PN / N / NI	Selected potential confounders included demographic and socio-economic factors, oral health status and behaviours, and general health and behaviours.
	**If Y/PY to 1.1, answer 1.2 and 1.3 to determine whether there is a need to assess time-varying confounding:**		
	**1.2. If Y or PY to 1.1: Was the analysis based on splitting follow up time according to exposure received?**<br>**If N or PN to 1.2**, answer questions 1.4 to 1.6, which relate to baseline confounding	NA / Y / PY / PN / **N** / NI	The participants could not switch between exposures, so the outcome could not be biased due time varying confounding.
	**1.3. If Y or PY to 1.2: Were exposure discontinuations or switches likely to be related to factors that are prognostic for the outcome?**	NA / **Y** / PY / PN / N / NI	Yes, it is possible that there will be undiagnosed diabetics in the non-diabetic (control) group due of misclassification.
	**If N or PN to 1.3**, answer questions 1.4 to 1.6, which relate to baseline confounding		
	**1.4. Did the authors use an appropriate analysis method that adjusted for all the critically important confounding areas?**	NA / **Y** / PY / PN / N / NI	Multivariable logistic regression was applied to evaluate the association between the number of teeth after controlling for confounders.
	**1.5. If Y or PY to 1.4: Were confounding areas that were adjusted for measured validly and reliably by the variables available in this study?**	NA / **Y** / PY / PN / N / NI	Yes.<br><br>Previous studies showed that the potential confounders possibly tended to bias the outcome.
	**1.6. Did the authors avoid adjusting for post-exposure variables?**	**NA** / Y / PY / PN / N / NI	No information about post-exposure variables.
	**If Y or PY to 1.3**, answer questions 1.7 and 1.8, which relate to time-varying confounding		

	**1.7. Did the authors use an appropriate analysis method that adjusted for all the critically important confounding areas and for time-varying confounding?**	NA / Y / PY / PN / N / NI	Due the cross-sectional design: the results have been measured at one point at the time, there is no information about time varying.
	**1.8. If Y or PY to 1.7: Were confounding areas that were adjusted for measured validly and reliably by the variables available in this study?**	NA / **Y** / PY / PN / N / NI	Previous studies showed that the potential confounders possibly tended to bias the outcome.
	**Risk of bias judgement**	**Moderate**	**Appropriate adjustment for potential confounders and multivariable logistic regression was applied to evaluate the association between the number of teeth after controlling for confounders.**
Bias in selection of participants into the study	**2.1. Was selection of participants into the study (or into the analysis) based on variables measured after the start of the exposure?**<br><br>**If N or PN to 2.1 go to 2.4**	Y / PY / PN / **N** / NI	No, participants were drawn from the KNHANES, a study periodically conducted by the Korea Centre for Disease Control and Prevention in 2012-2014.
	**2.2. If Y/PY to 2.1: Were the post-exposure variables that influenced selection associated with exposure?**	Y / PY / PN / **N** / NI	The sampling protocol was designed to include a complex, stratified, multistage, and probability–cluster survey of a representative sample of the non-institutionalized civilian population. The participants were randomly selected by geographic area, age, and gender based on the 2005 National Census Registry.
	**2.3. If Y/PY to 2.2: Were the post-exposure variables that influenced eligibility selection influenced by the outcome or a cause of the outcome?**	NA/ Y/ PY / PN / N / **NI**	Not described.
	**2.4 Do start of follow-up and start of exposure coincide for most participants?**	NA/ Y/ PY / PN / **N** / NI	No, the start of exposure already exists prior of the study, but it is possible that severity of Mets/DM changes between the participants.

	**2.5 If Y/PY to 2.2 and 2.3, or N/PN to 2.4: Were adjustment techniques used that are likely to correct for the presence of selection biases?**	NA / Y / PY / PN / N / NI	No, not neccesary. The authors used a representive sample.
	**Risk of bias judgement**	**Low**	**The KNHANES database used for the study is a large sample size of a representative Korean population. The authors analysed the entire Korean sample, including young adults and elders.**
Bias in classification of exposures	**3.1 Is exposure status well defined?**	**Y** / PY / PN / N / NI	Yes, METS is clearly defined. The authors made a subhead for the assessment of Mets and definition.
	**3.2 Did entry into the study begin with start of the exposure?**	**Y** / PY / PN / N / NI	Yes, because of the cross-sectional design. No detailed information about the reason and timing tot tooth loss.<br>It is possible that there will be undiagnosed diabetics in the non-diabetic (control) group.
	**3.3 Was information used to define exposure status recorded prior to outcome assessment?**	Y / PY / PN / N / **NI**	No information.
	**3.4 Could classification of exposure status have been affected by knowledge of the outcome or risk of the outcome?**	Y / PY / PN / **N** / NI	The trained dentists conducted the intra-oral examination for the assessment of the number of teeth and physicians performed the metabolic syndrome assessment.
	**3.5 Were exposure assessment methods robust (including methods used to input data)?**	Y **/** PY / PN / N / **NI**	No information.
	**Risk of bias judgement**	**Low**	**Anthropometry assessment were perfomed clinically by physicans and they were unknown for outcome assessment.**
	Optional: What is the predicted direction of bias due to measurement of outcomes or exposures?	Favours experimental	It is possible that there will be undiagnosed diabetics in the non-diabetic (control) group which leads to the predicted direction of bias

			favours experimental, because of the underestimation in the DM group.
Bias due to departures from intended exposures	**4.1. Is there concern that changes in exposure status occurred among participants?**<br>**If your aim for this study is to assess the effect of initiating and adhering to an exposure (as in a per-protocol analysis), answer questions 4.2 and 4.3, otherwise continue to 4.4 if Y or PY to 4.1.**	**Y** / PY / PN / N / NI	Yes, because the cross-sectional design.
	**4.2. Did many participants switch to other exposures?**	Y / PY / PN / N / **NI**	It is possible that severity of diabetes and/or MetS changes over time.
	**4.3. Were the critical co-exposures balanced across exposure groups?**	Y / PY / PN / N / NI	Yes, the authors used a complex, stratified, multistage, and probability–cluster survey of a representative sample of the non-institutionalized civilian population
	4.4. **If NY/PN PY to 4.1, or Y/PY to 4.2, or 4.3:** Were adjustment techniques used that are likely to correct for these issues?	NA / Y / PY / PN / **N** / NI	Not necessary.
	**Risk of bias judgement**	**Moderate**	**There were deviations from usual practice, but their impact on the outcome is expected to be slight. The authors mask these limitations by using the cross-sectional design.**
Bias due to missing data	**5.1 Were there missing outcome data?**	Y / PY / PN / N / **NI**	Missing data prior at the start of the study. Missing outcome data during or after analysis were not reported.
	**5.2 Were participants excluded due to missing data on exposure status?**	**Y** / PY / PN / N / NI	Only participants who had no missed main information such as oral examination, health status variables and laboratory test for Mets were included.
	**5.3 Were participants excluded due to missing data on other variables needed for the analysis?**	**Y** / PY / PN / N / NI	Yes, see 5.2

	**5.4 If Y/PY to 5.1, 5.2 or 5.3: Are the proportion of participants and reasons for missing data similar across exposures?**	NA / **Y** / PY / PN / N / NI	No not necessary, because the missing data already exist before start of the study. Participants with these specific missing data were excluded, so only participants with complete data participated.
	**5.5 If Y/PY to 5.1, 5.2 or 5.3: Were appropriate statistical methods used to account for missing data?**	NA / Y / PY / PN / **N** / NI	See above 5.4
	**Risk of bias judgement**	**Low**	**Data is complete.**
Bias in measurement of outcomes	**6.1 Could the outcome measure have been influenced by knowledge of the exposure received?**	Y / PY / PN / N / **NI**	Unclear if the trained dentists who performed assessment of the number of teeth were aware of the aim of the study.<br><br>It is not likely that knowledge of DM/MetS status influenced the assessment of number of existing teeth.
	**6.2 Was the outcome measure sensitive?**	Y / PY / PN / N / NI	The outcome measurement is a surrogate/hard endpoint and not sensitive (for change).
	**6.3 Were outcome assessors unaware of the exposure received by study participants?**	Y / PY / PN / N / **NI**	No information, but not likely to happen.
	**6.4 Were the methods of outcome assessment comparable across exposure groups?**	**Y** / PY / PN / N / NI	Yes, all the participants underwent an intra-oral examination and so recorded their oral health status.
	**6.5 Were any systematic errors in measurement of the outcome unrelated to exposure received?**	Y / PY / PN / N / NI	It is not likely that assessment of the number of existing teeth bring any systematic errors in measurment, because tooth loss is a surrogate endpoint en not sensitvie for change, see 6.2.

			Thereby the authors reported that impacted, implants and wisdom teeth were excluded for further anaylis.
	**Risk of bias judgement**	**Low**	**The methods of outcome assessment were comparable across exposure groups.**
Bias in selection of	Is the reported effect estimate likely to be selected, on the basis of the results, from...?		
the reported result	**7.1. ... multiple outcome *measurements* within the outcome domain?**	**Y** / PY / PN / N / NI	Yes, only the number of teeth is assessed and reported.
	**7.2 ... multiple *analyses* of the exposure-outcome relationship?**	**Y** / PY / PN / N / NI	Yes, several models (for adjustment) are created and reported in the study (Table 4). After, the authors adjusted for age and gender stratified association in table 5.
	**7.3 ... different *subgroups*?**	**Y** / PY / PN / N / NI	Analysis were performed in different categories for the number of teeth. Second, subsequent subgroup analyses were performed to identify specific groups according to the association between the number of teeth and MetS.
	**Risk of bias judgement**	**Low**	**All data were fully described.**
Overall bias	**Risk of bias judgement**	**Low**	**The sample size that is used is a large representative Korean population and could be generalised to all age groups, young adults and elders were included for analysis. Subgroups were performed to identify specific groups according the association. Third, the study adjusted for potential confounders in 5 different models.**

Online Appendix S2-3

Preliminary tool for risk of bias in exposure study: Greenblatt et al. 2016

Title: Association of diabetes with tooth loss in Hispanic/Latino adults: findings from the Hispanic Community Health Study/Study of Latinos

Year: 2016

Authors: Ariel P Greenblatt, Christian R Salazar, Mary E Northridge, Robert C Kaplan, George W Taylor, Tracy L Finlayson, Qibin Qi, Victor Badner.

PMID: 27239319

AIM*: investigate the association between diabetes mellitus and missing teeth in Hispanic/Latino adults from diverse heritage groups who reside in the USA and examine how diabetes is related to cumulative tooth loss in Hispanic populations across age group and by gender.*

Specify a target experiment specific to the study:

Community based cohort study of Hispanic/Latino adults in the US.

☐ The protocol-specified target experiment fully applies	*OR*	Participant<br><br>Experimental exposure<br><br>Control exposure	**Participant**<br>rom the Hispanic Community Health Study/Study of Latinos = HCHS/SOL community based prospective cohort study of 16415 self-identified Hispanic/Latino persons aged between 18-74 at screening from randomly selected households in four US field centers. The analytic sample comprised 15132 dentate and 833 dentate participants with complete data regarding missing teeth and diabetes.<br>**Experimental outcome**<br>Diabetes: HbA1c ≥ 6.5% or fasting time >8h and fasting glucose ≥ 126 mg/dL or fasting time <8h and fasting glucose ≥ 200 mg/dL, N= 2792.<br>- Controlled, N=1468<br>- Uncontrolled, N= 1324<br>Impaired glucose tolerance: HbA1c of 5.7–6.4% or fasting time >8 h and fasting glucose of 100–125 mg/ dL.<br>- Prediabetes, N= 5842<br>**Control exposure**<br>Normal glucose tolerance HbA1c <5.7% and fasting glucose <100 mg/dL, N= 6467

Specify the outcome

Specify which outcome is being assessed for risk of bias (typically from among those earmarked for the Summary of Findings table). Specify whether this is a proposed benefit or harm of exposure.

Trained and calibrated dental examiners (n=13 across all four study sites) performed a comprehensive oral examination for dental caries and periodontal disease using full-mouth assessments. The examiners determined tooth status by means of visual examination of all teeth present except third molars, for a total count of 28 teeth. If a tooth was missing, the examiner made a determination, after discussion with the participant, of
the reason for the tooth's absence (trauma, periodontal disease, caries or orthodontic treatment).
Only counted teeth that were determined missing due to periodontal diseases or caries are presented in the analyses.

The specific outcomes for missing teeth used in the analysis were tooth loss ≥9 teeth and total edentulism (missing 28 teeth, not including wisdom teeth). We defined impaired oral function as missing 9 or more teeth, because adequate oral functioning has been defined as having 20 or more teeth, which equates to missing 8 or fewer teeth (not including third molars).

Is your aim for this study…?

■ to assess the effect of initiating intervention (as in an intention-to-treat analysis)

☐ to assess the effect of initiating and adhering to intervention (as in a per-protocol analysis)

☐ other (specify)

Specify the numerical result being assessed

In case of multiple alternative analyses being presented, specify the numeric result (e.g. RR = 1.52 (95% CI 0.83 to 2.77) and/or a reference (e.g. to a table, figure or paragraph) that uniquely defines the result being assessed.

In descriptive analyses:

- Mean numbers of missing teeth in relation to participant characteristics for dentate participants using predicted marginal means and 95% CIs based on Taylor series linearization from log-linear models.

Associations between diabetes and tooth loss:

- ORs and 95% CI of having nine or more missing teeth in dentate participants using logistic regression models adjusting for age, sex, Hispanic background group, study site, nativity, status, income, education, last dental visit, current health insurance status, alternative healthy eating index, cigarette smoking, obesity, chronic periodontitis, CRP levels, and percent decay and filled teeth.

Edentulous in relation to diabetes status:

- Estimation ORs and 95% CI
- Exploratory analyses; stratified models by Hispanic background group to evaluate whether associations between diabetes and missing teeth differed across Hispanic backgrounds.

Statistical interaction of diabetes with missing teeth

- By sex and age group (18–44,45–65, 65+) in separate logistic regression models using interaction terms (ie, diabetes×sex; diabetes×age group) and adjusted for age, Hispanic background group, study site, nativity status, income, education, number of dental visits, current health insurance status, alternative healthy eating index, cigarette smoking, and obesity.

All tests were two-sided and statistical significance was defined as $p<0.05$.

Preliminary consideration of confounders

Complete a row for each important confounding area (i) listed in the review protocol; and (ii) relevant to the setting of this particular study, or which the study authors identified as potentially important. *"Important" confounding areas are those for which, in the context of this study, adjustment is expected to lead to a clinically important change in the estimated effect of the exposure. "Validity" refers to whether the confounding variable or variables fully measure the area, while "reliability" refers to the precision of the measurement (more measurement error means less reliability).*

**(iii) Confounding areas listed in the review protocol**				
Confounding area	Measured variable(s)	Is there evidence that controlling for this variable was unnecessary? *	Is the confounding area measured validly and reliably by this variable (or these variables)?	OPTIONAL: Is adjusting for this variable (alone) expected to move the effect estimate up or down?
Demographic characteristics	Age, gender		Yes / No / No information	Favor intervention / Favor control / No information
Social/economic factors	Household income, health insurance			
Behavioural covariates	Smoking level, BMI			

(iv) **Additional confounding areas relevant to the setting of this particular study, or which the study authors identified as important**				
Confounding area	Measured variable(s)	Is there evidence that controlling for this variable was unnecessary? *	Is the confounding area measured validly and reliably by this variable (or these variables)?	OPTIONAL: Is adjusting for this variable (alone) expected to move the effect estimate up or down?
Demographic characteristics	Nativity status, Hispanic background		Yes / No / No information	Favor intervention / Favor control / No information
Behavioural covariates	Physical activity level, healthy eating		Yes, according to WHO Global Physical Activity Questionnaire* or combination of activity recommend by the US physical activity guideline for adults. And healthy eating using the Alternative Healthy eating index 2010 (HEI-2010)***<br><br>** Bull FC, Maslin TS, Armstrong T, et al. Global physical activity questionnaire (GPAQ): nine country reliability and validity study. J Phys Act Health 2009; 6:790–804.*<br><br>*** U.S. Department of Health and Human Services. 2008 Physical activity guidelines for Americans. Washington DC, 2008. http:// health.gov/guidelines/guidelines*<br>**** Guenther PM, Casavale KO, Reedy J, et al. Update of the Healthy Eating Index: HEI-2010. J Acad Nutr Diet 2013;113:569–80.*	
Social/economic factors	Education level, field centre			
Clinical characteristics	DMFT-index, periodontal diseases		*DMFT index, periodontal disease classification**<br><br>**Schuller AA, Holst D. Oral status indicators DMFT and FS-T: reflections on index selection. Eur J Oral Sci 2001;109: 155–9.*	Results: adjustment for decayed and filled teeth attenuated all observed associations by an appreciable amount in the overall population → favour control.

			***Eke PI, Page RC, Wei L, et al. Update of the case definitions for population-based surveillance of periodontitis. J Periodontol 2012;83:1449–54.*	
	Dental visit		<1, 1-4 and >4 years ago*<br><br>**Beck JD, Youngblood M Jr, Atkinson JC, et al. The prevalence of caries and tooth loss among participants in the Hispanic Community Health Study/Study of Latinos. J Am Dent Assoc 2014;145:531–40.*	

* In the context of a particular study, variables can be demonstrated not to be confounders and so not included in the analysis: (a) if they are not predictive of the outcome; (b) if they are not predictive of exposure; or (c) because adjustment makes no or minimal difference to the estimated effect of the primary parameter. Note that "no statistically significant association" is not the same as "not predictive".

Preliminary consideration of criteria used to determine the accuracy of measurement of exposure and outcome

Complete a row for each measure listed in the study for the (i) exposure and (ii) outcome. Of the measures listed in the protocol, consider the sensitivity, specificity, and confidence in the methods used in the study.

(iii) Exposure measurement method listed in the study		
Method of measurement	Measured exposure	Is the exposure measured validly and reliably by this method (or these methods)?
ASA guidelines	Diabetes (glycemic status)	Yes. According to the ASA guidelines* using fasting blood glucose levels and HbA1c percentages and antidiabetic medication:<br>- Diabetes<br>- Impaired glucose tolerance<br>- Normal glucose tolerance<br><br>**American Diabetes Association. Classification and diagnosis of diabetes. Sec 2. In: standard of medical care in diabetes – 2016. Diabetes care 2016; 39(1):S13-22.*
ASA guidelines	Controlled/ uncontrolled diabetes	Yes, according to the ASA guidelines.<br>HbA1c<7% to indicate controlled diabetes and HbA1c>7% to indicate uncontrolled diabetes*.

		**American Diabetes Association. Classification and diagnosis of diabetes. Sec 2. In: standard of medical care in diabetes – 2016. Diabetes care 2016; 39(1):S13-22.*

(iv) Outcome measurement method listed in the study		
Method of measurement	Measured outcome	Is the outcome measured validly and reliably by this method (or these methods)?
Oral (visual) examination	Missing teeth due caries or periodontal diseases (except third molars)	Yes, only teeth that are missing due to caries or periodontal diseases were included in the study. The examination determined (after discussion with the participant) the reason for tooth absence; trauma, periodontal diseases, caries, orthodontic treatment or other reasons.
Oral (visual) examination	Impaired oral function	Yes.<br>Missing 9 or more teeth*; because adequate oral functioning has been defined as having 20 or more teeth; which equates to missing 8 or fewer teeth. Wisdom teeth are not included.<br><br>**Godfredsen K, Walls AW. What dentition assures oral function? Clinical Oral Implants Res 2007; 18(3):34-45*.

Preliminary consideration of co-exposures

Complete a row for each important co-intervention (i) listed in the review protocol; and (ii) relevant to the setting of this particular study, or which the study authors identified as important.
"Important" co-interventions are those for which, in the context of this study, adjustment is expected to lead to a clinically important change in the estimated effect of the intervention.

**(iii) Co-exposures listed in the review protocol**		
Co-exposure	Is there evidence that controlling for this co-exposure was unnecessary (e.g., because it was not administered)?	Is presence of this co-exposure likely to favor outcomes in the experimental or the control group
		Favor experimental / Favor comparator / No information

		Favor experimental / Favor comparator / No information
		Favor experimental / Favor comparator / No information

**(iv)**	**Additional co-exposures relevant to the setting of this particular study, or which the study authors identified as important**	
Co-exposure	Is there evidence that controlling for this co-exposure was unnecessary (e.g., because it was not administered)?	Is presence of this co-exposure likely to favor outcomes in the experimental or the control group
		Favor experimental / Favor comparator / No information
		Favor experimental / Favor comparator / No information
		Favor experimental / Favor comparator / No information

Results:

- Tooth loss by patient characteristics (table 1)
- Tooth loss and diabetes status (table 2)
  - Overall study population
  - By gender

Risk of bias assessment (cohort-type studies)

Bias due to confounding	1.1 **Is there potential for confounding of the effect of exposure in this study?**<br><br>**If N or PN to 1.1:** the study can be considered to be at low risk of bias due to confounding and no further signalling questions need be considered	**Y** / PY / PN / N	Cross-sectional design. Whether diabetes preceded the tooth loss, or vice versa, cannot be determined. Potential confounders could play a role between this pathway.
	**If Y/PY to 1.1, answer 2.1 and 1.3 to determine whether there is a need to assess time-varying confounding:**		
	**1.2. If Y or PY to 1.1: Was the analysis based on splitting, follow up time according to exposure received?**<br><br>**If N or PN to 1.2**, answer questions 1.4 to 1.6, which relate to baseline confounding	NA / Y / PY / PN / **N** / NI	The participants could not switch between exposures, so the outcome could not be biased due time varying confounding. On the other hand, there could be difference is exposure status at the start of the study and later on; see 1.3.
	**1.3. If Y or PY to 1.2: Were exposure discontinuations or switches likely to be related to factors that are prognostic for the outcome?**	NA / Y / PY / PN / N / **NI**	It is possible that there will be undiagnosed diabetics in the non-diabetic (control) group due of misclassification, there is no information given about that.
	**If N or PN to 1.3**, answer questions 1.4 to 1.6, which relate to baseline confounding		
	**1.4. Did the authors use an appropriate analysis method that adjusted for all the critically important confounding areas?**	NA / **Y** / PY / PN / N / NI	Adjustment for several confounders.
	**1.5. If Y or PY to 1.4: Were confounding areas that were adjusted for measured validly and reliably by the variables available in this study?**	NA / **Y** / PY / PN / N / NI	Yes, thereby scaling covariates are based on reliable sources. For example:<br>- Adjustment for decayed and filled teeth, using DMFT index.

			- Physical activity using an WHO Global Physical Activity questionnaire.
	**1.6. Did the authors avoid adjusting for post-exposure variables?**	NA / Y / PY / PN / N / **NI**	No information about post exposure variables.
	**If Y or PY to 1.3**, answer questions 1.7 and 1.8, which relate to time-varying confounding		

	**1.7. Did the authors use an appropriate analysis method that adjusted for all the critically important confounding areas and for time-varying confounding?**	NA / **Y** / PY / PN / N / NI	Yes, described in statistical analysis. The authors used logistic regression to test statistical interaction.
	**1.8. If Y or PY to 1.7: Were confounding areas that were adjusted for measured validly and reliably by the variables available in this study?**	NA / **Y** / PY / PN / N / NI	Yes, thereby scaling covariates are based on reliable sources. For example:<br>- Adjustment for decayed and filled teeth, using DMFT index.<br>- Physical activity using an WHO Global Physical Activity questionnaire.
	**Risk of bias judgement**	**Low**	**Confounding expected, all known critically important confounding domains appropriately measured and adjusted (reliably and validity).**
	Optional: What is the predicted direction of bias due to confounding?	**Favours experimental**	It is possible that there will be undiagnosed diabetics in the non-diabetic control. Possibly resulting in more tooth loss in the no-DM group favours experimental.
Bias in selection of participants	2.1. Was selection of participants into the study (or into the analysis) based on variables measured after the start of the exposure?	Y / PY / PN / N / **NI**	Detailed information about the sampling design is given in a study elsewhere:

into the study	**<u>If N or PN to 2.1 go to 2.4</u>**		*Sorlie PD, Aviles-Santa LM, Wassertheil-Smoller S, et al. Designand implementation of the Hispanic Community Health Study/Study of Latinos. Ann Epidemiol 2010; 20:629–41.*<br>- Community description and detailed information about involvement<br>- Participants sampling and recruitment<br><br>The exposure status was existed at the beginning of the study. There is no information given over possible intermediate measurements about DM status. It is not expected that selection of the participants is based on variables measures after the start of the exposure.
	**2.2. <u>If Y/PY to 2.1:</u> Were the post-exposure variables that influenced selection associated with exposure?**	**Y** / PY / PN / N / NI	Yes, it is well known that severity of diabetes possibly changes over time but is not possible to determine this variation due the cross-sectional design.
	**2.3. <u>If Y/PY to 2.2:</u> Were the post-exposure variables that influenced eligibility selection influenced by the outcome or a cause of the outcome?**	NA / Y / PY / **PN** / N / NI	Selection bias is limited. See description *Sorlie et al. 2010*. Woman and men who self-identify as Hispanic or Latino, age 18-72, from a random sample of households in defined communities in the Bronx, Chicago, Miami and San Diego. The participants not differ from the population of interest.
	**2.4 Do start of follow-up and start of exposure coincide for most participants?**	NA / Y / PY / PN / N / **NI**	There is no information given about the start of follow up.
	**2.5 If Y/PY to 2.2 and 2.3, or N/PN to 2.4: Were adjustment techniques used that are likely to correct for the presence of selection biases?**	NA / Y / PY / PN / **N** / NI	Selection bias is limited.

	**Risk of bias judgement**	**Low**	**All participants who would have been eligible for the target trial were included in the study and start of follow up. Data used from the HCHS/SOL community based prospective cohort, this is a big and representative sample.**
	Optional: What is the predicted direction of bias due to selection of participants into the study?	**Towards null**	Limited selection bias.
Bias in classification of exposures	**3.1 Is exposure status well defined?**	**Y** / PY / PN / N / NI	A detailed description about the exposure status is available under the chapter method, glycemic status, and based on a reliable source: ADA guideline.
	**3.2 Did entry into the study begin with start of the exposure?**	Y / **PY** / PN / N / NI	A group with DM and controls with no DM. Undiagnosed diabetics might be classified as a non-diabetic group.
	**3.3 Was information used to define exposure status recorded prior to outcome assessment?**	Y / PY / PN / N / NI	Yes, information about exposure status is used prior to outcome assessment. No information is given if the purpose of these particular study was evident among the study population.
	**3.4 Could classification of exposure status have been affected by knowledge of the outcome or risk of the outcome?**	Y / PY / PN / N / NI	No, a tooth is missing or not. It could not be affected by knowledge of the outcome.
	**3.5 Were exposure assessment methods robust (including methods used to input data)?**	**Y** / PY / PN / N / NI	Using the ADA guidelines to categorize participants. These data already exist.
	**Risk of bias judgement**	**Low**	**The only limitation is that diabetes is measured retrospectively. Exposure status is well defined and based on a reliable source.**

Bias due to departures from intended exposures	**4.1. Is there concern that changes in exposure status occurred among participants?**<br><br>**If your aim for this study is to assess the effect of initiating and adhering to an exposure (as in a per-protocol analysis), answer questions 4.2 and 4.3, otherwise continue to 4.4 if Y or PY to 4.1.**	**Y** / PY / PN / N / NI	Yes.<br><br>Undiagnosed diabetics might be classified as a non-diabetic group, because the exposure status is diagnosed previously. There is a distinction made in severity.<br><br>Unable to distinguish between type I and type II diabetes mellitus in this data set; unable to analyse the data based on type of diabetes only based on uncontrolled/controlled.
	**4.2. Did many participants switch to other exposures?**	Y / PY / PN / N / **NI**	Undiagnosed diabetics might be classified as a non-diabetic group.
	**4.3. Were the critical co-exposures balanced across exposure groups?**	Y / PY / PN / N / **NI**	Only a detailed description is made from the whole cohort in table 1, not specifically for the DM/no-DM group.
	4.4. **If NY/PN PY to 4.1, or Y/PY to 4.2, or 4.3:** Were adjustment techniques used that are likely to correct for these issues?	NA / Y / PY / PN / **N** / NI	
	**Risk of bias judgement**	**Low**	**There were deviations from intended exposure (for example severity of diabetes), but their impact on the outcome is expected to be slight.**
	Optional: What is the predicted direction of bias due to departures from the intended exposures?	**Favours experimental**	Undiagnosed diabetics might be classified as a non-diabetic group, which is favours the experimental (exposure DM) group.
Bias due to missing data	**5.1 Were there missing outcome data?**	Y / PY / PN / **N** / NI	Only because some participants had no complete data regarding missing teeth and diabetes; 97% of the sample enrolled, but this is prior at the start of the study.

	**5.2 Were participants excluded due to missing data on exposure status?**	Y / PY / PN **/ N** / NI	Only because they had no complete data regarding missing teeth and diabetes.
	**5.3 Were participants excluded due to missing data on other variables needed for the analysis?**	Y / PY / PN / **N** / NI	See above.
	**5.4 If Y/PY to 5.1, 5.2 or 5.3: Are the proportion of participants and reasons for missing data similar across exposures?**	NA / **Y** / PY / PN / N / NI	Yes, because sampling is prior of the study.
	**5.5 If Y/PY to 5.1, 5.2 or 5.3: Were appropriate statistical methods used to account for missing data?**	NA / Y / PY / PN / N / **NI**	Not known if there was no information about missing teeth it was more in the DM/no-DM group.
	**Risk of bias judgement**	**Low**	Data were reasonably complete. Reported missing data is prior at the start of the study.
Bias in measurement of outcomes	**6.1 Could the outcome measure have been influenced by knowledge of the exposure received?**	Y / PY / PN / **N** / NI	Data is collected from a big community-based cohort study. Respondents not known the aim of this specific study about tooth loss.
	**6.2 Was the outcome measure sensitive?**	Y / PY / PN / N / NI	The outcome measurement is a surrogate/hard endpoint and not sensitive (for change).
	**6.3 Were outcome assessors unaware of the exposure received by study participants?**	Y / PY / PN / N / **NI**	
	**6.4 Were the methods of outcome assessment comparable across exposure groups?**	Y / PY / PN / N / NI	Yes, trained and calibrated dental examiners (n=13 across all four study sites) performed a comprehensive oral examination for dental caries and periodontal disease using full-mouth assessments.

	**6.5 Were any systematic errors in measurement of the outcome unrelated to exposure received?**	Y / PY / PN / N / NI	If there were any systematic error in measurement it will be the same chance in both groups.
	**Risk of bias judgement**	**Low**	**The methods of outcome assessment were comparable across exposure groups.**
Bias in selection of the reported result	Is the reported effect estimate likely to be selected, on the basis of the results, from...?		
	**7.1. ... multiple outcome *measurements* within the outcome domain?**	Y / PY / PN / N / NI	No.
	**7.2 ... multiple *analyses* of the exposure-outcome relationship?**	Y / PY / PN / N / NI	No.
	**7.3 ... different *subgroups*?**	Y / PY / PN / N / NI	Only stratified by gender to assess its association with diabetes. Diabetes is given in subgroups:<br>- Prediabetes<br>- Diabetes (controlled/uncontrolled)
	**Risk of bias judgement**	**Low**	**There is no risk of selective reporting on the basis of results.**
Overall bias	**Risk of bias judgement**	**Low**	**Appropriate adjustment for most of the confounding areas. Thereby, the examiners determined tooth status by visual examination and diabetes on HbA1c levels based on a reliable source (ADA guideline).**

Online Appendix S2-4

Preliminary tool for risk of bias in exposure study: Costa et al. 2013/2011

Title: Progression of Periodontitis and Tooth Loss Associated with Glycemic Control in Individuals Undergoing Periodontal Maintenance Therapy: A 5-Year Follow-Up Study.

Year: 2013

Authors: Fernando Oliveira Costa, Luís Otávio Miranda Cota , Eugênio José Pereira Lages, Alcione Maria Soares Dutra Oliveira, Peterson Antônio Dutra Oliveira, Renata Magalhães Cyrino, Telma Campos Medeiros Lorentz, Sheila Cavalca Cortelli, José Roberto Cortelli

Department of Periodontology, Federal University of Minas Gerais, Belo Horizonte, Brazil.

PMID: 22769441

Title: Progression of periodontitis in a sample of regular and irregular compliers under maintenance therapy: A 3-Year Follow-Up Study.

Year: 2011

Authors: Fernando Oliveira Costa, Luís Otávio Miranda Cota, Eugênio José Pereira Lages, Telma Campos Medeiros Lorentz, Alcione Maria Soares Dutra Oliveira, Peterson Antônio Dutra Oliveira, José Eustáquio Costa.

Department of Periodontology, Federal University of Minas Gerais, Belo Horizonte, Brazil.

PMID: **21342000**

AIM: *The aim of the study is to evaluate associations between glycaemic control status and progression of periodontitis and tooth loss among individuals during periodontal maintenance therapy (PMT)*

Costa et al. 2013 and Costa et al. 2011 published data in different papers concerning one and the same population. In order to avoid including the same subjects reported in different papers, the paper of Costa et al. 2013 was chosen as representative for risk of bias.

Specify a target experiment specific to the study:

☐ The protocol-specified target experiment fully applies *OR*

Participant	**Participant**<br>92 individuals, all recruited from a prospective cohort with 238 undergoing periodontal maintenance therapy, participated in the study. This is the cohort that is used in the study Costa et al. 2011.
Experimental exposure	**Experimental exposure**<br>Diabetes: percentage of glycated hemoglobin (HbA1c)<br>- 23 individuals with DM and poor glycemic control (PGC)<br>- 23 individuals with DM and good glycemic control (GGC)
Control exposure	**Control exposure**<br>Healthy subjects = 46 (NDC)

Specify the outcome

Specify which outcome is being assessed for risk of bias (typically from among those earmarked for the Summary of Findings table). Specify whether this is a proposed benefit or harm of exposure.

Periodontal examination: plaque index, pocket depth, mean clinical attachment loss, BOP (yes/no), furcation involvement, and number of teeth present.

Is your aim for this study…?

■ to assess the effect of initiating intervention (as in an intention-to-treat analysis)

☐ to assess the effect of initiating and adhering to intervention (as in a per-protocol analysis)

☐ other (specify)

Specify the numerical result being assessed

In case of multiple alternative analyses being presented, specify the numeric result (e.g. RR = 1.52 (95% CI 0.83 to 2.77) and/or a reference (e.g. to a table, figure or paragraph) that uniquely defines the result being assessed.

Statistical analysis included a descriptive characterization of the sample according to variables of interest. Group comparisons by means of x^2 test, ANOVA, and Student t-test were performed when appropriate. When equal variances were assumed, variables were compared by means of ANOVA and Bonferroni post hoc test. When equal variances were not assumed, variables were compared by means of the Welch test and Tamhane post hoc test.

Logistic regression analysis was performed to investigate the association between the progression of periodontitis and tooth loss for the following independent predictor risk variables:

- sex (male/female)
- age (up to 30, 31 to 40, 41 to 49, and > 50 years)
- education level (> 8 years)
- cohabitation status (companion/no companion)
- smoking status (smoker/ former smoker/non-smoker)

- number of PMT visits, BMI (>25 kg/m^2)
- HbA1c (‡6.5% indicating PGC; <6.5% indicating GGC, and patients without diabetes as reference)
- duration of DM (>10 years)
- BOP (in >30% of sites),
- PD ‡4 mm in >30% of sites,
- PD between 4 and 6 mm in < 10% of sites
- clinical AL > 3 mm in 30% of sites.

All predictive variables presenting a P value of < 0.25 in the univariate analysis were included in the multivariate regression model. Variables were then removed manually step by step until the log-likelihood ratio test indicated that no variable should be removed. Confounding variables were identified if their removal from the model caused changes >15% in the b coefficient (probability of type II error). All variables included in the final multivariate model were determined to be independent through assessment of their collinearity. PI was excluded from the final model because of its covariance with BOP, and the number of remaining teeth was determined to be a covariable due to its association with other predictive variables.

Odds ratio (OR) estimates and their confidence intervals were calculated and reported. All tests were performed using statistical software. Results were considered significant if a P value <5% was attained (P <0.05).

Preliminary consideration of confounders

Complete a row for each important confounding area (i) listed in the review protocol; and (ii) relevant to the setting of this particular study, or which the study authors identified as potentially important. *"Important" confounding areas are those for which, in the context of this study, adjustment is expected to lead to a clinically important change in the estimated effect of the exposure. "Validity" refers to whether the confounding variable or variables fully measure the area, while "reliability" refers to the precision of the measurement (more measurement error means less reliability).*

(v) Confounding areas listed in the review protocol

Confounding area	Measured variable(s)	Is there evidence that controlling for this variable was unnecessary?*	Is the confounding area measured validly and reliably by this variable (or these variables)?	OPTIONAL: Is adjusting for this variable (alone) expected to move the effect estimate up or down?
Demographic factors	Sex, age	No evidence that controlling was unnecessary.	Yes	
Social/economic status	Smoking status	No evidence that controlling was unnecessary.	smokers reported consumption of > 100 cigarettes in their life and smoked at the time of examination; former smokers reported consumption of > 100 cigarettes in their life and did not smoke in at least two PMT visits; and non-smokers never smoked*.<br><br>* Turesky S, Gilmore ND, Glickman I. Reduced plaque formation by the chloromethyl analogue of victamine C. J Periodontol 1970;41:41-43.	
	Body Mass Index: BMI	No evidence that controlling was unnecessary.	Yes.	

(vi)	Additional confounding areas relevant to the setting of this particular study, or which the study authors identified as important			
**Confounding area**	**Measured variable(s)**	**Is there evidence that controlling for this variable was unnecessary? ***	**Is the confounding area measured validly and reliably by this variable (or these variables)?**	**OPTIONAL: Is adjusting for this variable (alone) expected to move the effect estimate up or down?**
Social/economic status	Education level, cohabitation status	No evidence that controlling was unnecessary.	Yes	

*In the context of a particular study, variables can be demonstrated not to be confounders and so not included in the analysis: (a) if they are not predictive of the outcome; (b) if they are not predictive of exposure; or (c) because adjustment makes no or minimal difference to the estimated effect of the primary parameter. Note that “no statistically significant association” is not the same as “not predictive”.

Preliminary consideration of criteria used to determine the accuracy of measurement of exposure and outcome

Complete a row for each measure listed in the study for the (i) exposure and (ii) outcome. Of the measures listed in the protocol, consider the sensitivity, specificity, and confidence in the methods used in the study.

(v) Exposure measurement method listed in the study		
Method of measurement	Measured exposure	Is the exposure measured validly and reliably by this method (or these methods)?
Professionally diagnosed (percentage HbA1c at all visits of PMT)	Type II DM	Yes: the percentage HbA1c is measured at all visits of PMT. After 5 years of PMT the individuals were selected and were allocated over the groups.<br><br>Definition of Type 2 DM and cut-off points regarding HbA1c percentages were based on American Diabetes Association (ADA) parameters. HbA1c tests were performed on all patients with diabetes 1 to 3 days before the PMT visit, in two referral laboratories, according to each individual's choice and convenience. These tests were performed according to the international standards recommended by the ADA*.<br><br>**American Diabetes Association. Diagnosis and classification of diabetes mellitus. Diabetes Care 2011;34 (Suppl. 1): S62-S69.*

(vi) Outcome measurement method listed in the study		
Method of measurement	Measured outcome	Is the outcome measured validly and reliably by this method (or these methods)?
Periodontal examination/evaluation (during each PMT visit)	Plaque Index, PD, clinical AL, BOP, furcation involvement, SU and tooth loss.	All periodontal parameters were used to determine periodontal status in four sites per tooth. Examinations were performed with a manual periodontal probe*.<br><br>Methodology for data collection and periodontal clinical procedures during all PMT visits were the same as reported by Lorentz et al**.

		* University of North Carolina (PCPUNC15BR) and Nabers PQ2NBR, Hu- Friedy, Chicago, IL.<br><br>** Lorentz TC,CotaL O,Cortelli JR,Vargas AM,Costa FO. Prospective study of complier individuals under periodontal maintenance therapy: Analysis of clinical periodontal parameters, risk predictors and the progression of periodontitis. J Clin Periodontol 2009;36: 58-67.
Interview (during each PMT visit)		To determine possible changes in variables of interest (demographic, biologic and behavioural)
Application of disclosing agents	Plaque index	

All interviews, examinations, and clinical periodontal procedures were performed by two trained and calibrated periodontists.

Preliminary consideration of co-exposures

Complete a row for each important co-intervention (i) listed in the review protocol; and (ii) relevant to the setting of this particular study, or which the study authors identified as important.
"Important" co-interventions are those for which, in the context of this study, adjustment is expected to lead to a clinically important change in the estimated effect of the intervention.

**(v) Co-exposures listed in the review protocol**		
Co-exposure	Is there evidence that controlling for this co-exposure was unnecessary (e.g., because it was not administered)?	Is presence of this co-exposure likely to favor outcomes in the experimental or the control group
		Favor experimental / Favor comparator / No information
		Favor experimental / Favor comparator / No information
		Favor experimental / Favor comparator / No information

**(vi) Additional co-exposures relevant to the setting of this particular study, or which the study authors identified as important**		
Co-exposure	Is there evidence that controlling for this co-exposure was unnecessary (e.g., because it was not administered)?	Is presence of this co-exposure likely to favor outcomes in the experimental or the control group
		Favor experimental / Favor comparator / No information
		Favor experimental / Favor comparator / No information
		Favor experimental / Favor comparator / No information

Risk of bias assessment (cohort-type studies)

Bias due to confounding	**1.1 Is there potential for confounding of the effect of exposure in this study? If N or PN to 1.1:** the study can be considered to be at low risk of bias due to confounding and no further signalling questions need be considered	**Y** / PY / PN / N	Yes, due the case-control design nested in an open cohort.<br>- Demographic factors: sex, age<br>- Social/economic factors: cohabitation status, education level, smoking level<br>- BMI
	**If Y/PY to 1.1, answer 2.1 and 1.3 to determine whether there is a need to assess time-varying confounding:**		
	**1.2. If Y or PY to 1.1: Was the analysis based on splitting, follow up time according to exposure received?**<br>**If N or PN to 1.2, answer questions 1.4 to 1.6, which relate to baseline confounding**	NA / Y / PY / PN / N / NI	The participants could not switch between exposures, so the outcome could not be biased due time varying confounding.
	**1.3. If Y or PY to 1.2: Were exposure discontinuations or switches likely to be related to factors that are prognostic for the outcome?**	NA / Y / PY / PN / **N** / NI	It is possible that there will be undiagnosed diabetics in the non-diabetic (control) group due of misclassification, but not likely to happen because after 5 years PMT the individuals were selected based on HbA1c control.
	**If N or PN to 1.3**, answer questions 1.4 to 1.6, which relate to baseline confounding		
	**1.4. Did the authors use an appropriate analysis method that adjusted for all the critically important confounding areas?**	NA / Y / PY / PN / N / NI	Logistic regression analysis performed to investigate the association between the progression and tooth loss for independent predictor risk variables.

	**1.5. If Y or PY to 1.4: Were confounding areas that were adjusted for measured validly and reliably by the variables available in this study?**	NA / Y / PY / PN / N / NI	Yes<br><br>All predictive variables presenting a P value of < 0.125 in the univariate analysis were included in the multivariate regression model. Variables were then removed manually step by step until the log-likelihood ratio test indicated that no variable should be removed. Confounders were included and identified if their removal from the model caused changes >15% in the b coefficient (type II error?)
	**1.6. Did the authors avoid adjusting for post-exposure variables?**	NA / Y / PY / PN / N / NI	No information about post-exposure variables.
	**If Y or PY to 1.3**, answer questions 1.7 and 1.8, which relate to time-varying confounding		

	**1.7. Did the authors use an appropriate analysis method that adjusted for all the critically important confounding areas and for time-varying confounding?**	NA / Y / PY / PN / N / NI	Yes, the authors used data after 5 years of PMT visits.
	**1.8. If Y or PY to 1.7: Were confounding areas that were adjusted for measured validly and reliably by the variables available in this study?**	NA / Y / PY / PN / N / NI	Previous studies showed that the potential confounders possibly tended to bias the outcome.
	**Risk of bias judgement**	**Moderate**	**A detailed description about the identifying confounding procedure and thereafter adjustment for potential confounders. A multivariate regression model was applied to evaluate the periodontitis and tooth loss association after controlling for confounders.**

Bias in selection of participants into the study	**2.1. Was selection of participants into the study (or into the analysis) based on variables measured after the start of the exposure?**<br><br>**<u>If N or PN to 2.1 go to 2.4</u>**	**Y** / PY / PN / N / NI	First:<br>Individuals were excluded from the study if they:<br><br>- were pregnant (n = 3);<br>- showed debilitating dis-eases that could impair the immune system (such as human immunodeficiency virus/acquired immune-deficiency syndrome, cancer, and auto-immune diseases; n = 4);<br>- presented with drug-induced gingival hyperplasia (n = 6)<br>- had Type I DM (n = 3);<br>- had <14 teeth (n = 19);<br>- Were irregular compliers (intervals between PMT visits >12 months [n = 53])<br>- showed glycemic status oscillation between >6.5% and <6.5% of glycated hemoglobin (HbA1c) in at least two PMT visits (n = 4).<br><br>Second and based on the above criteria after a period of 5 years of PMT the individuals were selected.
	**2.2. <u>If Y/PY to 2.1:</u> Were the post-exposure variables that influenced selection associated with exposure?**	Y / PY / PN / N / **NI**	No information.
	**2.3. <u>If Y/PY to 2.2:</u> Were the post-exposure variables that influenced eligibility selection influenced by the outcome or a cause of the outcome?**	NA/ **Y**/ PY / PN / N / NI	Yes, the authors selected the individuals after a period of 5 years of PMT.
	**2.4 Do start of follow-up and start of exposure coincide for most participants?**	NA / **Y** / PY / PN / N / NI	The start of the exposure could change between participants, but

			clinical periodontal procedures were performed at the same time for the whole cohort.
	2.5 If Y/PY to 2.2 and 2.3, or N/PN to 2.4: Were adjustment techniques used that are likely to correct for the presence of selection biases?	NA / Y / PY / PN / **N** / NI	No techniques were used that correct fort he presence of selection bias.
	**Risk of bias judgement**	**Moderate**	**Individuals were selected in a clearly defined two-step screening criteria, but the use of this prospective (5-year follow up) design could indicate the possibility of selection bias.**
Bias in classification of exposures	3.1 Is exposure status well defined?	Y / PY / PN / N / NI	Yes, clearly defined following the ADA parameters. Based on HbA1c percentage at all visits of PMT during a period of 5 years.
	3.2 Did entry into the study begin with start of the exposure?	Y / PY / PN / N / NI	Some of the participants could been exposed for DM for a longer time than others. Therefore, the authors made subgroups for controlling diabetes (poor glycemic control/good glycemic control).<br><br>The possibility that there will be undiagnosed diabetics in the non-diabetic (control) group is not likely because of the 5 year follow up design.
	3.3 Was information used to define exposure status recorded prior to outcome assessment?	**Y** / PY / PN / N / NI	ADA parameters were used to define exposure status before dental examination.
	3.4 Could classification of exposure status have been affected by knowledge of the outcome or risk of the outcome?	Y / PY / PN / **N** / NI	No, HBA1c test were performed on all patients with diabetes 1 to 3 days before PMT visits, into referral laboratories.

	3.5 Were exposure assessment methods robust (including methods used to input data)?	**Y** / PY / PN / N / NI	Yes, see above 3.4
	**Risk of bias judgement**	**Low**	**Diabetes was properly assessed according to the percentage of HbA1c percentages prior at all visits of PMT during a period of 5 years based on ADA parameters. Thereafter, the test was performed in two referral laboratories.**
Bias due to departures from intended exposures	**4.1. Is there concern that changes in exposure status occurred among participants?**<br><br>**If your aim for this study is to assess the effect of initiating and adhering to an exposure (as in a per-protocol analysis), answer questions 4.2 and 4.3, otherwise continue to 4.4 if Y or PY to 4.1.**	Y / PY / PN / N / NI	Not likely, because the follow-up period of 5 years.
	**4.2. Did many participants switch to other exposures?**	Y / PY / PN / **N** / NI	It is possible that the control of diabetes changes over time. Partly because this the authors made 2 subgroups for controlling diabetes (poor and good controlled)
	**4.3. Were the critical co-exposures balanced across exposure groups?**	Y / PY / PN / N / NI	Prior the whole group is matched for sex and smoking.<br><br>The groups were matched in an attempt to minimize the effects of confounders: if more than one match could be achieved, GGC and NDC individuals were randomly selected among the matches.

	**4.4. If NY/PN PY to 4.1, or Y/PY to 4.2, or 4.3: Were adjustment techniques used that are likely to correct for these issues?**	NA / **Y** / PY / PN / N / NI	Matching
	**Risk of bias judgement**	**Low**	**There were some deviations from usual practice, but their impact on the outcome is expected to be slight because the authors used proper matching techniques to minimize the effects of potential confounders.**
Bias due to missing data	**5.1 Were there missing outcome data?**	Y / PY / PN / **N** / NI	Missing outcome data during or after analysis were not reported.
	**5.2 Were participants excluded due to missing data on exposure status?**	Y / PY / PN / **N** / NI	All the participants included for analysis performed HbA1c test during a period of 5-year prior at the start of the study.
	**5.3 Were participants excluded due to missing data on other variables needed for the analysis?**	Y / PY / PN / N / **NI**	No information.
	**5.4 If Y/PY to 5.1, 5.2 or 5.3: Are the proportion of participants and reasons for missing data similar across exposures?**	NA / Y / PY / PN / N / NI	No information.
	**5.5 If Y/PY to 5.1, 5.2 or 5.3: Were appropriate statistical methods used to account for missing data?**	NA / Y / PY / PN / N / NI	Not necessary, because no missing data is reported.
	**Risk of bias judgement**	**Low**	**Data is complete.**
Bias in measurement of outcomes	**6.1 Could the outcome measure have been influenced by knowledge of the exposure received?**	Y / PY / PN / **N** / NI	All interviews, examinations and clinical periodontal procedures were performed by two trained and calibrated periodontists separate from the HbA1c test in the two referral laboratories.

	**6.2 Was the outcome measure sensitive?**	Y / PY / PN / N / NI	Specifically, the outcome tooth loss is<br>is a surrogate/hard endpoint and not sensitive (for change).<br><br>For the other periodontal procedures:<br>- PD+AL: were recorded and repeated within a 1-week interval for 10 individuals randomly selected from all study groups at baseline and final examination.<br><br>Data were tested through non-parametric k test and intraclass correlation. k coefficients for both intra- and interexaminer, as well as intraclass correlation coefficients were >0.87.<br><br>Before the study, a training process was conducted to standardize the application of smoking questionnaires during interviews. Interviews were repeated on 12 individuals to verify the quality of categorical data obtained. Because the literature has reported high inconsistency and biased information regarding smoking special attention was given to questions related to smoking habits. k coefficients obtained for smoking were 0.89.<br><br>In addition, all data collected by the questionnaire that may have changed with time were confirmed at each PMT visit.

	**6.3 Were outcome assessors unaware of the exposure received by study participants?**	Y / PY / PN / N / **NI**	No information.
	**6.4 Were the methods of outcome assessment comparable across exposure groups?**	Y / PY / PN / N / NI	Yes, all the participants underwent the same interviews, examinations and clinical periodontal procedures.
	**6.5 Were any systematic errors in measurement of the outcome unrelated to exposure received?**	Y / PY / PN / N / NI	Unlikely, see 6.2.
	**Risk of bias judgement**	**Low**	**The methods of outcome assessment were comparable across exposure groups.**
Bias in selection of	Is the reported effect estimate likely to be selected, on the basis of the results, from...?		
the reported result	**7.1. ... multiple outcome *measurements* within the outcome domain?**	Y / PY / PN / **N** / NI	Fully reported for plaque, BOP, mean clinical AL, pocket depth, number of teeth and SU. Furcation involvement is not described.
	**7.2 ... multiple *analyses* of the exposure-outcome relationship?**	**Y** / PY / PN / N / NI	Fully reported
	**7.3 ... different *subgroups*?**	**Y** / PY / PN / N / NI	Fully reported, subgroups are made in controlling diabetes (poor/good controlled).
	**Risk of bias judgement**	**Low**	**Furcation involvement is performed during periodontal examination but not fully described. This is not the aim of the conducted SR so risk of bias in the selection of reported results is despite this low.**
Overall bias	**Risk of bias judgement**	**Low**	**Some limitations, including the absence of stratified analysis of dose exposures and the small sample size of individuals for the final multivariable analysis.**

			**However, the 5-year follow up, prospective design, matching for smoking and gender, adjustment for potential confounders and standardization of periodontal treatment and PMT may minimalize these issues.**

Online Appendix S2-5

Preliminary tool for risk of bias in exposure study: Patel et al. 2013

Title: diabetes and tooth loss. An analysis of data from the National Health and Nutrition Examination Survey, 2003-2004.

Year: 2013

Authors: Manthan H. Patel, BDS, MPH; Jayanth V. Kumar, DDS, MPH; Mark E. Moss, DDS, MS, PhD.

PMID: 23633695

AIM*: to understand the association between diabetes and tooth loss in the united states.*

- *Conduct an analysis from the National Health and Nutrition Examination Survey (NHANES) to understand these relationships.*

Specify a target experiment specific to the study:

☐ The protocol-specified target experiment fully applies *OR*

Participant

Experimental exposure

Control exposure

Participant:
NHANES data for the 2003-2004 cycle → National Center for Health Statistics (NCHS) of the Centers for Disease Control and Prevention (CDC)
Inclusion 2508 participants who were 50 years and older for whom were oral health status and self-reported diabetes data.

NHANES investigators surveyed 10,122 people during the 2003-2004 cycle. Of these participants, only 2,508 were 50 years and older, which represented 77 million noninstitutionalized civilian people in the United States.
N= 2508
Edentulous: 453 participants
Dentulous: 2055 participants

Experimental exposure:
N= 384

Control exposure:
N= 1671

Specify the outcome

Specify which outcome is being assessed for risk of bias (typically from among those earmarked for the Summary of Findings table). Specify whether this is a proposed benefit or harm of exposure.

Tooth loss was the principal outcome of interest.

They calculated the prevalence of edentulism and the number of missing teeth among dentate people 50 years and older.

Is your aim for this study…?

■ to assess the effect of initiating intervention (as in an intention-to-treat analysis)

☐ to assess the effect of initiating and adhering to intervention (as in a per-protocol analysis)

☐ other (specify)

Specify the numerical result being assessed

In case of multiple alternative analyses being presented, specify the numeric result (e.g. RR = 1.52 (95% CI 0.83 to 2.77) and/or a reference (e.g. to a table, figure or paragraph) that uniquely defines the result being assessed.

Calculating various statistics and estimating standard errors (SEs) by using the survey module. The complex design of

MEC examination statistical weight with cluster and strata variables provided by NHANES to calculate weighted
point estimates and standard errors. Weighted numbers account for the complex survey design (including oversampling), survey nonresponse and poststratification (that is, the number of people in the population represented by that sample person).

Descriptive and weighted analyses to describe the prevalence of edentulism and the number of missing teeth among people with and without diabetes.
Statistical significance for the differences in the prevalence of edentulism over the different exposure categories by using c2 tests.
T test: to measure the mean differences in the number of missing teeth between dentate people with and without diabetes.
Logistic regression: to determine the effect of diabetes on edentulism.

Calculation of the adjusted odds ratio (OR) and regression coefficients along with 95 percent confidence interval (CI) to report statistical significance
at .05 level.
Multiple linear regression to control for the effect of potential confounding factors on the total number of missing teeth.

MEC examination weight provided by NHANES29 to obtain results that are representative of the U.S. population 50 years and older. We excluded from the multivariate analyses participants with missing values. In addition, we estimated the population attributable risk (PAR) percentage in a cross-sectional study by using the Levin31 formula
under the assumption that the diagnosis of diabetes preceded the outcome of edentulism.

Preliminary consideration of confounders

Complete a row for each important confounding area (i) listed in the review protocol; and (ii) relevant to the setting of this particular study, or which the study authors identified as potentially important. *"Important" confounding areas are those for which, in the context of this study, adjustment is expected to lead to a clinically important change in the estimated effect of the exposure. "Validity" refers to whether the confounding variable or variables fully measure the area, while "reliability" refers to the precision of the measurement (more measurement error means less reliability).*

(vii) Confounding areas listed in the review protocol

Confounding area	Measured variable(s)	Is there evidence that controlling for this variable was unnecessary? *	Is the confounding area measured validly and reliably by this variable (or these variables)?	OPTIONAL: Is adjusting for this variable (alone) expected to move the effect estimate up or down?
Demographic factors	Age, sec, race/ethnicity	No, based on previous epidemiologic studies*	Yes<br>Age: univariate analysis and considered as a continuous variable during model analysis.	Favor intervention / Favor control / No information
Social/economic status	Family income, dental insurance	No, based on previous epidemiologic studies*	Dental insurance: considered as a dichotomous variable.	
Behaviour	Smoking (history)	No, based on previous epidemiologic studies*	Number of days the person smoked to assign each participant to a categories: never, light or heavy smoker.	

(viii) **Additional confounding areas relevant to the setting of this particular study, or which the study authors identified as important**

Confounding area	Measured variable(s)	Is there evidence that controlling for this variable was unnecessary? *	Is the confounding area measured validly and reliably by this variable (or these variables)?	OPTIONAL: Is adjusting for this variable (alone) expected to move the effect estimate up or down?
Social status	Education	No, based on previous epidemiologic studies*	Yes / No / No information	Favor intervention / Favor control / No information

*Previous epidemiologic studies:

1. Kapp JM, Boren SA, Yun S, LeMaster J. Diabetes and tooth loss in a national sample of dentate adults reporting annual dental visits. Prev Chronic Dis 2007;4(3):A59.
2. Medina-Solís CE, Pérez-Núñez R, Maupomé G, Casanova- Rosado JF. Edentulism among Mexican adults aged 35 years and older and associated factors (published online ahead of print June 29, 2006). Am J Public Health 2006;96(9):1578-1581. doi:10.2105/AJPH.2005.071209.

* In the context of a particular study, variables can be demonstrated not to be confounders and so not included in the analysis: (a) if they are not predictive of the outcome; (b) if they are not predictive of exposure; or (c) because adjustment makes no or minimal difference to the estimated effect of the primary parameter. Note that "no statistically significant association" is not the same as "not predictive".

Preliminary consideration of criteria used to determine the accuracy of measurement of exposure and outcome

Complete a row for each measure listed in the study for the (i) exposure and (ii) outcome. Of the measures listed in the protocol, consider the sensitivity, specificity, and confidence in the methods used in the study.

(vii) Exposure measurement method listed in the study		
Method of measurement	Measured exposure	Is the exposure measured validly and reliably by this method (or these methods)?
Diabetes questionnaire	(Self-reported) diabetes status	No information about the validity of the questionnaire.<br>Asked by trained interviewers in their homes:<br>- Have you ever been told by a doctor or health care professional that you have diabetes or sugar diabetes?"<br>Answer: yes, no or borderline.<br><br>We categorized the participants with borderline

		diabetes as having diabetes, whereas we excluded from the analyses those who responded "refused" - and "don't know." We divided the presence

(viii) Outcome measurement method listed in the study		
Method of measurement	Measured outcome	Is the outcome measured validly and reliably by this method (or these methods)?
Oral examination	Tooth loss	Yes: performed by trained dental examiners in mobile examination centers (MEC). Tooth loss count separately for each tooth space and reported the participant of having a primary tooth, a permanent tooth, a dental implant, a root tip or an absent tooth.
Oral examination	Edentulous	Missing all permanent teeth.

Preliminary consideration of co-exposures

Complete a row for each important co-intervention (i) listed in the review protocol; and (ii) relevant to the setting of this particular study, or which the study authors identified as important.
"Important" co-interventions are those for which, in the context of this study, adjustment is expected to lead to a clinically important change in the estimated effect of the intervention.

**(vii)** **Co-exposures listed in the review protocol**		
Co-exposure	Is there evidence that controlling for this co-exposure was unnecessary (e.g., because it was not administered)?	Is presence of this co-exposure likely to favor outcomes in the experimental or the control group
		Favor experimental / Favor comparator / No information
		Favor experimental / Favor comparator / No information
		Favor experimental / Favor comparator / No information

**(viii)** **Additional co-exposures relevant to the setting of this particular study, or which the study authors identified as important**		
Co-exposure	Is there evidence that controlling for this co-exposure was unnecessary (e.g., because it was not administered)?	Is presence of this co-exposure likely to favor outcomes in the experimental or the control group
		Favor experimental / Favor comparator / No information
		Favor experimental / Favor comparator / No information
		Favor experimental / Favor comparator / No information

Risk of bias assessment (cohort-type studies)

Bias due to confounding	**1.1 Is there potential for confounding of the effect of exposure in this study? If N or PN to 1.1: the** study can be considered to be at low risk of bias due to confounding and no further signaling questions need be considered	**Y** / PY / PN / N	Cross sectional design did not account for temporality—that is, whether diabetes preceded tooth loss in the participants. Potential confounding of the effect of the exposure in the study.
	**If Y/PY to 1.1, answer 2.1 and 1.3 to determine whether there is a need to assess time-varying confounding:**		
	**1.2. If Y or PY to 1.1: Was the analysis based on splitting follow up time according to exposure received?** **If N or PN to 1.2**, answer questions 1.4 to 1.6, which relate to baseline confounding	NA / Y / PY / PN / N / NI	The participants could not switch between exposures, so the outcome could not be biased due time varying confounding.
	**1.3. If Y or PY to 1.2: Were exposure discontinuations or switches likely to be related to factors that are prognostic for the outcome?**	NA / Y / PY / PN / N / NI	Yes, because diabetes mellitus (type II) is self-reported at one point (previously) in the study. It is possible that there will be undiagnosed diabetics in the non-diabetic (control) group.
	**If N or PN to 1.3**, answer questions 1.4 to 1.6, which relate to baseline confounding		
	**1.4. Did the authors use an appropriate analysis method that adjusted for all the critically important confounding areas?**	NA / Y / PY / PN / N / NI	Yes: multiple logistic regression analysis: age, sex, race/ ethnicity, education, family income, dental insurance and history of smoking.
	**1.5. If Y or PY to 1.4: Were confounding areas that were adjusted for measured validly and reliably by the variables available in this study?**	NA / Y / PY / PN / N / NI	Yes, the potential confounders are selected on the basis of results from previous epidemiologic studies.

	**1.6. Did the authors avoid adjusting for post-exposure variables?**	NA / Y / PY / PN / N / **NI**	No information about post-exposure variables.
	**If Y or PY to 1.3**, answer questions 1.7 and 1.8, which relate to time-varying confounding		

	**1.7. Did the authors use an appropriate analysis method that adjusted for all the critically important confounding areas and for time-varying confounding?**	NA / Y / PY / PN / N / NI	No, the results are measured at one point at the time, there is no information about time varying.
	**1.8. If Y or PY to 1.7: Were confounding areas that were adjusted for measured validly and reliably by the variables available in this study?**	NA / Y / PY / PN / N / NI	Yes, the potential confounders are selected on the basis of results from previous epidemiologic studies.
	**Risk of bias judgement**	**Low**	**Confounding expected, all known important confounding domains appropriately measured and controlled for by using multiple linear regression models.**
Bias in selection of participants into the study	**2.1. Was selection of participants into the study (or into the analysis) based on variables measured after the start of the exposure?**<br><br>**<u>If N or PN to 2.1 go to 2.4</u>**	Y / PY / PN / N / NI	NHANES uses a complex, stratified, multistage probability sample to measure the health and nutritional status of adults and children who represent the civilian, noninstitutionalized population in the United States.<br><br>Detailed description of selection of participants is provided elsewhere.<br><br>Decision for a specific age group > 60 years, because tooth loss is more prevalent in people 50 years and older. Hence, the authors restricted the analysis to this age group.
	**2.2. <u>If Y/PY to 2.1:</u> Were the post-exposure variables that influenced selection associated with exposure?**	Y / PY / PN / N / NI	There could be selection bias, because the sample is based on diabetes questionnaire.

			If diabetes is misclassified than it is likely that the true association between tooth loss is diluted.
	**2.3. If Y/PY to 2.2: Were the post-exposure variables that influenced eligibility selection influenced by the outcome or a cause of the outcome?**	NA / Y / PY / PN / **N** / NI	The study provided a valid population-based estimate that can be generalized to the US population 50 years and older.
	**2.4 Do start of follow-up and start of exposure coincide for most participants?**	NA / Y / PY / PN / N / **NI**	No information.
	**2.5 If Y/PY to 2.2 and 2.3, or N/PN to 2.4: Were adjustment techniques used that are likely to correct for the presence of selection biases?**	NA / Y / PY / PN / **N** / NI	No.
	**Risk of bias judgement**	**Low**	**Representative sample of the general U.S. population. The authors described in the discussion section of the article: ‘’because of oversampling of people 60 years and older in the NHANES cycle for 2003-2004, the estimates for this age group are more precise and reliable than those in previous studies.**
Bias in classification of exposures	**3.1 Is exposure status well defined?**	Y / PY / PN / N / NI	Diabetes questionnaire to assess the participants self-reported diabetes status by trained interviewers.<br><br>Have you ever been told by a doctor or health care professional that you have diabetes or sugar diabetes?”<br>- Answer: yes, no or borderline.<br>- Borderline: having diabetes.<br>- Refused/don’t know were excluded<br>So only two groups remain.

			1. Participants reported with DM<br>2. Participants reporting, they did not had diabetes.<br><br>If diabetes is misclassified than it is likely that the true association between tooth loss is diluted.
	3.2 Did entry into the study begin with start of the exposure?	**Y** / PY / PN **/** N / NI	Participants classified after the diabetes questionnaire into two groups:<br>1. Participants reported with DM<br>2. Participants reporting, they did not had diabetes.
	3.3 Was information used to define exposure status recorded prior to outcome assessment?	Y / PY / PN / **N** / NI	Diabetes questionnaire to assess the participants self-reported diabetes status.
	3.4 Could classification of exposure status have been affected by knowledge of the outcome or risk of the outcome?	Y / PY / PN / N / NI	No classification of exposure status is assessed by participants with a diabetes questionnaire to assess self- reported diabetes status. Tooth loss is examined by dental trained examiners separately.
	3.5 Were exposure assessment methods robust (including methods used to input data)?	Y / PY / PN / N / **NI**	
	**Risk of bias judgement**	**Moderate**	**A diabetes questionnaire to assess self-reported diabetes status is possibly subject to bias and misinterpretation. Thereby undiagnosed diabetes could be misclassified in the no-dm group. Exposure status assessment is more reliable if it is measured by a professional and classified based on Hba1 levels.**

Bias due to departures from intended exposures	**4.1. Is there concern that changes in exposure status occurred among participants?**<br><br>**If your aim for this study is to assess the effect of initiating and adhering to an exposure (as in a per-protocol analysis), answer questions 4.2 and 4.3, otherwise continue to 4.4 if Y or PY to 4.1**	Y / PY / PN / N / NI	Undiagnosed diabetics might be classified as a non-diabetic group, because the exposure status is diagnosed at 1 time previously at the start in a diabetes questionnaire. Thereby no distinction is made in severity or type of diabetes:<br><br>Self-diabetes, prediabetes, high sugar, and any conditions other than “diabetes” or “sugar diabetes” were not included during the interview.
	**4.2. Did many participants switch to other exposures?**	Y / PY / PN / N / NI	No, due the cross-sectional design.
	**4.3. Were the critical co-exposures balanced across exposure groups?**	Y / PY / PN / N / NI	No, due the cross-sectional design.
	**4.4. If NY/PN PY to 4.1, or Y/PY to 4.2, or 4.3: Were adjustment techniques used that are likely to correct for these issues?**	NA / Y / PY / PN / N / NI	It is not possible to adjust for these issues because the exposure status is collected using 1 question: “Have you ever been told by a doctor or health care professional that you have diabetes or sugar diabetes?”
	**Risk of bias judgement**	**Serious**	**There were deviations from intended exposure, because they used a diabetes questionnaire to assess the self-reported diabetes status. If DM status is misclassified the true association between DM and tooth loss is diluted.**
Bias due to missing data	**5.1 Were there missing outcome data?**	**Y** / PY / PN / N / NI	In the chapter of the statistical analysis:<br>‘’We excluded from the multivariate analyses participants with missing

			values''. No further information is given about the reason and proportion of the missing data.
	**5.2 Were participants excluded due to missing data on exposure status?**	**Y** / PY / PN / N / NI	Yes: self-diagnosed diabetes, prediabetes, high sugar and any conditions other than diabetes or sugar diabetes were not included in the interview.<br><br>Respondents answering refused and don't know were excluded from analysis. Also, woman who had diabetes only during the time of pregnancy were considered to not have diabetes.
	**5.3 Were participants excluded due to missing data on other variables needed for the analysis?**	Y / PY / PN / N / **NI**	No information, only reasons for excluding due missing data on exposure status.
	**5.4 If Y/PY to 5.1, 5.2 or 5.3: Are the proportion of participants and reasons for missing data similar across exposures?**	NA / Y / PY / PN / N / **NI**	No information, see 5.1
	**5.5 If Y/PY to 5.1, 5.2 or 5.3: Were appropriate statistical methods used to account for missing data?**	NA / Y / PY / PN / **N** / NI	No sensitivity analysis or other statistical methods are used to account for missing data.
	**Risk of bias judgement**	**Moderate**	**No specific information is described about the reason or proportion of missing data. The authors excluded participants with missing values from the multivariate analysis.**
Bias in measurement of outcomes	**6.1 Could the outcome measure have been influenced by knowledge of the exposure received?**	Y / PY / PN / N / **NI**	Data collected by trained dental examiners. This provide a valid and accurate estimate.<br><br>Not sure if the oral examiner known about the exposure status; so, if an patient had DM or not. It is unlikely

			that knowledge of the DM exposure influenced the examination for tooth loss.
	**6.2 Was the outcome measure sensitive?**	Y / PY / PN / N / NI	The outcome measurement is a surrogate/hard endpoint and not sensitive (for change).<br><br>Only permanent teeth were counted and a total of 28 teeth were considered.
	**6.3 Were outcome assessors unaware of the exposure received by study participants?**	Y / PY / PN / N / NI	They were unaware of the exposure because the outcome and the exposure status is measured by two different professionals.<br>Presence of DM: trained interviewer<br>Number of teeth: oral examiners.
	**6.4 Were the methods of outcome assessment comparable across exposure groups?**	Y / PY / PN / N / NI	Same examination to assess the number of missing teeth in both groups. No distinction is made between DM and no-DM group.
	**6.5 Were any systematic errors in measurement of the outcome unrelated to exposure received?**	Y / PY / PN / N / **NI**	No information is given about any systematic errors in measurements. Oral examiners were performed by trained dental examiners. This provides an more valid and accurate estimate relative to an inexperienced examiner (with no dental experience).
	**Risk of bias judgement**	**Low**	**The outcome measurement (tooth loss) is well defined and described. Outcome and exposure assessments is measured by two different professionals.**
Bias in selection of	Is the reported effect estimate likely to be selected, on the basis of the results, from...?		
the reported result	**7.1. ... multiple outcome *measurements* within the outcome domain?**	**Y** / PY / PN / N / NI	2 different outcomes:<br>- Prevalence of edentulism

			- Tooth loss among dentate people.<br>Both fully described.
	7.2 ... multiple *analyses* of the exposure-outcome relationship?	Y / PY / PN / N / NI	
	7.3 ... different *subgroups*?	**Y** / PY / PN / N / NI	2 different subgroups were made:<br>- Edentulous<br>- Dentulous<br>Both fully described.
	**Risk of bias judgement**	**Low**	**Data were complete.**
Overall bias	**Risk of bias judgement**	**Serious**	**Representative sample of the US population is a major strength of the study. Thereby the author adjusted for several confounding areas.**<br><br>**A limitation is the use of self-reported diabetes status. If diabetes is misclassified, then it is likely that the true association between diabetes and tooth loss is diluted.**

Online Appendix S2-6

Preliminary tool for risk of bias in exposure study: Botero et al. 2012

Title: Tooth and Periodontal Clinical Attachment Loss Are Associated with Hyperglycaemia in Patients with Diabetes.

Year: 2012

Authors: Javier Enrique Botero,* Fanny Lucia Yepes,* Natalia Rolda´n,* Cesar Augusto Castrillo´n,* Juan Pablo Hincapie,* Sandra Paola Ochoa,* Carlos Andre´s Ospina,* Mar´ıa Alejandra Becerra,* Adriana Jaramillo,† Sonia Jakeline Gutierrez,† and Adolfo Contreras†

** Faculty of Dentistry, Universidad de Antioquia, Medellin, Colombia.*

† Periodontal Medicine Group, Universidad del Valle, Cali, Colombia.

PMID: **22248217**

AIM: *assess the relationship between blood glucose levels and clinical parameters of periodontal disease in patients with diabetes*

Specify a target experiment specific to the study:

☐ The protocol-specified target experiment fully applies ***OR***

Participant	**Participant**:<br>convenience sample selected from February 2010 to March 2011
Experimental exposure	**Experimental exposure:**<br>65 individuals with DM
Control exposure	**Control exposure:**<br>81 individuals without DM: Smokers were separately analysed. However, none of the DM reported smoking. Therefore, only the non-smoking no DM were considered as control group. The controls consisted thereafter of 59 individuals.

Specify the outcome

Specify which outcome is being assessed for risk of bias (typically from among those earmarked for the Summary of Findings table). Specify whether this is a proposed benefit or harm of exposure.

Periodontal examination recorded in six sites around each tooth excluding M3's.<br>PD (millimetres)<br>CAL (millimetres)<br>Bleeding on probing (average in percentage)<br>Number of teeth<br>Radiographs to evaluated bone loss

Is your aim for this study…?

■ to assess the effect of initiating intervention (as in an intention-to-treat analysis)

☐ to assess the effect of initiating and adhering to intervention (as in a per-protocol analysis)

☐ other (specify)

Specify the numerical result being assessed

In case of multiple alternative analyses being presented, specify the numeric result (e.g. RR = 1.52 (95% CI 0.83 to 2.77) and/or a reference (e.g. to a table, figure or paragraph) that uniquely defines the result being assessed.

The relationship between periodontitis and diabetes was tested in a 2x2 table, and the OR was calculated (95% CI).<br>CAL was considered the primary outcome and PD and number of teeth present were the secondary outcomes.<br>To establish the relationship between glycemia (independent variable) and periodontal clinical parameters (dependent variable), a linear regression analysis and the Spearman correlation test were used. A statistical software§ was used to analyse all data. Statistical differences were assumed when P <0.05.

Preliminary consideration of confounders

Complete a row for each important confounding area (i) listed in the review protocol; and (ii) relevant to the setting of this particular study, or which the study authors identified as potentially important.
"Important" confounding areas are those for which, in the context of this study, adjustment is expected to lead to a clinically important change in the estimated effect of the exposure. "Validity" refers to whether the confounding variable or variables fully measure the area, while "reliability" refers to the precision of the measurement (more measurement error means less reliability).

**(ix) Confounding areas listed in the review protocol**				
Confounding area	Measured variable(s)	Is there evidence that controlling for this variable was unnecessary? *	Is the confounding area measured validly and reliably by this variable (or these variables)?	OPTIONAL: Is adjusting for this variable (alone) expected to move the effect estimate up or down?

Demographic factors	Sex, age	No evidence that controlling was unnecessary.	Yes	
Social/economic status	Smoking status	No evidence that controlling was unnecessary.	Yes: the authors mentioned that cigarette smoking is a confounding variable and therefore not considered as exclusion criterion but recorded when indicated and analysed independently	

(x) **Additional confounding areas relevant to the setting of this particular study, or which the study authors identified as important**

Confounding area	Measured variable(s)	Is there evidence that controlling for this variable was unnecessary? *	Is the confounding area measured validly and reliably by this variable (or these variables)?	OPTIONAL: Is adjusting for this variable (alone) expected to move the effect estimate up or down?
DM type	Type I or type II	Yes: type I and type II was known, the patients were considered in the same group because previous studies have found that they are equally susceptible to periodontal disease.<br><br>*Emrich LJ, Shlossman M, Genco RJ. Periodontal disease in non-insulin-dependent diabetes mellitus. J Periodontol 1991;62:123-131.*<br><br>*Ryan ME, Carnu O, Kamer A. The influence of di- abetes on the periodontal tissues. J Am Dent Assoc 2003;134(Spec. No.):34S-40S.*<br><br>*Lalla E, Kaplan S, Chang SM, et al. Periodontal infection profiles in type 1 diabetes. J Clin Periodontol 2006;33:855-862.*		

Diabetes duration	Diabetes duration in years			
Gingivitis/periodontitis				

* In the context of a particular study, variables can be demonstrated not to be confounders and so not included in the analysis: (a) if they are not predictive of the outcome; (b) if they are not predictive of exposure; or (c) because adjustment makes no or minimal difference to the estimated effect of the primary parameter. Note that “no statistically significant association” is not the same as “not predictive”.

Preliminary consideration of criteria used to determine the accuracy of measurement of exposure and outcome

Complete a row for each measure listed in the study for the (i) exposure and (ii) outcome. Of the measures listed in the protocol, consider the sensitivity, specificity, and confidence in the methods used in the study.

(ix) Exposure measurement method listed in the study		
Method of measurement	Measured exposure	Is the exposure measured validly and reliably by this method (or these methods)?
Professionally diagnosed	Type DM (I/II)	Patients with a previous (>2 years) and confirmed diagnosis (fasting glucose ‡126 mg/dL or hemoglobin A1c [HbA1c] ‡6.5%) of type I or II diabetes mellitus from the Hospital Universitario San Vicente de Paul (Medellin, Colombia) were invited to participate in the study.<br><br>Values for fasting preprandial glycemia (FPG) (milligrams per deciliter) in all participants and glycosylated hemoglobin (HbA1c percentage) only in individuals with diabetes were recorded. Blood glucose levels were analyzed as follows: normal fasting glucose <100 mg/dL, impaired fasting glucose ‡100 but <126 mg/dL, and hyperglycemia (diabetes) ‡126 mg/dL.

(x) Outcome measurement method listed in the study		
Method of measurement	Measured outcome	Is the outcome measured validly and reliably by this method (or these methods)?
Clinical periodontal examination	PD, CAL, BOP, number of teeth	Performed by two calibrated clinicians in all participants. The recording was calibrated until intraclass and interclass k values between 0.80 and 0.90.<br><br>Periodontal probing performed by using a marked periodontal probe and measurements were rounded to the next millimetre.

Radiographic	Bone loss	To determine possible changes in variables of interest (demographic, biologic and behavioural)

Preliminary consideration of co-exposures

Complete a row for each important co-intervention (i) listed in the review protocol; and (ii) relevant to the setting of this particular study, or which the study authors identified as important.
"Important" co-interventions are those for which, in the context of this study, adjustment is expected to lead to a clinically important change in the estimated effect of the intervention.

**(ix) Co-exposures listed in the review protocol**		
Co-exposure	Is there evidence that controlling for this co-exposure was unnecessary (e.g., because it was not administered)?	Is presence of this co-exposure likely to favor outcomes in the experimental or the control group
		Favor experimental / Favor comparator / No information
		Favor experimental / Favor comparator / No information
		Favor experimental / Favor comparator / No information

**(x) Additional co-exposures relevant to the setting of this particular study, or which the study authors identified as important**		
Co-exposure	Is there evidence that controlling for this co-exposure was unnecessary (e.g., because it was not administered)?	Is presence of this co-exposure likely to favor outcomes in the experimental or the control group
		Favor experimental / Favor comparator / No information
		Favor experimental / Favor comparator / No information
		Favor experimental / Favor comparator / No information

Risk of bias assessment (cohort-type studies)

Bias due to confounding	1.1 **Is there potential for confounding of the effect of exposure in this study? If N or PN to 1.1:** the study can be considered to be at low risk of bias due to confounding and no further signalling questions need be considered	**Y** / PY / PN / N	Yes, due the cross-sectional design.
	**If Y/PY to 1.1, answer 2.1 and 1.3 to determine whether there is a need to assess time-varying confounding:**		
	**1.2. If Y or PY to 1.1: Was the analysis based on splitting follow up time according to exposure received?** **If N or PN to 1.2**, answer questions 1.4 to 1.6, which relate to baseline confounding	NA / Y / PY / PN / **N** / NI	The participants could not switch between exposures, so the outcome could not be biased due time varying confounding. It is a cross-sectional, so measurements were performed ones.
	**1.3. If Y or PY to 1.2: Were exposure discontinuations or switches likely to be related to factors that are prognostic for the outcome?**	NA / **Y** / PY / PN / N / NI	It is possible that there will be undiagnosed diabetics in the non-diabetic (control) group. Only in de DM individuals FPG and HbA1c values were recorded. The controlls were seleected from the school of Dentistry.
	**If N or PN to 1.3**, answer questions 1.4 to 1.6, which relate to baseline confounding		
	**1.4. Did the authors use an appropriate analysis method that adjusted for all the critically important confounding areas?**	NA / **Y** / PY / PN / N / NI	Linear regression analysis and Spearman correlation was used.
	**1.5. If Y or PY to 1.4: Were confounding areas that were adjusted for measured validly and reliably by the variables available in this study?**	NA / Y / PY / PN / N / NI	Yes.
	**1.6. Did the authors avoid adjusting for post-exposure variables?**	NA / Y / PY / PN / N / NI	No information about post-exposure variables.
	**If Y or PY to 1.3**, answer questions 1.7 and 1.8, which relate to time-varying confounding		

	**1.7. Did the authors use an appropriate analysis method that adjusted for all the critically important confounding areas and for time-varying confounding?**	NA / Y / PY / PN / N / NI	Yes.
	**1.8. If Y or PY to 1.7: Were confounding areas that were adjusted for measured validly and reliably by the variables available in this study?**	NA / Y / PY / PN / N / NI	Previous studies showed that the potential confounders possibly tended to bias the outcome.
	**Risk of bias judgement**	**Serious**	**Only a short description about the analysis method to establish the relationship between glycemia and periodontal clinical parameters. Confounding is not specifically mentioned.**
Bias in selection of participants into the study	**2.1. Was selection of participants into the study (or into the analysis) based on variables measured after the start of the exposure?**<br><br>**<u>If N or PN to 2.1 go to 2.4</u>**	**Y** / PY / PN / N / NI	Two groups were selected separately.<br><br>1. DM: patients with a previous and confirmed diagnosis of type ½ diabetes were invited to participate (fasting glucose ‡126 mg/dL or haemoglobin A1c [HbA1c] ‡6.5%)<br><br>2. No-DM: selected from the school of dentistry at the University based on the following inclusion.
	**2.2. <u>If Y/PY to 2.1:</u> Were the post-exposure variables that influenced selection associated with exposure?**	Y / PY / PN / N / **NI**	No information.
	**2.3. <u>If Y/PY to 2.2:</u> Were the post-exposure variables that influenced eligibility selection influenced by the outcome or a cause of the outcome?**	NA/ **Y**/ PY / PN / N / NI	Based on the following criteria:<br>- ≥ 18 years old;<br>- Voluntary participation; confirmed type I or II diabetes mellitus<br>- other controlled systemic diseases (e.g., hypertension);

			- ≥ 10 teeth present.<br><br>Individuals were excluded when they presented any systemic disease that contraindicated the clinical examination, had any previous (3 months) consumption of antibiotics and/or anti-inflammatory drugs, received previous periodontal treatment (6 months), and were pregnant or positive for human immunodeficiency virus.
	**2.4 Do start of follow-up and start of exposure coincide for most participants?**	NA / **Y** / PY / PN / N / NI	The start of the exposure could change between participants, but clinical periodontal procedures were performed ones.
	**2.5 If Y/PY to 2.2 and 2.3, or N/PN to 2.4: Were adjustment techniques used that are likely to correct for the presence of selection biases?**	NA / Y / PY / PN / **N** / NI	No techniques were used that correct fort he presence of selection bias.
	**Risk of bias judgement**	**Moderate**	**Individuals were separately selected. The controls did not receive screening on FPG and HbA1c levels to correct for potential misclassification.**<br><br>**Clearly defined inclusion and exclusion criteria.**
Bias in classification of exposures	**3.1 Is exposure status well defined?**	Y / PY / PN / N / NI	Yes, clearly defined. Patients with a previous and confirmed diagnosis of type I/II were invited in the DM group. Thereafter, values for FPG and HbA1c percentages was recorded (only in the individuals with diabetes).
	**3.2 Did entry into the study begin with start of the exposure?**	Y / PY / PN / N / NI	Some of the participants could been exposed for DM for a longer time than others. The authors measured

			diabetes duration in years for separate DM subgroups.<br>It is possible that there will be undiagnosed diabetics in the non-diabetic (control) group.
	**3.3 Was information used to define exposure status recorded prior to outcome assessment?**	**Y** / PY / PN / N / NI	Yes. First based on confirmed DM diagnosis from a hopsial. Second, based on FPG values and HbA1c levels.
	**3.4 Could classification of exposure status have been affected by knowledge of the outcome or risk of the outcome?**	Y / PY / PN / **N** / NI	No.
	**3.5 Were exposure assessment methods robust (including methods used to input data)?**	**Y** / PY / PN / N / NI	Yes, see above 3.4
	**Risk of bias judgement**	**Moderate**	**Diabetes was properly assessed according professionally diagnosed criteria and FPG/Hba1C levels. There could be DM in the no-DM group (misclassification) because these participants did not receive any examination about DM, only based on previous (>2 years) and confirmed diagnosis.**
Bias due to departures from intended exposures	**4.1. Is there concern that changes in exposure status occurred among participants?**<br><br>**If your aim for this study is to assess the effect of initiating and adhering to an exposure (as in a per-protocol analysis), answer questions 4.2 and 4.3, otherwise continue to 4.4 if Y or PY to 4.1.**	**Y** / PY / PN / N / NI	Yes, this is possible, but they measured diabetes duration in years.
	**4.2. Did many participants switch to other exposures?**	**Y** / PY / PN / N / NI	It is possible that the control of diabetes changes over time.

	4.3. Were the critical co-exposures balanced across exposure groups?	Y / PY / PN / N / NI	Yes, see table 1 (demographic description of the patients included in the study).
	4.4. If NY/PN PY to 4.1, or Y/PY to 4.2, or 4.3: Were adjustment techniques used that are likely to correct for these issues?	NA / Y / PY / PN / **N** / NI	No
	**Risk of bias judgement**	**Moderate**	**There were some deviations from usual practice. No adjustment techniques were used to correct for these issues (for example matching).**
Bias due to missing data	5.1 Were there missing outcome data?	Y / PY / PN / **N** / NI	Missing outcome data during or after analysis were not reported.
	5.2 Were participants excluded due to missing data on exposure status?	Y / PY / PN / **N** / NI	All the participants included for analysis were reported.
	5.3 Were participants excluded due to missing data on other variables needed for the analysis?	Y / PY / PN / N / **NI**	No information.
	5.4 If Y/PY to 5.1, 5.2 or 5.3: Are the proportion of participants and reasons for missing data similar across exposures?	NA / Y / PY / PN / N / NI	No information.
	5.5 If Y/PY to 5.1, 5.2 or 5.3: Were appropriate statistical methods used to account for missing data?	NA / Y / PY / PN / N / NI	Not necessary, because no missing data is reported.
	**Risk of bias judgement**	**Low**	**Data is complete.**<br><br>**In the current review were excluded smokers, because they were separately analysed. However, none of the DM reported smoking. Therefore, only the non-**

			**smoking no DM were considered as control group. The controls consisted thereafter of 59 individuals.**
Bias in measurement of outcomes	**6.1 Could the outcome measure have been influenced by knowledge of the exposure received?**	Y / PY / PN / **N** / NI	The clinically periodontal examination was performed by two calibrated clinicians. Periodontal probing was performed using a marked periodontal probe.<br><br>The recording was calibrated until intraclass and interclass k values between 0.80 and 0.90.<br><br>Periodontal clinical parameters were recorded in six sites around each tooth excluding wisdom teeth.
	**6.2 Was the outcome measure sensitive?**	Y / PY / PN / N / NI	Specifically, the outcome tooth loss is<br>is a surrogate/hard endpoint and not sensitive (for change).<br><br>The other outcome measurements were sensitive, but were not considered in the current review.
	**6.3 Were outcome assessors unaware of the exposure received by study participants?**	Y / PY / PN / N / **NI**	No information.
	**6.4 Were the methods of outcome assessment comparable across exposure groups?**	Y / PY / PN / N / NI	Yes, outcome assessment were comparable across the two groups.
	**6.5 Were any systematic errors in measurement of the outcome unrelated to exposure received?**	Y / PY / PN / N / NI	Unlikely, see 6.2.
	**Risk of bias judgement**	**Low**	**The methods of outcome assessment were comparable across exposure groups.**

Bias in selection of	Is the reported effect estimate likely to be selected, on the basis of the results, from...?		
the reported result	**7.1. ... multiple outcome *measurements* within the outcome domain?**	Y / PY / PN / **N** / NI	The authors reported the median instead of mean number of teeth, so the authors were contacted to receive additional information.
	**7.2 ... multiple *analyses* of the exposure-outcome relationship?**	**Y** / PY / PN / N / NI	Fully reported
	**7.3 ... different *subgroups*?**	**Y** / PY / PN / N / NI	Fully reported.
	**Risk of bias judgement**	**Low**	**Fully reported after contacting the authors for additional information about the mean number of teeth**
Overall bias	**Risk of bias judgement**	**Serious**	**The authors well performed the clinical examination by two calibrated clinicians and intraclass and interclass values were calculated. However, the authors did not appropriate for al important confounding areas. Correlation and regression analysis were carried out, but not further described. Thereafter, the method for exposure assessment were not comparable across the two groups.**

Online Appendix S2-7

Preliminary tool for risk of bias in exposure study: Sensorn et al. 2012

Title: Relationship between Diabetes Mellitus and Tooth Loss in Adults Residing in Ubonratchathani Province, Thailand

Year: 2013

Authors: Watcharaporn Sensorn DDS(a)Supaporn Chatrchaiwiwatana PhD(b), Sauwanan Bumrerraj MD(c)

A: Nachaluay Hospital, Ubonratchathani, Thailand

B: Faculty of Dentistry, Khon Kaen University, Khon Kaen, Thailand

C: Faculty of Medicine, Khon Kaen University, Khon Kaen, Thailand

PMID: 23390792

***AIM**: the purpose of the present cross- sectional study was to determine the association between tooth loss and diabetes mellitus adjusting for potential confounding factors in adults residing in Nachaluay district, Ubonratchathani province, Thailand.*

Specify a target experiment specific to the study:

☐ The protocol-specified target experiment fully applies ***OR***

Participant

Experimental exposure

Control exposure

Participant:
The population consisted of diabetic and non-diabetic adults living in Nachaluay district, Ubonratchathani province, Thailand during the year 2010. The samples included 605 people (130 males and 475 females). Their ages ranged from 20 to 86 years.

Experimental outcome:
379 diabetics: fasting plasma glucose (FPG) value ≥ 126 mg/dl and had already been informed of their condition by a medical practitioner.

Control exposure:
226 non-diabetic

Specify the outcome

Specify which outcome is being assessed for risk of bias (typically from among those earmarked for the Summary of Findings table). Specify whether this is a proposed benefit or harm of exposure.

Tooth loss

Is your aim for this study…?

■ to assess the effect of initiating intervention (as in an intention-to-treat analysis)

☐ to assess the effect of initiating and adhering to intervention (as in a per-protocol analysis)

☐ other (specify)

Specify the numerical result being assessed

In case of multiple alternative analyses being presented, specify the numeric result (e.g. RR = 1.52 (95% CI 0.83 to 2.77) and/or a reference (e.g. to a table, figure or paragraph) that uniquely defines the result being assessed.

Results were obtained by means of descriptive, bivariate, and multivariable logistic regression. Descriptive statistics using mean, standard deviation and proportion were used to analyse the basic

information such as age, sex, marital status, education level, main occupation, income and oral health status, both periodontal diseases and dental caries.

Bivariate statistics using chi-square test and independent t-test were employed, based on the assumptions of the statistics being used, to assess the preliminary relationship between tooth loss (defined as missing ≥ 1 teeth) and potential predictors, not yet adjusting for confounding factors.

The final multivariable logistic regression model having tooth loss as an outcome was achieved to define a set of variables related to tooth loss. The adjusted odds ratios along with their 95% CIs were reported and p-value of less than 0.05 was considered statistically significant.

Preliminary consideration of confounders

Complete a row for each important confounding area (i) listed in the review protocol; and (ii) relevant to the setting of this particular study, or which the study authors identified as potentially important. *"Important" confounding areas are those for which, in the context of this study, adjustment is expected to lead to a clinically important change in the estimated effect of the exposure. "Validity" refers to whether the confounding variable or variables fully measure the area, while "reliability" refers to the precision of the measurement (more measurement error means less reliability).*

**(xi) Confounding areas listed in the review protocol**				
Confounding area	Measured variable(s)	Is there evidence that controlling for this variable was unnecessary? *	Is the confounding area measured validly and reliably by this variable (or these variables)?	OPTIONAL: Is adjusting for this variable (alone) expected to move the effect estimate up or down?
Demographic factors	Gender, age	No	Yes: Gender (male/female), age (year).	No information
BMI, dietary	Weight, height, BMI	No	Yes: weight (kilogram), height (meter), waist (inch), body mass index (kg/m^2)	No information

Education level	Education level, diploma/bachelor's degree	No	Education level (no schooling/lower primary (prathom 4)/lower primary (prathom 6)/lower secondary/upper secondary or vocational school/bachelor diploma/bachelor degree/ others), occupation (unemployed/labor/agricultural/ civil/merchant/others.	No information
Social/economic status	Marital status, income level	No	Yes: marital status (single/married/divorced/ widowed), income (monthly/yearly).	No information
General health status	DM, other diseases, duration of DM, level of fasting blood sugar, treatment of DM, xerostomia, diet control, exercise, food intake inducing DM	No	Having diabetes mellitus and/or other diseases (yes/ no), duration of having diabetes mellitus (year), level of fasting blood sugar during the past three months (mg/dl), treatment of diabetes (yes/no), xerostomia (yes/no), diet control (yes/no), exercise (none/sometime/ regularly), food intake inducing diabetes mellitus	No information
Smoking, alcohol use	Smoking and alcohol use.	No	Alcohol use, smoking and betel chewing (non-user/ex-user/occasional user/ regular user), duration of alcohol use, tobacco smoking and betel chewing (year),	No information

(xii)	**Additional confounding areas relevant to the setting of this particular study, or which the study authors identified as important**

Confounding area	Measured variable(s)	Is there evidence that controlling for this variable was unnecessary? *	Is the confounding area measured validly and reliably by this variable (or these variables)?	OPTIONAL: Is adjusting for this variable (alone) expected to move the effect estimate up or down?
Dental hygiene status	Tooth brushing, oral cleansing aids, denture wearing, knowledge about relationship DM and tooth loss.	No.	Tooth brushing (none/sometimes/once daily/ twice a day/more than twice a day), oral cleansing aids other than brushing, oral care problem, history of tooth loss, denture wearing and cleaning as well as knowledge and attitude about relationship between diabetes mellitus and tooth loss (yes/no).	No information

* In the context of a particular study, variables can be demonstrated not to be confounders and so not included in the analysis: (a) if they are not predictive of the outcome; (b) if they are not predictive of exposure; or (c) because adjustment makes no or minimal difference to the estimated effect of the primary parameter. Note that “no statistically significant association” is not the same as “not predictive”.

Preliminary consideration of criteria used to determine the accuracy of measurement of exposure and outcome

Complete a row for each measure listed in the study for the (i) exposure and (ii) outcome. Of the measures listed in the protocol, consider the sensitivity, specificity, and confidence in the methods used in the study.

(xi) Exposure measurement method listed in the study		
Method of measurement	Measured exposure	Is the exposure measured validly and reliably by this method (or these methods)?
Professionally diagnosed (FPG)	Diabetes mellitus	Yes:<br>“known diabetes”, who had fasting plasma glucose (FPG) value ≥ 126 mg/dl and had already been informed of their condition by a medical practitioner.<br><br>The patients were followed-up routinely by a medical team. The check-up periods for these diabetic patients were scheduled every one, two, or three months, for fasting plasma glucose (FPG) level > 180 mg/dl, > 130-180 mg/dl, or 80-130 mg/dl,

		respectively.<br><br>Discussion session: The finding that diabetes status (defined as yes vs. no) and duration of diabetes demonstrated some relations with tooth loss while FPG did not show any trend of association might reflect that the validity of measurement of FPG might not be adequate in the present study. Under limited resources, the low-cost FPG is practically used to measure diabetes in rural hospitals of Thailand. However, it should be noted that haemoglobin A1c is considered a more efficient measurement and should be increasingly used instead.
Interview	Diabetes mellitus	Having diabetes mellitus and/or other diseases (yes/ no), duration of having diabetes mellitus (year), level of fasting blood sugar during the past three months (mg/dl), treatment of diabetes (yes/no).

(xii) Outcome measurement method listed in the study		
Method of measurement	Measured outcome	Is the outcome measured validly and reliably by this method (or these methods)?
Oral examination (dentist)	Dental caries status	Yes, based on WHO criteria.<br><br>Dental caries status was measured using decayed, missing and filled teeth (DMFT) index whereby the criteria were coded as follows: 0 = normal tooth without caries (sound tooth), 1 = decayed, 2 = filled with decayed, 3 = filled with no decayed, 4 = missing due to caries, 5 = missing due to other reasons, 6 = fissure sealant, 7 = crown or bridge abutment, 8 = unseen in the oral cavity, 9 = fracture, 10 = abrasion or erosion, and 11 = status of teeth not included in the above.<br><br>Treatment need was coded as 0 = no need for treatment, 1 = prevention of caries, 2 = fissure sealant, 3 = one surface filling, 4 = two or more surface filling, 5 = crown, 6 = veneer, 7 = root canal treatment, 8 = extraction, 9 = need for other care (specify type of treatment).
Oral examination (dentist)	Periodontal conditions	Yes, based on WHO criteria.<br><br>Periodontal conditions were determined for periodontal pocket as well as clinical attachment loss (CAL) using WHO periodontal probe. The Community Periodontal Index (CPI) was used based on the following criteria: 0 = healthy gingiva, 1 =

		bleeding gingiva, 2 = calculus, 3 = calculus with bleeding, 4 = pocket 4-5 mm, 5 = pocket 6 mm or more, 9 = cannot be determined, 10 = missing sextant/excluded.
Oral examination (dentist)	Debris	Yes, based on WHO criteria.<br><br>Debris Index Simplified (DI-S) was used to measure deposition of food debris based on the following criteria: 0 = no debris, 1 = debris deposit 1/3 of tooth surface, 2 = debris deposit 1/3-2/3 of tooth surface, 3 = debris deposit > 2/3 of tooth surface.

Preliminary consideration of co-exposures

Complete a row for each important co-intervention (i) listed in the review protocol; and (ii) relevant to the setting of this particular study, or which the study authors identified as important.
"Important" co-interventions are those for which, in the context of this study, adjustment is expected to lead to a clinically important change in the estimated effect of the intervention.

**(xi)** Co-exposures listed in the review protocol		
Co-exposure	Is there evidence that controlling for this co-exposure was unnecessary (e.g., because it was not administered)?	Is presence of this co-exposure likely to favor outcomes in the experimental or the control group
-	-	No information

**(xii)** Additional co-exposures relevant to the setting of this particular study, or which the study authors identified as important		
Co-exposure	Is there evidence that controlling for this co-exposure was unnecessary (e.g., because it was not administered)?	Is presence of this co-exposure likely to favor outcomes in the experimental or the control group
-	-	No information

Risk of bias assessment (cohort-type studies)

Bias due to confounding	1.1 **Is there potential for confounding of the effect of exposure in this study? If N or PN to 1.1:** the study can be considered to be at low risk of bias due to confounding and no further signalling questions need be considered	**Y** / PY / PN / N	Yes. Potential thoughts for confounding the effect of exposure in the study due the cross-sectional design.<br><br>Thereby the baseline oral health conditions of the participants were possible not the same at baseline.<br><br>For example:<br>the risk of tooth loss may be greater in patients who have already poor periodontal status before beginning of the study.
	**If Y/PY to 1.1, answer 2.1 and 1.3 to determine whether there is a need to assess time-varying confounding:**		
	**1.2. If Y or PY to 1.1: Was the analysis based on splitting, follow up time according to exposure received?**<br><br>**If N or PN to 1.2**, answer questions 1.4 to 1.6, which relate to baseline confounding	NA / Y / PY / PN / **N** / NI	No, the participants could not switch between exposures, so the outcome could not be biased due time varying confounding.
	**1.3. If Y or PY to 1.2: Were exposure discontinuations or switches likely to be related to factors that are prognostic for the outcome?**	NA / **Y** / PY / PN / N / NI	Yes, it is possible that there will be undiagnosed diabetics in the non-diabetic (control) group due of misclassification.
	**If N or PN to 1.3**, answer questions 1.4 to 1.6, which relate to baseline confounding		
	**1.4. Did the authors use an appropriate analysis method that adjusted for all the critically important confounding areas?**	NA / **Y** / PY / PN / N / NI	The authors adjusted for several confounders using multivariable logistic regression models.
	**1.5. If Y or PY to 1.4: Were confounding areas that were adjusted for measured validly and reliably by the variables available in this study?**	NA / Y / PY / PN / N / NI	Described in the results and table 3 (bivariate relationship between tooth loss and selected variables split out in DM and no DM). These descriptive

			statistics are also adjusted in other reliable and comparable studies.<br><br>The authors used multivariable logistic regression models.
	**1.6. Did the authors avoid adjusting for post-exposure variables?**	NA / Y / PY / PN / N / NI	The authors did not adjust for post-exposure variables.
	**If Y or PY to 1.3**, answer questions 1.7 and 1.8, which relate to time-varying confounding		
	**1.7. Did the authors use an appropriate analysis method that adjusted for all the critically important confounding areas and for time-varying confounding?**	NA / Y / PY / PN / N / NI	Yes, see 1.4
	**1.8. If Y or PY to 1.7: Were confounding areas that were adjusted for measured validly and reliably by the variables available in this study?**	NA / Y / PY / PN / N / NI	Yes, see 1.5
	**Risk of bias judgement**	**Low**	**Confounding expected, all known critically important confounding domains appropriately measured and adjusted (multivariable logistic regression models).**
Bias in selection of participants into the study	**2.1. Was selection of participants into the study (or into the analysis) based on variables measured after the start of the exposure?**<br><br>**If N or PN to 2.1 go to 2.4**	Y / PY / PN / N / **NI**	The population consisted of diabetic and non-diabetic adults living in Nachaluay district, Ubonratchathani province, Thailand during the year 2010. The samples included 605 people (130 males and 475 females; 379 diabetics and 226 non-diabetic).<br><br>No specific information about the criteria for inclusion and participating of the study.

	**2.2. If Y/PY to 2.1: Were the post-exposure variables that influenced selection associated with exposure?**	Y / PY / PN / N / NI	Yes, it is possible that the severity of diabetes changes (over time) and within the exposure group.<br><br>Because of the cross-sectional design, there were no detailed information about previous glycemic control. Only one measurement at 1 point is described and used to define the DM and no DM group.
	**2.3. If Y/PY to 2.2: Were the post-exposure variables that influenced eligibility selection influenced by the outcome or a cause of the outcome?**	NA / Y / PY / PN / N / NI	No information if participants were excluded in case of missing data (on covariates.)
	**2.4 Do start of follow-up and start of exposure coincide for most participants?**	NA / Y / PY / PN / N / NI	No, the start of exposure already exists prior of the study. Thereby the severity changes between participants.
	**2.5 If Y/PY to 2.2 and 2.3, or N/PN to 2.4: Were adjustment techniques used that are likely to correct for the presence of selection biases?**	NA / Y / PY / PN / N / **NI**	No information about adjustment techniques.
	**Risk of bias judgement**	**Serious**	**Selection of the participants is described too briefly in the material and methods section. The authors had could described the inclusion and exclusion criteria more broadly, for example exclusion by other systematic diseases.**<br><br>**Thereby it is unknown if the sample is representative for the whole population in Thailand. A detailed description about the province in question is not available.**<br><br>**On the other hand, a strength of the present study is de sample**

			**size. The sample was big enough to give sufficient statistical power of the study greater than 80% (described by the authors in the discussion part).**
Bias in classification of exposures	**3.1 Is exposure status well defined?**	**Y** / PY / PN / N / NI	Yes: diabetic patients were classified as "known diabetes", who had fasting plasma glucose (FPG) value ≥ 126 mg/dl and had already been informed of their condition by a medical practitioner.<br><br>These patients were followed-up routinely by a medical team.
	**3.2 Did entry into the study begin with start of the exposure?**	Y / PY / PN / N / NI	Yes, the whole diabetic group did entry into the study begin with the start of exposure due the cross-sectional design.
	**3.3 Was information used to define exposure status recorded prior to outcome assessment?**	Y / PY / PN / N / NI	Yes, based on fasting plasma glucose (FPG) value.
	**3.4 Could classification of exposure status have been affected by knowledge of the outcome or risk of the outcome?**	Y / PY / PN / N / **NI**	It is unknown if the exposure status has been affected by knowledge of the outcome or risk of outcome, but not likely.<br><br>The data collection is done separately by two different operators:<br>1. Interview: baseline characteristics<br>2. Oral examination: periodontal conditions and dental caries status.

	**3.5 Were exposure assessment methods robust (including methods used to input data)?**	Y / PY / PN / **N** / NI	No: the finding that diabetes status (defined as yes vs. no) and duration of diabetes demonstrated some relations with tooth loss (questioned in the interview on baseline characteristics), while FPG did not show any trend of association might reflect that the validity of measurement of FPG might not be adequate in the present study.
	**Risk of bias judgement**	**Serious**	**Afterwards it turns out and described in the discussion session: it should be noted that hemoglobin A1c is considered a more efficient measurement and should be increasingly used instead fasting plasma glucose values.**
Bias due to departures from intended exposures	**4.1. Is there concern that changes in exposure status occurred among participants?**<br><br>**If your aim for this study is to assess the effect of initiating and adhering to an exposure (as in a per-protocol analysis), answer questions 4.2 and 4.3, otherwise continue to 4.4 if Y or PY to 4.1.**	Y / PY / PN / N / NI	Undiagnosed diabetics might be classified as a non-diabetic group, because the exposure status is diagnosed previously at the start based on FGG value.<br>Misclassification is limited because the patients were followed up routinely by a medical team.<br>The check-up periods for these diabetic patients were schedule every, two, or three months, for fasting plasma glucose (FPG) level > 180 mg/dl, > 130-180 mg/dl, or 80-130 mg/dl, respectively.

			Thereby no distinction is made in severity or type of diabetes.
	**4.2. Did many participants switch to other exposures?**	Y / PY / PN / N / **NI**	Undiagnosed diabetics might be classified as a non-diabetic group.
	**4.3. Were the critical co-exposures balanced across exposure groups?**	Y / PY / PN / N / NI	A detailed description about co-exposures is specifically made for the DM/no-DM group in table 3.<br><br>The mean age for DM group is higher, had a higher waist rate and lower income and education levels.
	**4.4. If NY/PN PY to 4.1, or Y/PY to 4.2, or 4.3: Were adjustment techniques used that are likely to correct for these issues?**	NA / Y / PY / PN / N / NI	No
	**Risk of bias judgement**	**Moderate**	**The authors-based DM status on FPG measurements instead of the most commonly used HbA1c levels. This might be not adequate. It is used because of the low-cost of FPG.**
Bias due to missing data	**5.1 Were there missing outcome data?**	Y / PY / PN / N / **NI**	No information about missing data. It is likely that only participant who underwent the full interview and received the whole oral examination were included for study analysis.<br><br>Thereby all the 605 included people were used for data analysis, so no indication for missing data.
	**5.2 Were participants excluded due to missing data on exposure status?**	Y / PY / PN / **N** / NI	All the participants underwent an FPG test to compare fasting plasma glucose values.
	**5.3 Were participants excluded due to missing data on other variables needed for the analysis?**	Y / PY / PN / N / **NI**	No specific information, but not necessary? Only participants of which data is complete were included for analysis.

	**5.4 <u>If Y/PY to 5.1, 5.2 or 5.3:</u> Are the proportion of participants and reasons for missing data similar across exposures?**	**NA** / Y / PY / PN / N / **NI**	No specific information about missing data.
	**5.5 <u>If Y/PY to 5.1, 5.2 or 5.3:</u> Were appropriate statistical methods used to account for missing data?**	**NA** / Y / PY / PN / N / NI	Not necessary?
	**Risk of bias judgement**	**Low**	**There is no specific information about missing data, but it is likely that all the participants who underwent oral examination and interview on baseline characteristics were included in the study.**<br><br>**Data were reasonably complete.**
Bias in measurement of outcomes	**6.1 Could the outcome measure have been influenced by knowledge of the exposure received?**	Y / PY / PN / N / NI	It is possible, but the oral examination and interview about baseline characteristics is done separately by two different operators:<br>- Oral examination: dentist<br>- Interview: research team
	**6.2 Was the outcome measure sensitive?**	Y / PY / PN / N / NI	No, the outcome measurement is a surrogate/hard endpoint and not sensitive (for change).
	**6.3 Were outcome assessors unaware of the exposure received by study participants?**	Y / PY / PN / N / NI	Yes, likely.
	**6.4 Were the methods of outcome assessment comparable across exposure groups?**	Y / PY / PN / N / NI	Yes, both groups received the same interview and oral examination.
	**6.5 Were any systematic errors in measurement of the outcome unrelated to exposure received?**	**Y** / PY / PN / N / NI	Yes:<br><br>The dentist who conducted the examination had previously been trained for assessing oral health indices validly and had acceptable kappa values for repeatability of at least 80%. To ensure consistency in

			performing the oral examination in the field, repeated measurements in 10% of the samples were done and the kappa statistics throughout the whole examination were higher than 80%. The research team who conducted the interview also had been well-trained to do it. To reduce errors in the process of data entry, double data entry was done independently by two well-trained research staff.
	**Risk of bias judgement**	**Low**	**The methods of outcome assessment were comparable across both groups.**
Bias in selection of	Is the reported effect estimate likely to be selected, on the basis of the results, from...?		
the reported result	**7.1. ... multiple outcome *measurements* within the outcome domain?**	**Y** / PY / PN / N / NI	All the results about the clinical parameters were reported.
	**7.2 ... multiple *analyses* of the exposure-outcome relationship?**	Y / PY / PN / N / NI	All the multiple analyses were reported.
	**7.3 ... different *subgroups*?**	Y / PY / PN / N / NI	No further distinction in subgroups were made, only in DM and no DM.
	**Risk of bias judgement**	**Low**	**All the results have been fully reported.**
	Optional: What is the predicted direction of bias due to selection of the reported result?	Favours the best possible outcome for the authors	If there were any concern about publication bias; the predicted direction of bias is favouring the best possible outcome for the authors.
Overall bias	**Risk of bias judgement**	**Serious**	**The authors adjusted for several confounding areas and adjusted these sources by using multivariable logistic regression. Thereby all the results have been**

			fully reported.<br><br>Despite this, we have become to a serious risk of bias judgement due the assessment of DM based on FPG values and the limited information about selection of the participants in the method section.<br><br>The present results agree well with several prior studies around the world and can be established as baseline knowledge for future research, such as this systematic review.

Online Appendix S2-8

Preliminary tool for risk of bias in exposure study: Kaur et al. 2009

Title: Association between type 1 and type 2 diabetes with periodontal disease and tooth loss

Year: 2009

Authors: Kaur G, Holtfreter B, Rathmann W, Schwahn C, Wallaschofski H, Schipf S, Nauck M, Kocher T.

PMID: 19622096

AIM: to determine whether both type 1 (T1DM) and type 2 diabetes mellitus (T2DM) are associated with increased prevalence and extent of periodontal disease and tooth loss compared with non-diabetic subjects within a homogeneous adult study population.

Specify a target experiment specific to the study:

☐	The protocol-specified target experiment fully applies	***OR***	Participant	**Participant**:<br>- General population from the SHIP Trend study (population based survey in North-Eastern Germany). This is an representative sample 7008 subjects aged between 20-79 years was selected from the population.<br>- General population from the Centre of Cardiology and Diabetes, Karlsburg.
			Experimental exposure	**Experimental exposure:**<br>The T1DM cohort (233 subjects aged 20–81 years) was recruited from the Centre of Cardiology and Diabetes, Karlsburg, and the surrounding practicing diabetologists. These subjects lived in the same geographical region as the subjects recruited for SHIP<br><br>The T2DM cohort (229 subjects) was recruited from the SHIP Trend study.
			Control exposure	**Control exposure:**<br>Nondiabetic subjects from the SHIP Trend study.

General population from the SHIP Trend study (population-based survey in North-Eastern Germany). Entire regional population: 212.157 inhabitants → a representative sample 7008 subjects aged between 20-79 years was selected from the population registration offices. A two-way cluster sampling method is used:

- Adopted from the World Health Organization Monitoring Trends and Determinants in Cardiovascular Disease (MONICA) Study; yielding 12 5-year age strata (20–79 years) for both genders, each including 292 individuals. Between October 1997 and May 2001, a total of 4310 individuals (response 68.8%) participated in the study.
- The T1DM cohort (233 subjects aged 20–81 years) was recruited from the Centre of Cardiology and Diabetes, Karlsburg, and the surrounding practicing diabetologists. These subjects lived in the same geographical region as the subjects recruited for SHIP. Data collection performed between december 1997 and december 2000 from the diabetic registries of the Centre of Cardiology and Diabetes, Karlsburg.

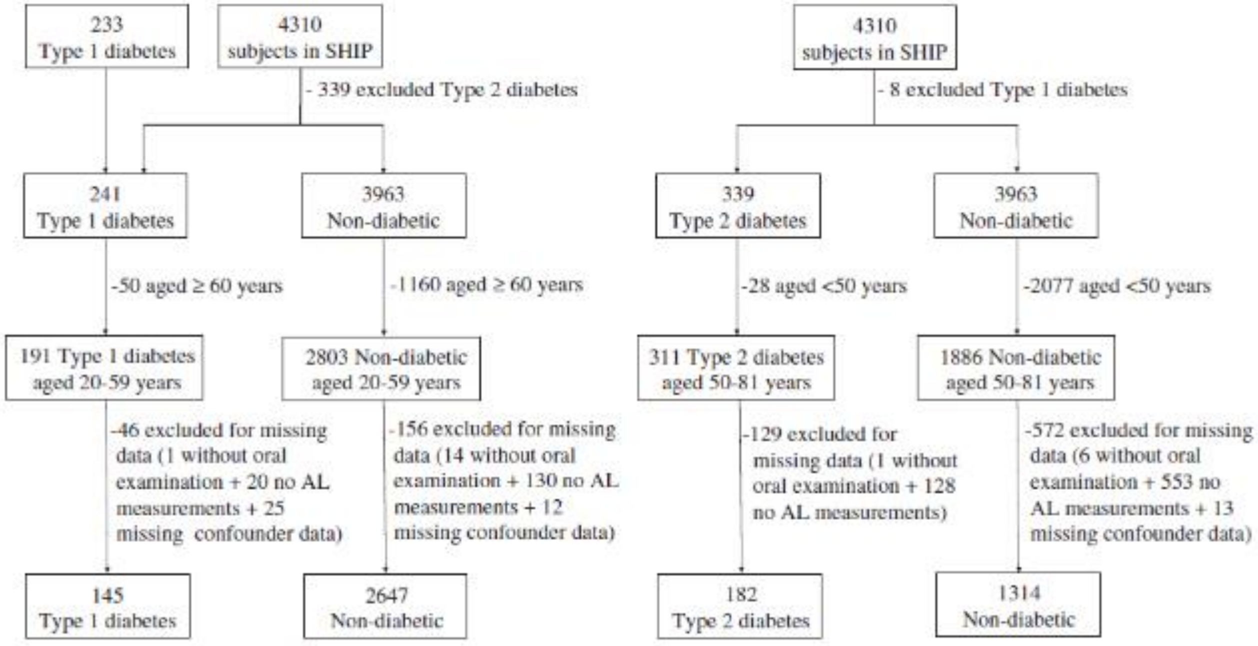

Fig. 1. Description of the study population. AL, attachment loss.

Specify the outcome

Specify which outcome is being assessed for risk of bias (typically from among those earmarked for the Summary of Findings table). Specify whether this is a proposed benefit or harm of exposure.

Periodontal disease is assessed by attachment loss (AL) and the number of missing teeth.<br><br>Worse periodontal health, so more attachment loss or missing fewer teeth can be seen as a harmful effect.

Is your aim for this study…?

■ **to assess the effect of initiating intervention (as in an intention-to-treat analysis)**

□ to assess the effect of initiating and adhering to intervention (as in a per-protocol analysis)

□ other (specify)

Specify the numerical result being assessed

In case of multiple alternative analyses being presented, specify the numeric result (e.g. RR = 1.52 (95% CI 0.83 to 2.77) and/or a reference (e.g. to a table, figure or paragraph) that uniquely defines the result being assessed.

Continuous data were expressed as mean and standard deviation. Nominal data were presented as absolute numbers and per cent values. For continuous data<br>comparisons between groups were performed using the Mann–Whitney U-test.<br>For nominal data, the w2 test was applied.<br><br>Linear regression models were fitted to assess the association between T1DM as well as T2DM and mean AL as the dependent variable. The final model was<br>adjusted for age, gender, school education, smoking, WC and the frequency of dental visits (in the last 12 months).

Linear regression coefficients (B) with their 95% confidence intervals (95% CI) and p values were reported.<br><br>**To evaluate the association between T1DM or T2DM and the number of missing teeth multivariable logistic regression analyses were performed. Because of a bimodal and skewed distribution of number of missing teeth, the variable was dichotomized. Cases with a high number of missing teeth were assessed in relation to their age and gender. Thus, 25% of females and males (separately) with the highest number of missing teeth in each 5-year age group were considered as cases. The reference group included the remaining 75% of females and males (separately) within each 5-year age group. This dichotomous variable was used to estimate the association between both types of diabetes and a high number of missing teeth. Odds ratios (OR) with 95% CI and p values are listed in the tables.**<br><br>Effect modifications were assessed including interaction terms between confounders and the exposure variable in the multivariable models. The statistical significance of interactions was assessed using likelihood ratio tests. In case of a statistically significant interaction (po0.1<br>for interaction), stratified analyses were run.<br><br>Sensitivity analyses were run to assess the association between T1DM, T2DM and periodontal disease by changing disease definition to verify the stability of findings regarding the association between both diabetes types and periodontitis.<br><br>Additionally, analyses were restricted to subjects with at least 12 sites with valid AL measurements. A value of po0.05 was considered to be statistically significant for all analyses

Preliminary consideration of confounders

Complete a row for each important confounding area (i) listed in the review protocol; and (ii) relevant to the setting of this particular study, or which the study authors identified as potentially important. *"Important" confounding areas are those for which, in the context of this study, adjustment is expected to lead to a clinically important change in the estimated effect of the exposure. "Validity" refers to whether the confounding variable or variables fully measure the area, while "reliability" refers to the precision of the measurement (more measurement error means less reliability).*

**(xiii) Confounding areas listed in the review protocol**

Confounding area	Measured variable(s)	Is there evidence that controlling for this variable was unnecessary?*	Is the confounding area measured validly and reliably by this variable (or these variables)?	OPTIONAL: Is adjusting for this variable (alone) expected to move the effect estimate up or down?
Behavioural	Cigarette smoking	No	A computer-aided personal interview.<br>Measured in never, former and current smoker.	
Dental History	Frequency of dental visits		A computer-aided personal interview	
Sociodemographic characteristics			A computer-aided personal interview	
Medical history	BMI (height and weight), Hba1c, white blood cell count, waist circumference		Hb1ac measured in <6, 6-6.9, and >7 in %.<br>White blood cell count in Gpt/l<br>Duration of diabetes in years<br>Age at diabetes diagnosis (years)	

(xiv) Additional confounding areas relevant to the setting of this particular study, or which the study authors identified as important

Confounding area	Measured variable(s)	Is there evidence that controlling for this variable was unnecessary?*	Is the confounding area measured validly and reliably by this variable (or these variables)?	OPTIONAL: Is adjusting for this variable (alone) expected to move the effect estimate up or down?
School education			No information<br>Measured in < 10 years, 10 years and > 10 years.	Favor intervention / Favor control / No information

* In the context of a particular study, variables can be demonstrated not to be confounders and so not included in the analysis: (a) if they are not predictive of the outcome; (b) if they are not predictive of exposure; or (c) because adjustment makes no or minimal difference to the estimated effect of the primary parameter. Note that “no statistically significant association” is not the same as “not predictive”.

Preliminary consideration of criteria used to determine the accuracy of measurement of exposure and outcome

Complete a row for each measure listed in the study for the (i) exposure and (ii) outcome. Of the measures listed in the protocol, consider the sensitivity, specificity, and confidence in the methods used in the study.

(xiii) Exposure measurement method listed in the study		
Method of measurement	Measured exposure	Is the exposure measured validly and reliably by this method (or these methods)?
Physician	Diabetes (T1DM cohort)	The T1DM cohort (233 subjects aged 20–81 years) was recruited from the Centre of Cardiology and Diabetes.
Self-reported physician	Diabetes T1DM (SHIP cohort)	Subjects were defined as T1DM if the onset of disease was before the age of 30 years or if administration of insulin started less than one year after the onset of the disease.
Self-administered questionnaire, diet recommendations or oral anti-diabetic drugs.	Diabetes T2DM (SHIP cohort)	Subjects were defined as having T2DM if the onset of disease was after the age of 29 or if the administration of insulin started 41 year after disease onset in subjects younger than 30 years.<br><br>According to the ATC codes.
Health related interviews and risk-related questionnaires: self-reported physician	Medication use (anti-diabetic drugs)	prescriptions or medications brought during health-related interviews were categorized according to the Anatomical Therapeutic Chemical (ATC) classification system.
Health related interviews and risk-related questionnaires. self-reports	Diabetes duration	Diabetes duration, and duration and mode of anti-diabetic therapy were assessed by self-reports.

(xiv) Outcome measurement method listed in the study

Method of measurement	Measured outcome	Is the outcome measured validly and reliably by this method (or these methods)?
Oral and medical examinations	Periodontal status (AL + PD) in mm, percentage of site with AL/PD >4mm (%)	Yes: registered according to the half-mouth method on the right or the left side in alternate subjects using a periodontal probe (PCP 11, Hu-Friedy, Chicago, IL, USA) at four sites per tooth (mesiobuccal, midbuccal, distobuccal and midlingual) (Hensel et al.2003). Periodontal assessment included attachment loss (AL) and probing depth (PD) measurements.
Oral examination	Periodontal status (number of teeth)	Yes: determined full mouth on a maximum of 28 teeth. All fully erupted teeth, except third molars.<br><br>Calibrated licensed dentists performed all the examinations. Every 6–12 months, calibration exercises were performed on a subset of persons not connected to the study.

Preliminary consideration of co-exposures

Complete a row for each important co-intervention (i) listed in the review protocol; and (ii) relevant to the setting of this particular study, or which the study authors identified as important.
"Important" co-interventions are those for which, in the context of this study, adjustment is expected to lead to a clinically important change in the estimated effect of the intervention.

**(xiii) Co-exposures listed in the review protocol**		
Co-exposure	Is there evidence that controlling for this co-exposure was unnecessary (e.g., because it was not administered)?	Is presence of this co-exposure likely to favor outcomes in the experimental or the control group
		Favor experimental / Favor comparator / No information
		Favor experimental / Favor comparator / No information
		Favor experimental / Favor comparator / No information

**(xiv) Additional co-exposures relevant to the setting of this particular study, or which the study authors identified as important**		
Co-exposure	Is there evidence that controlling for this co-exposure was unnecessary (e.g., because it was not administered)?	Is presence of this co-exposure likely to favor outcomes in the experimental or the control group

		Favor experimental / Favor comparator / No information
		Favor experimental / Favor comparator / No information
		Favor experimental / Favor comparator / No information

Risk of bias assessment (cohort-type studies)

Bias due to confounding	**1.1 Is there potential for confounding of the effect of exposure in this study? If N or PN to 1.1:** the study can be considered to be at low risk of bias due to confounding and no further signalling questions need be considered	**Y** / PY / PN / N	Yes, there is potential for confounding of the effect of exposure in this study. There could be a reason for considerable thoughts that prognostic factors also predict the exposure which is received.<br>.
	**If Y/PY to 1.1, answer 2.1 and 1.3 to determine whether there is a need to assess time-varying confounding:**		
	**1.2. If Y or PY to 1.1: Was the analysis based on splitting, follow up time according to exposure received?**<br>**If N or PN to 1.2**, answer questions 1.4 to 1.6, which relate to baseline confounding	NA / Y / PY / PN / **N** / NI	The participants could not switch between exposures, so the outcome could not be biased due time varying confounding.
	**1.3. If Y or PY to 1.2: Were exposure discontinuations or switches likely to be related to factors that are prognostic for the outcome?**	NA / **Y** / PY / PN / N / NI	Exposure switched are related to the outcome.<br><br>There is a distinction made in type of DM (type I/II) not on severity/control of DM. Uncontrolled DM could lead to a worse prognosis for tooth loss.
	**If N or PN to 1.3**, answer questions 1.4 to 1.6, which relate to baseline confounding		
	**1.4. Did the authors use an appropriate analysis method that adjusted for all the critically important confounding areas?**	NA / **Y** / PY / PN / N / NI	The final model was adjusted for age, gender, school education, smoking, WC and the frequency of dental visits (in the last 12 months).<br><br>Effect modifications were assessed including interaction terms between confounders and the exposure variable in the multivariable models.

			Sensitivity analyses were run to assess the association between T1DM, T2DM and periodontal disease by changing disease definition to verify the stability of findings regarding the association between both diabetes types and periodontitis.
	**1.5. If Y or PY to 1.4: Were confounding areas that were adjusted for measured validly and reliably by the variables available in this study?**	NA / **Y** / PY / PN / N / NI	T1DM and T2DM occur at different age groups. The analyses were performed in different age groups. The study enabled a valid evaluation
	**1.6. Did the authors avoid adjusting for post-exposure variables?**	NA / Y / PY / PN / N / **NI**	No information given about post exposure variables.
	**If Y or PY to 1.3**, answer questions 1.7 and 1.8, which relate to time-varying confounding		

	**1.7. Did the authors use an appropriate analysis method that adjusted for all the critically important confounding areas and for time-varying confounding?**	NA / Y / PY / PN / N / NI	Yes, see 1.4.<br><br>The final model was adjusted for age, gender, school education, smoking, WC and the frequency of dental visits (in the last 12 months). Effect modifications were assessed including interaction terms between confounders and the exposure variable in the multivariable models. Sensitivity analyses were run to assess the association between T1DM, T2DM and periodontal disease by changing disease definition to verify the stability of findings regarding the association between both diabetes types and periodontitis.

	**1.8. If Y or PY to 1.7: Were confounding areas that were adjusted for measured validly and reliably by the variables available in this study?**	NA / Y / PY / PN / N / NI	T1DM and T2DM occur at different age groups. The analyses were performed in different age groups. The study enabled a valid evaluation.
	**Risk of bias judgement**	**Low**	**Potential confounding but the analysis was performed in different age groups and the study adjusted for several confounders, such as age, smoking and co-morbidities. Thereby effect modifications were assessed and sensitivity analysis were made.**
	Optional: What is the predicted direction of bias due to confounding?	**Favors experimental**	Bias could lead to an inaccurate underestimate and potential bias favours the experimental group.
Bias in selection of participants into the study	**2.1. Was selection of participants into the study (or into the analysis) based on variables measured after the start of the exposure?**<br><br>**<u>If N or PN to 2.1 go to 2.4</u>**	**Y** / PY / PN / N / NI	The Study of Health in Pomerania (SHIP) is a population-based survey, including a medical and dental examination of the adult population in a northeast region of Germany. Details about the study population, recruitment and examinations have been published elsewhere (John et al. 2001).<br><br>*John, U., Greiner, B., Hensel, E., Ludemann, J., Piek, M. & Sauer, S. (2001) Study of Health In Pomerania (SHIP): a health examination survey in an east German region: objectives and design. Sozial- und Praventivmedizin 46, 186–194.*
	**2.2. <u>If Y/PY to 2.1:</u> Were the post-exposure variables that influenced selection associated with exposure?**	Y / PY / PN / N / NI	Yes, it is possible that the severity of diabetes changes over time. Because of the cross-sectional design, there were no detailed

			information about previous glycemic control.
	**2.3. If Y/PY to 2.2: Were the post-exposure variables that influenced eligibility selection influenced by the outcome or a cause of the outcome?**	NA / Y / PY / PN / N / NI	Yes, see figure 1.
	**2.4 Do start of follow-up and start of exposure coincide for most participants?**	NA / Y / PY / PN / **N** / NI	No, the start of exposure already exists prior of the study. Thereby the severity changes between participants.
	**2.5 If Y/PY to 2.2 and 2.3, or N/PN to 2.4: Were adjustment techniques used that are likely to correct for the presence of selection biases?**	NA / Y / PY / PN / N / **NI**	No information given about adjustment techniques.
	**Risk of bias judgement**	**Low**	**Large sample size and the selection into the study is well described (also by using a clear figure).**<br><br>**Some problems, such as post-exposure variables, are related to the cross-sectional design of the study.**
Bias in classification of exposures	**3.1 Is exposure status well defined?**	Y / PY / PN / N / NI	Yes, T1DM and T2DM is clearly defined. Subhead definition of diabetes.
	**3.2 Did entry into the study begin with start of the exposure?**	Y / PY / PN / N / NI	Yes, because of the cross-sectional design. Because of this there were no detailed information on the reasons for and the timing of tooth loss, previous periodontal treatment and previous glycemic control.<br><br>It is possible that there will be undiagnosed diabetics in the non-diabetic (control) group.
	**3.3 Was information used to define exposure status recorded prior to outcome assessment?**	Y / PY / PN / N / NI	No information about the exposure status (severity of diabetes), only

			distinction between diabetes type II and controls.
	**3.4 Could classification of exposure status have been affected by knowledge of the outcome or risk of the outcome?**	Y / PY / PN / N / NI	No, the data of the participants is recruited somewhere else prior of the start of the study; Centre of Cardiology of Diabetes of SHIP cohort. Classification could not have been affected by knowledge of the outcome.
	**3.5 Were exposure assessment methods robust (including methods used to input data)?**	Y / PY / PN / N / **NI**	No information.
	**Risk of bias judgement**	**Low**	**T1DM and T2DM were clearly defined to reduce the misclassification.**
	Optional: What is the predicted direction of bias due to measurement of outcomes or exposures?	**Favors experimental**	It is possible that there will be undiagnosed diabetics in the non-diabetic (control) group which leads to the predicted direction of bias favours experimental, because of the underestimation in the DM group.
Bias due to departures from intended exposures	**4.1. Is there concern that changes in exposure status occurred among participants?**<br><br>**If your aim for this study is to assess the effect of initiating and adhering to an exposure (as in a per-protocol analysis), answer questions 4.2 and 4.3, otherwise continue to 4.4 if Y or PY to 4.1.**	Y / PY / PN / N / NI	Yes, see chapter 3.2<br><br>Yes, because it is a cross-sectional design. Because of this there were no detailed information on the reasons for and the timing of tooth loss, previous periodontal treatment and previous glycemic control.<br><br>For example; teeth with worse periodontal disease might have been extracted; hence the remaining teeth may not represent the long-term periodontal status.

	4.2. Did many participants switch to other exposures?	Y / PY / PN / N / NI	It is possible that severity of diabetes changes over time.
	4.3. Were the critical co-exposures balanced across exposure groups?	**Y** / PY / PN / N / NI	These subjects in the T1DM cohort recruited from the Centre of Cardiology and Diabetes, Karlsburg and the SHIP cohort lived in the same geographical region.<br><br>T1DM subjects were younger, but did not differ considerably with regard to education and smoking habits compared with non-diabetic subjects T2DM subjects were less educated, more obese and more frequently former smokers than non-diabetic subjects<br>(see Table 1 of the study; Demographic, medical, and dental characteristics of the study population in T1DM versus non-diabetic subjects aged 20–59 years and<br>T2DM versus non-diabetic subjects aged 50–81 years)
	4.4. If NY/PN PY to 4.1, or Y/PY to 4.2, or 4.3: Were adjustment techniques used that are likely to correct for these issues?	NA / Y / PY / PN / **N** / NI	No, not possible because of the cross-sectional design.
	**Risk of bias judgement**	**Low**	**Exposure status is well defined. Not likely that the intended exposures bias the results. The authors distinguish DM between type I and type II which is a strength of the present study.**
Bias due to missing data	5.1 Were there missing outcome data?	Y / PY / PN / N / NI	One limitation may exist due to missing evaluation of the oral glucose tolerance test and non-fasting glucose values
	5.2 Were participants excluded due to missing data on exposure status?	Y / PY / PN / N / NI	Yes, see figure 1. Missing data about periodontal measures of missing confounder data:<br><br>1. Type I: 46 excluded for missing

			data (1 without oral examination + 20 no AL measurements + 25 missing confounder data)<br><br>2.Type I, non-DM (SHIP): 156 excluded for missing data (14 without oral examination + 130 no AL measurements + 12 missing confounder data)<br><br>3.Type II, DM (SHIP) 129 excluded for missing data (1 without oral examination + 128 no AL measurement)<br><br>4.Type II, non-DM (SHIP) 572 excluded for missing data (6 without oral examination + 553 no AL measurements + 13 missing confounder data)
	**5.3 Were participants excluded due to missing data on other variables needed for the analysis?**	**Y** / PY / PN / N / NI	Yes, excluded because of missing data for potential confounders: age, gender, school education, smoking, WC and the frequency of dental visits<br>in the last 12 months), see above.
	**5.4 <u>If Y/PY to 5.1, 5.2 or 5.3:</u> Are the proportion of participants and reasons for missing data similar across exposures?**	NA / Y / PY / PN / N / NI	No not necessary, because the missing data already exist before start of the study. Participants with these specific missing data were excluded, so only participants with complete data participated.

	**5.5 <u>If Y/PY to 5.1, 5.2 or 5.3:</u> Were appropriate statistical methods used to account for missing data?**	NA / Y / PY / PN / N / NI	No information.
	**Risk of bias judgement**	**Low**	**Data is complete. Missing data exist prior by start of the study. The sample only consist of people with complete data, because participants with missing values were excluded for analysis.**
	Optional: What is the predicted direction of bias due to missing data?	Towards null	
Bias in measurement of outcomes	**6.1 Could the outcome measure have been influenced by knowledge of the exposure received?**	Y / PY / PN / **N** / NI	Detailed description about periodontal measurements:<br>- AL represents the distance from the cemento-enamel junction to the bottom of the periodontal pocket.<br>- PD represents the distance from the gingival margin to the base of the periodontal pocket.<br>Registered according to the half-mouth method and by using the same periodontal probe.<br><br>**The number of teeth was determined full mouth on a maximum of 28 teeth.** All fully erupted teeth, except third molars.
	**6.2 Was the outcome measure sensitive?**	Y / PY / PN / **N** / NI	The outcome measurement is a surrogate/hard endpoint and not sensitive (for change).<br><br>The number of teeth was determined full mouth on a maximum of 28 teeth.

			All fully erupted teeth, except third molars.
	**6.3 Were outcome assessors unaware of the exposure received by study participants?**	**Y** / PY / PN / N / NI	Yes: calibrated licensed dentists performed all the examinations. Every 6–12 months, calibration exercises were performed on a subset of persons not connected to the study.
	**6.4 Were the methods of outcome assessment comparable across exposure groups?**	**Y** / PY / PN / N / NI	Yes, same for both groups. Same dentist performed the examinations.
	**6.5 Were any systematic errors in measurement of the outcome unrelated to exposure received?**	Y / PY / PN / **N** / NI	Inter and intraclass correlations were calculated.
	**Risk of bias judgement**	**Low**	**The methods of outcome assessment were comparable across exposure groups and clearly described.**
Bias in selection of	Is the reported effect estimate likely to be selected, on the basis of the results, from...?		
the reported result	**7.1. ... multiple outcome *measurements* within the outcome domain?**	**Y** / PY / PN / N / NI	Multiple outcomes: AL, PD and number of missing teeth are fully described.
	**7.2 ... multiple *analyses* of the exposure-outcome relationship?**	Y / PY / PN / N / **NI**	Yes, several models (for adjustment) are created and reported in the study.
	**7.3 ... different *subgroups*?**	**Y** / PY / PN / N / NI	Yes, analysis was performed in different age groups. .
	**Risk of bias judgement**	**Low**	**All the data were fully described.**
	Optional: What is the predicted direction of bias due to selection of the reported result?	**Favors experimental**	Publication bias leads to predicted direction of bias favours experimental.
Overall bias	**Risk of bias judgement**	**Low**	**The major strength of the study is the large sample size comprising a wide age range of social and**

			**medical data permitting the estimation with good statistical precision. Thereby, the authors used linear and multivariable regressions models, effects modifications were assessed, and sensitivity analyses were made. Finally, a major strength is the distinction between diabetes type I and II.**<br><br>**Due the cross-sectional design, there was no detailed information on the reasons for and the timing of tooth loss.**

Online Appendix S2-9

Preliminary tool for risk of bias in exposure study: Patiño-Marín et al. 2008

Title: CARIES, PERIODONTAL DISEASE AND TOOTH LOSS IN PATIENTS WITH DIABETES MELLITUS TYPES 1 AND 2

Year: 2008

Authors: Nuria Patiño Marín (a), Juan P. Loyola Rodríguez (b), Carlo E. Medina Solis (c), América P. Pontigo Loyola (c), Juan F. Reyes Macías (4), Jenny C. Ortega Rosado (a), Celia Aradillas García €

a: Laboratory of Clinical Investigation.

b: Laboratory of Molecular Biology and Oral Microbiology. Faculty of Dentistry, Autonomous University of San Luis Potosí, México.

c: Academic Area of Dentistry of the Institute of Health Sciences of the Autonomous University of the State of Hidalgo. Pachuca, Hidalgo. Mexico.

d: Department of Pathology, Autonomous University of San Luis Potosí, México.

e: Faculty of Medicine, Autonomous University of San Luis Potosí, México.

PMID: 19177848

AIM: To determine the frequency of caries, periodontal disease and tooth loss in patients affected by diabetes mellitus type 1 and 2.

Specify a target experiment specific to the study:

☐	The protocol-specified target experiment fully applies	***OR***	Participant<br><br>Experimental exposure<br><br>Control exposure	**Participants:**<br>Cross-sectional study was conducted from October 2004 to November 2007. A total of 175 subjects participated.<br>**Experimental exposure:**<br>**105 diabetic patients:**<br>1. 35 patients with diabetes type I → with glycosylated hemoglobin values between 6.5% and 7%),<br>2. 35 patients with diabetes type I → with glycosylated hemoglobin values higher than 7%)<br>1. 35 patients with diabetes type II<br>**Control exposure:**<br>**70 Non-diabetic patients:**<br>1. 35 subjects without diabetes mellitus type I<br>2. 35 subjects without diabetes mellitus type

Specify the outcome

Specify which outcome is being assessed for risk of bias (typically from among those earmarked for the Summary of Findings table). Specify whether this is a proposed benefit or harm of exposure.

All data were expressed as mean, standard deviation, frequencies and percentage.

To determine the distribution of the variables, the Shapiro-Wilk and Brown- Forsyte statistical tests were used. The statistical tests used for analysing the data were U. Mann Whitney and Kruskal Wallis to compare quantitative variables, Chi Square for qualitative variables, and Spearman statistical test for correlations among variables.

Logistic regression was used to calculate the odds ratio (with a 95% confidence interval) in the group of patients with diabetes type 2. In the analysis,
the dependent variable was presence or absence of loss of attachment level and the independent variables were missing teeth, periodontal pocket probing depth and diabetes.

Is your aim for this study…?

The controversy in literature and the small number of studies on the Mexican population, in which there is high prevalence of diabetes, are the main reasons for identifying frequency of caries, periodontal disease and missing teeth, with the aim of reporting the frequencies of these oral manifestations in patients with diabetes types 1 and 2, to produce data that will contribute towards the establishment of oral health prevention programs or systems in the future.

■ to assess the effect of initiating intervention (as in an intention-to-treat analysis)

☐ to assess the effect of initiating and adhering to intervention (as in a per-protocol analysis)

☐ other (specify)

Specify the numerical result being assessed

In case of multiple alternative analyses being presented, specify the numeric result (e.g. RR = 1.52 (95% CI 0.83 to 2.77) and/or a reference (e.g. to a table, figure or paragraph) that uniquely defines the result being assessed.

All data were expressed as mean ± standard deviation, frequencies and percentage. To determine the distribution of the variables, the Shapiro-Wilk and Brown- Forsyte statistical tests were used. The statistical tests used for analysing the data were U. Mann Whitney and Kruskal Wallis to compare quantitative variables, Chi Square for qualitative variables, and Spearman statistical test for correlations among variables. Logistic regression was used to calculate the odds ratio (with a 95% confidence interval) in the group of patients with diabetes type 2. In the analysis, the dependent variable was presence or absence of loss of attachment level and the independent variables were missing teeth, periodontal pocket probing depth and diabetes. And imposed alpha levels of $p<0.05$ is used.

Preliminary consideration of confounders

Complete a row for each important confounding area (i) listed in the review protocol; and (ii) relevant to the setting of this particular study, or which the study authors identified as potentially important. *"Important" confounding areas are those for which, in the context of this study, adjustment is expected to lead to a clinically important change in the estimated effect of the exposure. "Validity" refers to*

whether the confounding variable or variables fully measure the area, while "reliability" refers to the precision of the measurement (more measurement error means less reliability).

**(xv) Confounding areas listed in the review protocol**				
Confounding area	Measured variable(s)	Is there evidence that controlling for this variable was unnecessary?*	Is the confounding area measured validly and reliably by this variable (or these variables)?	OPTIONAL: Is adjusting for this variable (alone) expected to move the effect estimate up or down?
			No information	No information

(xvi) **Additional confounding areas relevant to the setting of this particular study, or which the study authors identified as important**				
Confounding area	Measured variable(s)	Is there evidence that controlling for this variable was unnecessary?*	Is the confounding area measured validly and reliably by this variable (or these variables)?	OPTIONAL: Is adjusting for this variable (alone) expected to move the effect estimate up or down?
			No information	No information

* In the context of a particular study, variables can be demonstrated not to be confounders and so not included in the analysis: (a) if they are not predictive of the outcome; (b) if they are not predictive of exposure; or (c) because adjustment makes no or minimal difference to the estimated effect of the primary parameter. Note that "no statistically significant association" is not the same as "not predictive".

Preliminary consideration of criteria used to determine the accuracy of measurement of exposure and outcome

Complete a row for each measure listed in the study for the (i) exposure and (ii) outcome. Of the measures listed in the protocol, consider the sensitivity, specificity, and confidence in the methods used in the study.

(xv) Exposure measurement method listed in the study		
Method of measurement	Measured exposure	Is the exposure measured validly and reliably by this method (or these methods)?
Professionally diagnosed<br><br>Type I based on hemoglobin values and blood glucose levels professionally diagnosed.<br><br>Type II professionally diagnosed<br><br>Controls based on glucose values and BMI index	Diabetes mellitus	**Group 1**: patients with diabetes type I with glycosylated hemoglobin values between 6.5% and 7% and blood glucose levels under 110 mg/dl., of either sex, aged 8 to 30 years, with 5 or more years evolution of diabetes as from diagnosis, and without arterial hypertension.<br><br>**Group 2:** patients with diabetes type I with glycosylated hemoglobin values higher than 7% and blood glucose levels higher than 110 mg/dl., of either sex, aged 8 to 30 years, with 5 or more years evolution of diabetes as from diagnosis, and without arterial hypertension.<br><br>**Group 3 (control):** subjects without diabetes type I, of either sex, aged 8 to 30 years, with glucose values < 110 mg/dl, with body mass index (height and weight) < 27 Kg/m2 and without arterial hypertension.<br><br>**Group 4:** patients with diabetes type II, of either sex, aged 30 to 60 years, with 5 or more year's evolution of diabetes as from diagnosis and without arterial hypertension.<br><br>**Group 5 (control):** subjects without diabetes type II, of either sex, aged 30 to 60 years, with glucose values < 110 mg/dl, with a body mass index < 27 Kg/m2 and without arterial hypertension.

(xvi) Outcome measurement method listed in the study		
Method of measurement	Measured outcome	Is the outcome measured validly and reliably by this method (or these methods)?
Professionally blind (regarding the diabetes diagnosis) evaluation	1) Frequency of caries, filled teeth, missing teeth and prosthetic restoration (fixed, removable and full denture). Fixed and	Plaque index: (Silness and Loe, 1964)<br>Calculus index: (Greene and Vermillion 1964)<br>Periodontal Evaluation were recorded with a calibrated periodontal probe graduated in mm (Hu-friendy)

	removable dentures were evaluated as number of replacement teeth.<br><br>2) DMFT index –the sum of decayed, missing and filled teeth.<br><br>3) Oral hygiene - Plaque Index – the presence of dentobacterial plaque on all tooth surfaces was recorded<br>Calculus Index – the presence of<br>supra and subgingival calculus was evaluated on 4 tooth surfaces (mesial, distal, buccal and lingual),<br>and the average calculated.<br><br>4) Periodontal Evaluation – probing depth<br>and loss of epithelial attachment were recorded.	Probing depth was measured from the gingival margin to the base of the pocket, considering a healthy sulcus as < 3 mm. The level of epithelial attachment was evaluated from the cemento-enamel junction to the base of the sulcus, considering a healthy sulcus as < 2 mm.

Preliminary consideration of co-exposures

Complete a row for each important co-intervention (i) listed in the review protocol; and (ii) relevant to the setting of this particular study, or which the study authors identified as important.
"Important" co-interventions are those for which, in the context of this study, adjustment is expected to lead to a clinically important change in the estimated effect of the intervention.

**(xv) Co-exposures listed in the review protocol**		
Co-exposure	Is there evidence that controlling for this co-exposure was unnecessary (e.g., because it was not administered)?	Is presence of this co-exposure likely to favor outcomes in the experimental or the control group
		Favor experimental / Favor comparator / No information
		Favor experimental / Favor comparator / No information
		Favor experimental / Favor comparator / No information

**(xvi) Additional co-exposures relevant to the setting of this particular study, or which the study authors identified as important**		
Co-exposure	Is there evidence that controlling for this co-exposure was unnecessary (e.g., because it was not administered)?	Is presence of this co-exposure likely to favor outcomes in the experimental or the control group
		Favor experimental / Favor comparator / No information
		Favor experimental / Favor comparator / No information
		Favor experimental / Favor comparator / No information

Risk of bias assessment (cohort-type studies)

Bias due to confounding	**1.1 Is there potential for confounding of the effect of exposure in this study? If N or PN to 1.1:** the study can be considered to be at low risk of bias due to confounding and no further signalling questions need be considered	**Y** / PY / PN / N / NI	Cross-sectional design, but there is no detailed information given about potential confounders.<br><br>Only age, BMI glucose, glycosylated hemoglobin, mean blood pressure, sex and hereditary family history for all study groups is evaluated and described in table 1.
	**If Y/PY to 1.1, answer 2.1 and 1.3 to determine whether there is a need to assess time-varying confounding:**		
	**1.2. If Y or PY to 1.1: Was the analysis based on splitting, follow up time according to exposure received?**<br><br>**If N or PN to 1.2**, answer questions 1.4 to 1.6, which relate to baseline confounding	NA / Y / PY / PN / **N** / NI	The participants could not switch between exposures, so the outcome could not be biased due time varying confounding. Difference in exposure status is limited at the start of the study because the authors made a distinction between diabetes type I and II. For type I a further division in HbA1 levels.
	**1.3. If Y or PY to 1.2: Were exposure discontinuations or switches likely to be related to factors that are prognostic for the outcome?**	NA / **Y** / PY / PN / N / NI	Yes, studies have reported that there is an association between DM (exposure status) and the presence of risk markers; age, sex, race, frequency of dental visits, plaque, hemoglobin A1c, duration of diabetes BMI and periodontitis.
	**If N or PN to 1.3**, answer questions 1.4 to 1.6, which relate to baseline confounding		
	**1.4. Did the authors use an appropriate analysis method that adjusted for all the critically important confounding areas?**	NA / Y / PY / PN / **N** / NI	No.<br><br>Only age, BMI glucose, glycosylated hemoglobin, mean blood pressure, sex and hereditary family history for all study groups is evaluated and

			described in table 1. No appropriate methods to control for these potential confounders is measured, such as stratification, matching. Only logistic regression is used to calculate the odds ratio in the group of patients with diabetes type 2.
	1.5. If Y or PY to 1.4: Were confounding areas that were adjusted for measured validly and reliably by the variables available in this study?	NA / Y / PY / PN / N / NI	No information.
	1.6. Did the authors avoid adjusting for post-exposure variables?	NA / Y / PY / PN / **N** / NI	No information about controlling for post exposure variables that are affected by exposures is described. This probably not happened, because no correction has even made for potential confounding.
	**If Y or PY to 1.3**, answer questions 1.7 and 1.8, which relate to time-varying confounding		

	1.7. Did the authors use an appropriate analysis method that adjusted for all the critically important confounding areas and for time-varying confounding?	NA / Y / PY / PN / **N** / NI	No, see 1.4
	1.8. If Y or PY to 1.7: Were confounding areas that were adjusted for measured validly and reliably by the variables available in this study?	NA / Y / PY / PN / N / NI	
	**Risk of bias judgement**	**Moderate**	**Only logistic regression was used to calculate the odds ratio in the group of patients with diabetes type 2. A detailed description about any potential confounding domains is missing or not described.**

	Optional: What is the predicted direction of bias due to confounding?	Unpredictable	
Bias in selection of participants into the study	**2.1. Was selection of participants into the study (or into the analysis) based on variables measured after the start of the exposure?**<br><br>**If N or PN to 2.1 go to 2.4**	**Y** / PY / PN / N / NI	The subjects, selected by means of consecutive non-probabilistic sampling, met the following criteria:<br>**Group 1**: patients with diabetes type I with glycosylated hemoglobin values between 6.5% and 7% and blood glucose levels under 110 mg/dl., of either sex, aged 8 to 30 years, with 5 or more years evolution of diabetes as from diagnosis, and without arterial hypertension.<br><br>**Group 2:** patients with diabetes type I with glycosylated hemoglobin values higher than 7% and blood glucose levels higher than 110 mg/dl., of either sex, aged 8 to 30 years, with 5 or more years evolution of diabetes as from diagnosis, and without arterial hypertension.<br><br>**Group 3 (control):** subjects without diabetes type I, of either sex, aged 8 to 30 years, with glucose values < 110 mg/dl, with body mass index (height and weight) < 27 Kg/m2 and without arterial hypertension.<br><br>**Group 4:**<br>patients with diabetes type II, of either sex, aged 30 to 60 years, with 5 or more year's evolution of diabetes as from

			diagnosis and without arterial hypertension.<br><br>**Group 5 (control):** subjects without diabetes type 2, of either sex, aged 30 to 60 years, with glucose values < 110 mg/dl, with a body mass index < 27 Kg/m2 and without arterial hypertension.<br><br>The exposure status already exists prior at the start of the study.
	**2.2. <u>If Y/PY to 2.1:</u> Were the post-exposure variables that influenced selection associated with exposure?**	Y / PY / PN / **N** / NI	
	**2.3. <u>If Y/PY to 2.2:</u> Were the post-exposure variables that influenced eligibility selection influenced by the outcome or a cause of the outcome?**	NA / **Y** / PY / PN / N / NI	Exclusion criteria for all groups were pregnancy, patients with evident genetic pathologies, treatment of periodontal disease, treatment for epilepsy or kidney transplant, but these exclusion criteria made an more reliably outcome.
	**2.4 Do start of follow-up and start of exposure coincide for most participants?**	NA / Y / PY / PN / **N** / NI	No distinction is made in duration of diabetes.
	**2.5 If Y/PY to 2.2 and 2.3, or N/PN to 2.4: Were adjustment techniques used that are likely to correct for the presence of selection biases?**	NA / Y / PY / PN / **N** / NI	No adjustment techniques are used to correct for selection bias.
	**Risk of bias judgement**	**Moderate**	**Selection bias is moderate. They used a non-probabilistic sampling method selected by means of consecutive sampling. Which gave not all the individuals in the Mexican population an equal chance of being selected.**

			**For the SR, we excluded group 1, 2 and 3 because these groups also consistent of participants <18 years. We only include adults in the meta-analysis.**
Bias in classification of exposures	**3.1 Is exposure status well defined?**	**Y** / PY / PN / N / NI	Yes, detailed description in the subhead materials and methods about the structure and composition of the 5 groups.
	**3.2 Did entry into the study begin with start of the exposure?**	Y / PY / PN / **N** / NI	No, distinction is made in 5 groups. Undiagnosed diabetics might be classified as a non-diabetic group.<br><br>The exposure status already exists prior at the start of the study, but the severity of DM may differ within the groups. They defined multiple groups, for example for type I DM, to distinguish in the severity of DM based on HbA1 levels.
	**3.3 Was information used to define exposure status recorded prior to outcome assessment?**	Y / PY / PN / N / **NI**	A lot of studies used the WHO classification to define DM status in several categories, but no specific information about the source and the distribution of the participants into the 5 groups is given.
	**3.4 Could classification of exposure status have been affected by knowledge of the outcome or risk of the outcome?**	Y / PY / PN / N / NI	No, a blind (regarding the diabetes diagnosis) evaluation is made of the clinical parameters on all teeth of the participating subjects.
	**3.5 Were exposure assessment methods robust (including methods used to input data)?**	Y / PY / PN / N / **NI**	-
	**Risk of bias judgement**	**Low**	**The authors made a detailed description of the exposure status and the distribution of the participants into the 5 different**

			**groups. Thereby, the information is collected at the time when the exposure status already exists and not determined retrospectively.**
Bias due to departures from intended exposures	**4.1. Is there concern that changes in exposure status occurred among participants?**<br><br>**If your aim for this study is to assess the effect of initiating and adhering to an exposure (as in a per-protocol analysis), answer questions 4.2 and 4.3, otherwise continue to 4.4 if Y or PY to 4.1.**	**Y** / PY / PN / N / NI	Yes, but this is inevitable by diabetes mellitus. Severity of the disease changes over time.
	**4.2. Did many participants switch to other exposures?**	Y / PY / PN / N / NI	Undiagnosed diabetics might be classified as a non-diabetic group.
	**4.3. Were the critical co-exposures balanced across exposure groups?**	Y / PY / PN / N / NI	Yes, detailed description for each different group is made in table 1.
	**4.4. If NY/PN PY to 4.1, or Y/PY to 4.2, or 4.3: Were adjustment techniques used that are likely to correct for these issues?**	NA / Y / PY / PN / N / NI	No they only used logistic regression to calculated the odds ratio in the group of patients with diabetes type 2
	**Risk of bias judgement**	**Low**	**There were deviations from usual practice, but their impact on the outcome is expected to be slight, because this is inevitable by the disease.**<br><br>**We excluded group 1, 2 and 3 (DM type I) in the SR. So only focus on diabetes type II.**
Optional: What is the predicted direction of bias due to departures from the	**Favours experimental**	If there were undiagnosed diabetics in the non-diabetic group, the predicted direction of bias is probably favours experimental.	

intended exposures?			
Bias due to missing data	**5.1 Were there missing outcome data?**	Y / PY / PN / N / **NI**	Not reported.
	**5.2 Were participants excluded due to missing data on exposure status?**	Y / PY / PN / N / **NI**	Likely, but not reported.
	**5.3 Were participants excluded due to missing data on other variables needed for the analysis?**	Y / PY / PN / N / **NI**	Likely, but not reported.
	**5.4 If Y/PY to 5.1, 5.2 or 5.3: Are the proportion of participants and reasons for missing data similar across exposures?**	NA / Y / PY / PN / N / **NI**	Not reported.
	**5.5 If Y/PY to 5.1, 5.2 or 5.3: Were appropriate statistical methods used to account for missing data?**	NA / Y / PY / PN / **N** / NI	No.
	**Risk of bias judgement**	**NI**	**No information is reported about missing data or the potential for data to be missing. It is likely that there will be no missing data.**
Bias in measurement of outcomes	**6.1 Could the outcome measure have been influenced by knowledge of the exposure received?**	Y / PY / PN / **N** / NI	No.
	**6.2 Was the outcome measure sensitive?**	Y / PY / PN / N / NI	Only outcome measures for the other periodontal parameters, such as probing pocket depth of epithelial attachments loss, but missing teeth is a surrogate, hard, endpoint.<br><br>For the sensitive outcomes a detailed description about the measurement method is made and ICC were calculated.<br><br>For example, probing depth: Probing depth was measured from the gingival margin to the base of the pocket, considering a healthy sulcus as < 3 mm using a calibrated periodontal probe graduated in mm.

	**6.3 Were outcome assessors unaware of the exposure received by study participants?**	**Y** / PY / PN / N / NI	Yes, a blind (regarding the diabetes diagnosis) evaluation was made of the clinical parameters on all teeth of the participating subjects.<br><br>The examiner underwent standardization for all variables during a pilot study on a total 60 subjects.
	**6.4 Were the methods of outcome assessment comparable across exposure groups?**	Y / PY / PN / N / NI	Yes, all the 5 groups underwent the same outcome assessments.<br>1. Frequency of caries, filled teeth, missing teeth and prosthetic restoration<br>2. DMFT index<br>3. Oral hygiene
	**6.5 Were any systematic errors in measurement of the outcome unrelated to exposure received?**	Y / PY / PN / **N** / NI	Intra- and inter-observer data reproducibility was evaluated with Kappa and the intraclass correlation Coefficient.<br><br>Inter- and intraobserver standardization for all variables had concordance greater than 0.80. Concluded in a reliable and consistent measurement for all variables.
	**Risk of bias judgement**	**Low**	**The methods of outcome assessment were comparable across the groups and the outcome measure is not being influenced by knowledge of the exposure status, because the evaluation is made blind regarding the diabetes diagnosis.**

Bias in selection of	Is the reported effect estimate likely to be selected, on the basis of the results, from...?		
the reported result	**7.1. ... multiple outcome *measurements* within the outcome domain?**	**Y** / PY / PN / N / NI	All the results about the clinical parameters were reported.
	**7.2 ... multiple *analyses* of the exposure-outcome relationship?**	**Y** / PY / PN / N / NI	All the multiple analysis has been reported.
	**7.3 ... different *subgroups*?**	**Y** / PY / PN / N / NI	No distinction in specific subgroups is made, for example stratification in age. All the results for the several groups have been reported.
	**Risk of bias judgement**	**Low**	**All the results have been reported.**
	Optional: What is the predicted direction of bias due to selection of the reported result?	Unpredictable	If there were any concern about publication bias; the predicted direction of bias is favouring the best possible outcome for the authors.
Overall bias	**Risk of bias judgement**	**Moderate**	**Serious risk of bias. The cross-sectional study design is subject to confounding and the authors did not/report to short the adjustment for potential confounders. Only a short description that logistic regression is used to calculate the odds ratio.**<br><br>**Thereby, potential selection bias because a non-probabilistic sampling method selected by means of consecutive sampling is used. Which gave not all the individuals an equal chance of being selected.**

Online Appendix S2-10

Preliminary tool for risk of bias in exposure study: Bacic et al. 1989

Title: Dental status in a group of adult diabetic patients

Year: 1989

Authors: Bacić M, Ciglar I, Granić M, Plancak D, Sutalo J.

PMID: 2591185

***AIM**: The aim of the study is to determine the prevalence of dental caries, DMFT level and treatment needs in a group of adult diabetic patients, and to compare them to those recorded in a control group as well as to elucidate any possible effects of the type, duration, and control of diabetes on the variables under study.*

Specify a target experiment specific to the study:

☐ The protocol-specified target experiment fully applies ***OR*** Participant / Experimental exposure / Control exposure

Participant:
Adults in Croatia.

Experimental exposure:
Random sample of 222 dentate diabetic patients (130 males, 92 females), mean age 49.6 years. Patients were selected from the Vuk Vrhovac Institute of Diabetes Endocrinology and Metabolic Diseases, in Zagreb and they had been referred from all parts of Croatia.

1. 109 insulin-dependent, type I
2. 113 non-insulin-dependent, type II

Mean duration of diabetes of 11 years.

Control exposure:
189 dentate subjects (115 males, 74 females), mean age 43.9, with no suggestion of diabetes in their medical history. They were taken from the general population of Croatia during a survey on the prevalence of periodontal disease and caries in Croatia.

Specify the outcome

Specify which outcome is being assessed for risk of bias (typically from among those earmarked for the Summary of Findings table). Specify whether this is a proposed benefit or harm of exposure.

DMFT: a common method for assessing dental caries prevalence as well as dental treatment needs among patients.

- Decayed teeth
- Missing teeth
- Filled teeth

Is your aim for this study…?

■ to assess the effect of initiating intervention (as in an intention-to-treat analysis)

☐ to assess the effect of initiating and adhering to intervention (as in a per-protocol analysis)

☐ other (specify)

Specify the numerical result being assessed

In case of multiple alternative analyses being presented, specify the numeric result (e.g. RR = 1.52 (95% CI 0.83 to 2.77) and/or a reference (e.g. to a table, figure or paragraph) that uniquely defines the result being assessed.

Statistical evaluation of the data for both groups was made by the STATJOB program package using analysis of variance and the results were compared between the groups.

Preliminary consideration of confounders

No consideration or correction for any potential confounders, only an distinction between the age groups is measured.

Complete a row for each important confounding area (i) listed in the review protocol; and (ii) relevant to the setting of this particular study, or which the study authors identified as potentially important. *"Important" confounding areas are those for which, in the context of this study, adjustment is expected to lead to a clinically important change in the estimated effect of the exposure. "Validity" refers to whether the confounding variable or variables fully measure the area, while "reliability" refers to the precision of the measurement (more measurement error means less reliability).*

**(xvii) Confounding areas listed in the review protocol**				
Confounding area	Measured variable(s)	Is there evidence that controlling for this variable was unnecessary?*	Is the confounding area measured validly and reliably by this variable (or these variables)?	OPTIONAL: Is adjusting for this variable (alone) expected to move the effect estimate up or down?
			Yes / No / No information	Favor intervention / Favor control / No information

(xviii) **Additional confounding areas relevant to the setting of this particular study, or which the study authors identified as important**				
Confounding area	Measured variable(s)	Is there evidence that controlling for this variable was unnecessary?*	Is the confounding area measured validly and reliably by this variable (or these variables)?	OPTIONAL: Is adjusting for this variable (alone) expected to move the effect estimate up or down?
			Yes / No / No information	Favor intervention / Favor control / No information

* In the context of a particular study, variables can be demonstrated not to be confounders and so not included in the analysis: (a) if they are not predictive of the outcome; (b) if they are not predictive of exposure; or (c) because adjustment makes no or minimal difference to the estimated effect of the primary parameter. Note that “no statistically significant association” is not the same as “not predictive”.

Preliminary consideration of criteria used to determine the accuracy of measurement of exposure and outcome

Complete a row for each measure listed in the study for the (i) exposure and (ii) outcome. Of the measures listed in the protocol, consider the sensitivity, specificity, and confidence in the methods used in the study.

(xvii) Exposure measurement method listed in the study		
Method of measurement	Measured exposure	Is the exposure measured validly and reliably by this method (or these methods)?
Professionally diagnosed	Diabetes	C-peptide findings, duration of diabetes, mean blood glucose (MGB), HbA1 level, and the presence of diabetic complications, i.e. neuropathy and retinopathy.<br><br>All data were recorded in the WHO combined oral health and treatment needs assessments forms, according to the instructions by the WHO.

(xviii) Outcome measurement method listed in the study		
Method of measurement	Measured outcome	Is the outcome measured validly and reliably by this method (or these methods)?
Clinical examination	DMFT	Decayed, filled and missing teeth were recorded. Only cavities extending into the dentine were registered. No radiographs were taken. Instruments used for the examination were a standard probe and a mirror.<br><br>All data were recorded in the WHO combined oral health and treatment needs assessments forms, according to the instructions by the WHO.

Preliminary consideration of co-exposures

Complete a row for each important co-intervention (i) listed in the review protocol; and (ii) relevant to the setting of this particular study, or which the study authors identified as important.
"Important" co-interventions are those for which, in the context of this study, adjustment is expected to lead to a clinically important change in the estimated effect of the intervention.

**(xvii) Co-exposures listed in the review protocol**		
Co-exposure	Is there evidence that controlling for this co-exposure was unnecessary (e.g., because it was not administered)?	Is presence of this co-exposure likely to favor outcomes in the experimental or the control group
		Favor experimental / Favor comparator / No information
		Favor experimental / Favor comparator / No information
		Favor experimental / Favor comparator / No information

**(xviii) Additional co-exposures relevant to the setting of this particular study, or which the study authors identified as important**		
Co-exposure	Is there evidence that controlling for this co-exposure was unnecessary (e.g., because it was not administered)?	Is presence of this co-exposure likely to favor outcomes in the experimental or the control group
		Favor experimental / Favor comparator / No information
		Favor experimental / Favor comparator / No information
		Favor experimental / Favor comparator / No information

Risk of bias assessment (cohort-type studies)

Bias due to confounding	**1.1 Is there potential for confounding of the effect of exposure in this study? If N or PN to 1.1:** the study can be considered to be at low risk of bias due to confounding and no further signalling questions need be considered	**Y** / PY / PN / N	Yes, a limitation of the study is its cross-sectional design and the potential confounding of the effect of exposure in the study.<br><br>For example: diabetics have more meals daily than normal subjects which, together with a high level of glucose in the saliva and gingival fluid, permits the presence of possible more cariogenic bacteria and the development of dental caries.<br><br>Confounding by dietary patters plays an essential role in the in pathway in oral health by this specific group.
	**If Y/PY to 1.1, answer 2.1 and 1.3 to determine whether there is a need to assess time-varying confounding:**		
	**1.2. If Y or PY to 1.1: Was the analysis based on splitting follow up time according to exposure received?**<br><br>**If N or PN to 1.2**, answer questions 1.4 to 1.6, which relate to baseline confounding	NA / Y / PY / PN / **N** / NI	The participants could not switch between exposures, so the outcome could not be biased due time varying confounding.
	**1.3. If Y or PY to 1.2: Were exposure discontinuations or switches likely to be related to factors that are prognostic for the outcome?**	NA / **Y** / PY / PN / N / NI	Severity in metabolic control or duration of the disease suggest an increased risk for a worse DMFT score. In the study no correlation was observed either between duration of diabetes mellitus of the degree of diabetic control as assessed by blood glucose (MGB) or hemoglobin (HbA1) and DMFT score.
	**If N or PN to 1.3**, answer questions 1.4 to 1.6, which relate to baseline confounding		

	**1.4. Did the authors use an appropriate analysis method that adjusted for all the critically important confounding areas?**	NA / Y / PY / PN / **N** / NI	The authors did no adjusted for potential correlation, but calculated the correlation between:<br>- duration of diabetes mellitus of the degree of diabetic control as assessed by blood glucose (MGB) or hemoglobin (HbA1) and DMFT score.<br>- Diabetes complications, i.e. neuropathy, and retinopathy and changes in the DMFT values.<br><br>Thereby a distinction is made for DMFT score, treatment needs according to age groups.
	**1.5. If Y or PY to 1.4: Were confounding areas that were adjusted for measured validly and reliably by the variables available in this study?**	NA / Y / PY / PN / **N** / NI	No adjustment for confounding areas in the study. For example, regression techniques.
	**1.6. Did the authors avoid adjusting for post-exposure variables?**	NA / **Y** / PY / PN / N / NI	Yes, DMFT score is plotted for 7 different age groups:<br>- < 20<br>- 20-29<br>- 30-34<br>- 35-44<br>- 45-54<br>- 55-64<br>- >65
	**If Y or PY to 1.3**, answer questions 1.7 and 1.8, which relate to time-varying confounding		

	**1.7. Did the authors use an appropriate analysis method that adjusted for all the critically important confounding areas and for time-varying confounding?**	NA / Y / PY / PN / **N** / NI	See, above 1.4.

	**1.8. If Y or PY to 1.7: Were confounding areas that were adjusted for measured validly and reliably by the variables available in this study?**	NA / Y / PY / PN / **N** / NI	See above, 1.5
	**Risk of bias judgement**	**Serious**	**The authors did not use any analysis method that adjusted for important confounding areas, only a distinction in age is made.**<br><br>**They have thought about possible correlations for confounding by calculation the correlation between several parameters.**
	Optional: What is the predicted direction of bias due to confounding?	Unpredictable	
Bias in selection of participants into the study	**2.1. Was selection of participants into the study (or into the analysis) based on variables measured after the start of the exposure?**<br><br>**<u>If N or PN to 2.1 go to 2.4</u>**	**Y** / PY / PN / N / NI	Yes<br><br>DM group: based on C-peptide findings, duration of diabetes, mean blood glucose (MGB), HbA1 level, and the presence of diabetic complications, i.e. neuropathy and retinopathy.<br><br>Random sample of 222 dentate diabetic patients (130 males, 92 females), mean age 49.6 years. Patients were selected from the Vuk Vrhovac Institute of Diabetes Endocrinology and Metabolic Diseases, in Zagreb and they had been referred from all parts of Croatia.<br>1. 109 insulin-dependent, type I<br>2. 113 non-insulin-dependent, type II

			Mean duration of diabetes of 11 years.<br><br>No-DM: 189 dentate subjects (115 males, 74 females), mean age 43.9, with no suggestion of diabetes in their medical history. They were taken from the general population of Croatia during a survey on the prevalence of periodontal disease and caries in Croatia.
	**2.2. If Y/PY to 2.1: Were the post-exposure variables that influenced selection associated with exposure?**	Y / PY / PN / N / NI	Yes, it is possible that the severity of diabetes changes over time, but the authors calculated the correlation between the degree of diabetic control and DMFT score, but the specific value is not given.
	**2.3. If Y/PY to 2.2: Were the post-exposure variables that influenced eligibility selection influenced by the outcome or a cause of the outcome?**	NA / Y / PY / PN / N / **NI**	No information about exclusion eligibility.
	**2.4 Do start of follow-up and start of exposure coincide for most participants?**	NA / **Y** / PY / PN / N / NI	Yes, both groups received after the start of the study clinical examination.
	**2.5 If Y/PY to 2.2 and 2.3, or N/PN to 2.4: Were adjustment techniques used that are likely to correct for the presence of selection biases?**	NA / Y / PY / PN / **N** / NI	No adjustment techniques were used to correct for the presence of selection bias.
	**Risk of bias judgement**	**Moderate**	**The sample is representative, but selection into the study may have been related to exposure and outcome.**<br><br>**It is possible that only the sickest diabetics with bad values were included in the exposure group because this group is selected from a specific diabetes institute.**

			**The ''healthy'' diabetics are often not affiliated by these centers.**<br><br>**The authors tried to correct for these limitations because they used a random sample, made distinction in type of diabetes and calculated correlations.**
Bias in classification of exposures	**3.1 Is exposure status well defined?**	**Y** / PY / PN / N / NI	Yes.<br><br>All data were recorded in the WHO combined oral health and treatment needs assessments forms, according to the instructions by the WHO.
	**3.2 Did entry into the study begin with start of the exposure?**	**Y** / PY / PN / N / NI	Yes, the whole random sample of the diabetic participants had at the start of the study diabetes. But there's difference in the level of insulin secretion, expressing in 109 insulin dependent and 113 non-insulin dependent diabetes.
	**3.3 Was information used to define exposure status recorded prior to outcome assessment?**	**Y** / PY / PN / N / NI	Yes, but this is separate and not related to this study.
	**3.4 Could classification of exposure status have been affected by knowledge of the outcome or risk of the outcome?**	Y / PY / PN / N / **NI**	No information
	**3.5 Were exposure assessment methods robust (including methods used to input data)?**	Y / PY / PN / N / **NI**	No information
	**Risk of bias judgement**	**Low**	**Exposure status is well defined; it is not likely that classification of exposure status has been affected by knowledge of the outcome.**
Bias due to departures	**4.1. Is there concern that changes in exposure status occurred among participants?**	**Y** / PY / PN / N / NI	Yes, but the authors made distinctions in insulin-dependent and non-insulin dependent DM and

from intended exposures	**If your aim for this study is to assess the effect of initiating and adhering to an exposure (as in a per-protocol analysis), answer questions 4.2 and 4.3, otherwise continue to 4.4 if Y or PY to 4.1.**		calculated correlations in degree of diabetic control and DMFT score.
	**4.2. Did many participants switch to other exposures?**	**Y** / PY / PN / N / NI	The controls were taken from the general population of Croatia during a survey on the prevalence of periodontal disease and caries in Croatia, with no suggestion of diabetes in their medical history. Despite that it is still possible that undiagnosed diabetics might be in this group and so misclassified as a non-diabetic.
	**4.3. Were the critical co-exposures balanced across exposure groups?**	**Y** / PY / PN / N / NI	Yes, for example age. Mean age in the DM group: 49,6 years and in the control group 43.9 years.<br><br>In many studies there's a big difference in age between those two groups.
	**4.4. If NY/PN PY to 4.1, or Y/PY to 4.2, or 4.3: Were adjustment techniques used that are likely to correct for these issues?**	NA / Y / PY / PN / N / **NI**	No adjustment techniques were used to correct
	**Risk of bias judgement**	**Moderate**	**Any deviations from intended exposure reflected usual practice.**
Bias due to missing data	**5.1 Were there missing outcome data?**	Y / PY / PN / N / **NI**	No specific information about missing outcome data, but all the participants participated at the start of the study (N= 411) were included in data analysis.
	**5.2 Were participants excluded due to missing data on exposure status?**	Y / PY / PN / N / **NI**	No specific information, but it can be assumed that exposure status is well known. They used a random sample from a Diabetes institute.
	**5.3 Were participants excluded due to missing data on other variables needed for the analysis?**	Y / PY / PN / N / **NI**	No information.

	**5.4 <u>If Y/PY to 5.1, 5.2 or 5.3:</u> Are the proportion of participants and reasons for missing data similar across exposures?**	NA / **Y** / PY / PN / N / NI	All the participants participated at the study (N= 411) were included in data analysis.
	**5.5 <u>If Y/PY to 5.1, 5.2 or 5.3:</u> Were appropriate statistical methods used to account for missing data?**	NA / Y / PY / PN / N / **NI**	Not necessary, because the data is complete.
	**Risk of bias judgement**	**Low**	**Not specifically mentioned, but all the participants who participated at the study (N= 411) were included in data analysis. No information about excluding participants due missing data on exposure status is reported.**
Bias in measurement of outcomes	**6.1 Could the outcome measure have been influenced by knowledge of the exposure received?**	**Y** / PY / PN / N / NI	
	**6.2 Was the outcome measure sensitive?**	**Y** / PY / PN / N / NI	Classification for cavities could be sensitive, because only cavities into the dentin were registered. This can be interpreted differently by the two examiners.<br><br>The outcome measurements about missing teeth is a surrogate/hard endpoint and not sensitive for change.
	**6.3 Were outcome assessors unaware of the exposure received by study participants?**	Y / PY / PN / N / **NI**	It is not known if the examiner were blind for exposure status so knowing which patient had diabetes and who had not.
	**6.4 Were the methods of outcome assessment comparable across exposure groups?**	Y / PY / PN / N / NI	Yes, instruments used for examination were a standard probe and a mirror. In both groups, the survey procedures were carried out by the same dentist. Both examiners were calibrated during the survey of oral health in Croatia according to the WHO criteria.

	**6.5 Were any systematic errors in measurement of the outcome unrelated to exposure received?**	**Y** / PY / PN / N / NI	In both groups, the survey procedures were carried out by the same dentist.
	**Risk of bias judgement**	**Low**	**It is unknown if the outcome assessor were unaware for exposure status. If so, it is not likely that this bias the results because tooth loss is not a sensitive outcome.**<br><br>**The methods of outcome assessments were comparable for both groups.**
Bias in selection of	Is the reported effect estimate likely to be selected, on the basis of the results, from...?		
the reported result	**7.1. ... multiple outcome *measurements* within the outcome domain?**	**Y** / PY / PN / N / NI	Yes, complete.
	**7.2 ... multiple *analyses* of the exposure-outcome relationship?**	Y / PY / PN / N / **NI**	No, the specific value about the correlation between:<br>- duration of diabetes mellitus of the degree of diabetic control as assessed by blood glucose (MGB) or hemoglobin (HbA1) and DMFT score.<br>- Diabetes complications, i.e. neuropathy, and retinopathy and changes in the DMFT values;<br>is not specifically given. The authors described this only as no correlation, but how low is this value?
	**7.3 ... different *subgroups*?**	**Y** / PY / PN / N / NI	Yes, complete.

	**Risk of bias judgement**	**Moderate**	**Data were reasonably complete; only specific values of correlations are missing.**
Overall bias	**Risk of bias judgement**	**Serious**	**The sample is representative, and bias due to missing data and measurements of the outcome could be considered as low, but there is no correction for potential confounding (for example by using regression models).**<br><br>**In this cross-sectional design potential confounding should be considered. Only subgroups for age were created and correlation about parameters were calculated. Unfortunately, the specific values of these relationships, which might give a slight indication of potential confounding, have not been reported in the article.**

Online Appendix S2-11

Preliminary tool for risk of bias in exposure study: Falk et al. 1988

Title: Number of teeth, prevalence of caries and periapical lesions in insulin-dependent diabetics

Year: 1989

Authors: HANNE FALK, ANDERS HUGOSON AND HELENE THORSTENSSON

PMID: 2740831

AIM*: The aim of this study was to compare the number of teeth, prevalence of caries and periapical lesions in age and sex-matched adult long and short duration insulin-dependent diabetics and non-diabetics.*

Specify a target experiment specific to the study:

☐ The protocol-specified target experiment fully applies ***OR***

Participant

Experimental exposure

Control exposure

Participant:
The studied population comprised the 741 insulin dependent diabetics, aged 20-70 yr. old examined at the Department of Medicine at the Central Hospital in Jonkoping, Sweden. They constituted
all insulin-dependent diabetics living in the borough of Jonkoping. A sample from this, population was selected for a detailed examination of dental health. From each age group, comprising individuals
born the same year, the man and the woman with the longest and shortest diabetes duration were selected.

The group thus selected consisted of 194 diabetics. Out of these 194 individuals**, 180 took part in the examination,** 94 (48 women and
46 men) with long and 86 (44 women and 42 men) with short diabetes duration.

Experimental/control exposure:
The control group was selected from the county council's register of persons residing in the borough of Jonkoping and consisted of an age and sex-matched, random sample of 102 non-diabetics.
All selected individuals received a written invitation, containing the same information as that given to the diabetics, to attend a dental examination.

Out of the 102 non-diabetics, 86 (49 women and 37 men) took part in the study

Number of individuals examined (20–70 yr old) and diabetes duration. Means ($\bar{x}$)

	Dentate			Edentulous
	Total	Women	Men	Total
Diabetics				
Long duration ($\bar{x}$ = 28.9 yr)	82	42	40	12 (12.8%)
Short duration ($\bar{x}$ = 5.2 yr)	72	34	38	14 (16.3%)
Non-diabetics	77	43	34	9 (10.5%)

Specify the outcome

Specify which outcome is being assessed for risk of bias (typically from among those earmarked for the Summary of Findings table). Specify whether this is a proposed benefit or harm of exposure.

The number of existing teeth (except third molars)

Presence of restoration was recorded for each tooth surface.

Is your aim for this study…?

■ to assess the effect of initiating intervention (as in an intention-to-treat analysis)

☐ to assess the effect of initiating and adhering to intervention (as in a per-protocol analysis)

☐ other (specify)

Specify the numerical result being assessed

In case of multiple alternative analyses being presented, specify the numeric result (e.g. RR = 1.52 (95% CI 0.83 to 2.77) and/or a reference (e.g. to a table, figure or paragraph) that uniquely defines the result being assessed.

Student's /-test was used to determine the significance of differences between two independent groups, Comparisons among more than two
groups were made using analysis of variance (ANOVA), If the ANOVA rejected the multisampling null hypothesis, a multiple comparison procedure

(Newman-Keul test) was used to detect where the differences were located. When frequencies in discrete categories constituted the research<br>data, the chi-square test was used to determine the significance of differences between independent groups. All statistical tests were two tailed and at the 5% significance level. The data are presented as mean values and standard errors.

Preliminary consideration of confounders

Complete a row for each important confounding area (i) listed in the review protocol; and (ii) relevant to the setting of this particular study, or which the study authors identified as potentially important. *"Important" confounding areas are those for which, in the context of this study, adjustment is expected to lead to a clinically important change in the estimated effect of the exposure. "Validity" refers to whether the confounding variable or variables fully measure the area, while "reliability" refers to the precision of the measurement (more measurement error means less reliability).*

**(xix) Confounding areas listed in the review protocol**				
Confounding area	Measured variable(s)	Is there evidence that controlling for this variable was unnecessary?*	Is the confounding area measured validly and reliably by this variable (or these variables)?	OPTIONAL: Is adjusting for this variable (alone) expected to move the effect estimate up or down?
Demographic	Age, sex	Ye	**Yes**	No information
General health status	Duration of diabetes:<br>- Short<br>- Long	Yes	**Yes**	

(xx) **Additional confounding areas relevant to the setting of this particular study, or which the study authors identified as important**

Confounding area	Measured variable(s)	Is there evidence that controlling for this variable was unnecessary?*	Is the confounding area measured validly and reliably by this variable (or these variables)?	OPTIONAL: Is adjusting for this variable (alone) expected to move the effect estimate up or down?
			Yes / No / No information	Favor intervention / Favor control / No information

* In the context of a particular study, variables can be demonstrated not to be confounders and so not included in the analysis: (a) if they are not predictive of the outcome; (b) if they are not predictive of exposure; or (c) because adjustment makes no or minimal difference to the estimated effect of the primary parameter. Note that “no statistically significant association” is not the same as “not predictive”.

Preliminary consideration of criteria used to determine the accuracy of measurement of exposure and outcome

Complete a row for each measure listed in the study for the (i) exposure and (ii) outcome.
Of the measures listed in the protocol, consider the sensitivity, specificity, and confidence in the methods used in the study.

(xix) Exposure measurement method listed in the study		
Method of measurement	Measured exposure	Is the exposure measured validly and reliably by this method (or these methods)?
Medical examination (by physician)		Yes / No / No information

(xx) Outcome measurement method listed in the study		
Method of measurement	Measured outcome	Is the outcome measured validly and reliably by this method (or these methods)?
Dental examin ation		Yes:<br><br>Two dentists carried out the recordings. Before recording, the examiners were calibrated with respect to the diagnostic criteria. A full-mouth intraoral radiography examination was carried out, comprising periapical and posterior bitewing radiographs.<br><br>In edentulous individuals, an orthopantomogram and a bite-plane radiograph were taken in each jaw. The intraoral radiographs were taken with an X-ray unit with a 65 kV tube fitted with a long cone (focus-film distance 20 cm). Paralleling technique was used. The radiographs were examined by one examiner, using a pair of binoculars according to MATTSON (23), without any knowledge of the group to which the individual belonged.
	Number of edentulousness and number of existing teeth	Except third molars

	Caries	Yes:<br><br>Buccal, lingual and occlusal tooth surfaces were examined for caries according to the criteria given by KOCH*<br>1) Initial caries: mineral loss in the enamel surface layer appearing as chalky spots without cavitation in surfaces not previously restored;<br>2) Clinical caries: new carious lesions, on surfaces not previously restored, of such an extent that, on probing, they can be verified as cavities and that, on probing in fissures, the probe sticks at a slight pressure;<br>3) secondary caries: caries lesions according to the criteria of clinical caries but occurring on a previously restored surface;<br>4) proximal caries was only recorded radiographically.<br><br>***KOCH G. Effect of sodium fluoride in dentifrice and mouthwash on incidence of dental caries in schoolchildren. Odontal Revy 1967; 18: Suppl 12.*<br><br>Well-defined decrease of the mineral content on the proximal surface visible on the radiograph was<br>recorded as<br>I) initial caries: mineral loss in the enamel surface layer,<br>2) manifest caries: a caries lesion extending into the dentin,<br>3) secondary caries: a caries lesion according to the criteria of manifest caries but occurring on a restored surface.
	Restorations	Presence of restoration was recorded for each tooth surface.
	Endodontic treatment<br>and periapical status	For each tooth was recorded the presence of:<br>1) an endodontically treated (pulp-amputated or root filled) tooth without a periapical lesion<br>2) an endodontically treated tooth with a periapical or juxtaradicular lesion,<br>3) a tooth not endodontically treated but with a periapical or juxtaradicular lesion.<br><br>A clearly and locally widened periodontal membrane, loss of lamina dura or destruction of bone adjacent to the root was recorded as a lesion, The mean percentage of endodontically treated with and without lesions as well as of non-endodontically treated teeth with lesions was determined.

Preliminary consideration of co-exposures

Complete a row for each important co-intervention (i) listed in the review protocol; and (ii) relevant to the setting of this particular study, or which the study authors identified as important.
"Important" co-interventions are those for which, in the context of this study, adjustment is expected to lead to a clinically important change in the estimated effect of the intervention.

**(xix) Co-exposures listed in the review protocol**		
Co-exposure	Is there evidence that controlling for this co-exposure was unnecessary (e.g., because it was not administered)?	Is presence of this co-exposure likely to favor outcomes in the experimental or the control group
		Favor experimental / Favor comparator / No information
		Favor experimental / Favor comparator / No information
		Favor experimental / Favor comparator / No information

**(xx) Additional co-exposures relevant to the setting of this particular study, or which the study authors identified as important**		
Co-exposure	Is there evidence that controlling for this co-exposure was unnecessary (e.g., because it was not administered)?	Is presence of this co-exposure likely to favor outcomes in the experimental or the control group
		Favor experimental / Favor comparator / No information
		Favor experimental / Favor comparator / No information
		Favor experimental / Favor comparator / No information

Risk of bias assessment (cohort-type studies)

Bias due to confounding	1.1 **Is there potential for confounding of the effect of exposure in this study? If N or PN to 1.1:** the study can be considered to be at low risk of bias due to confounding and no further signaling questions need be considered	**Y** / PY / PN / N	Due the cross-sectional design potential confounders could bias the effect.
	**If Y/PY to 1.1, answer 2.1 and 1.3 to determine whether there is a need to assess time-varying confounding:**		
	**1.2. If Y or PY to 1.1: Was the analysis based on splitting follow up time according to exposure received?** **If N or PN to 1.2, answer questions 1.4 to 1.6, which relate to baseline confounding**	NA / Y / PY / PN / **N** / NI	The participants could not switch between exposures, so the outcome could not be biased due time varying confounding.
	**1.3. If Y or PY to 1.2: Were exposure discontinuations or switches likely to be related to factors that are prognostic for the outcome?**	NA / Y / PY / PN / N / NI	For example, duration of diabetic suggests an increased risk for oral health parameters. The study made a distinction in diabetic duration (but no difference between these groups were found). Also, sex and age difference were taken into the study.
	**If N or PN to 1.3**, answer questions 1.4 to 1.6, which relate to baseline confounding		
	**1.4. Did the authors use an appropriate analysis method that adjusted for all the critically important confounding areas?**	NA / **Y** / PY / PN / N / NI	Yes, matching technique to create an age and sex-matched random sample from the same are.
	**1.5. If Y or PY to 1.4: Were confounding areas that were adjusted for measured validly and reliably by the variables available in this study?**	NA / **Y** / PY / PN / N / NI	Yes, validly and reliably.
	**1.6. Did the authors avoid adjusting for post-exposure variables?**	NA / Y / PY / PN / N / **NI**	No information about adjusting for post-exposure variables.

	**If Y or PY to 1.3**, answer questions 1.7 and 1.8, which relate to time-varying confounding		

	1.7. Did the authors use an appropriate analysis method that adjusted for all the critically important confounding areas and for time-varying confounding?	NA / **Y** / PY / PN / N / NI	Yes, matching technique to create an age and sex-matched random sample from the same are.
	1.8. **If Y or PY to 1.7**: Were confounding areas that were adjusted for measured validly and reliably by the variables available in this study?	NA / Y / PY / PN / N / NI	Yes, validly and reliably.
	**Risk of bias judgement**	**Moderate**	**Potential confounding, but the analysis was performed in different age groups and diabetes duration and thereafter adjusted for sex.**
Bias in selection of participants into the study	**2.1. Was selection of participants into the study (or into the analysis) based on variables measured after the start of the exposure?**<br><br>**If N or PN to 2.1 go to 2.4**	Y / PY / PN / **N** / NI	No, selection of participants took part prior at the start of the study.<br><br>DM:<br>selected from the Department of medicine at the central Hospital in Jönköping, Sweden<br><br>No-DM:<br>selected from the county council's register of persons residing in the borough of Jonkoping.
	**2.2. If Y/PY to 2.1: Were the post-exposure variables that influenced selection associated with exposure?**	Y / PY / PN / N / NI	Yes, it is possible that the severity of diabetes changes over time. Because of the cross-sectional design, there were no detailed information glycemic control, only a distinction is made between long and short duration of diabetes.

	**2.3. <u>If Y/PY to 2.2:</u> Were the post-exposure variables that influenced eligibility selection influenced by the outcome or a cause of the outcome?**	NA / Y / PY / PN / N / **NI**	No information about exclusion eligibility.
	**2.4 Do start of follow-up and start of exposure coincide for most participants?**	NA / Y / PY / PN / N / NI	Yes, both groups received after the start of the study clinical examination by an dentist.
	**2.5 If Y/PY to 2.2 and 2.3, or N/PN to 2.4: Were adjustment techniques used that are likely to correct for the presence of selection biases?**	NA / Y / PY / PN / N / NI	No adjustment techniques were used to correct for the presence of selection bias.
	**Risk of bias judgement**	**Low**	**The participants in the study constituted a well-defined, group of diabetics; a sample of all insulin-dependent diabetics in the borough of Jönköping.**<br><br>**The control group consisted of an age and sex-matched random sample of non-diabetics from the same area.**<br><br>**The sample in the study is well defined and representative.**
Bias in classification of exposures	**3.1 Is exposure status well defined?**	Y / **PY** / PN / N / NI	No detailed description about the classification of diabetes. The studies population examined at the Department of Medicine at the Central Hospital in Jonkoping, Sweden.<br><br>It can be assumed that classification and thereafter diabetes duration is clinically assessed and done in a scientific way, because the data was used from a central hospital. For example, using the WHO classification or ADA guidelines.

	**3.2 Did entry into the study begin with start of the exposure?**	**Y** / PY / PN / N / NI	Yes, because of the cross-sectional design the entire diabetic group is exposed at the start of the study.<br><br>There is a difference in diabetes duration.
	**3.3 Was information used to define exposure status recorded prior to outcome assessment?**	Y / PY / PN / N / **NI**	Yes, but this is separate and not related to this study. Only insulin dependent diabetics were included.
	**3.4 Could classification of exposure status have been affected by knowledge of the outcome or risk of the outcome?**	Y / PY / PN / **N** / NI	No, classification of exposure is prior at the start of the study because the data is received elsewhere.
	**3.5 Were exposure assessment methods robust (including methods used to input data)?**	Y / PY / PN / N / **NI**	No information.
	**Risk of bias judgement**	**Moderate**	**It is not clear whether allocation for DM/controls are based on (for example WHO criteria, HbA1c levels).**<br><br>**It is not likely that classification of exposure status has not been affected by knowledge of the outcome.**
Bias due to departures from intended exposures	**4.1. Is there concern that changes in exposure status occurred among participants?**<br><br>**If your aim for this study is to assess the effect of initiating and adhering to an exposure (as in a per-protocol analysis), answer questions 4.2 and 4.3, otherwise continue to 4.4 if Y or PY to 4.1.**	Y / PY / PN / N / NI	Yes, but the authors made a distinction for diabetes. Only focused on DM type I and duration of the disease.
	**4.2. Did many participants switch to other exposures?**	Y / PY / PN / N / NI	It is possible that undiagnosed have been misclassified in the non-diabetics group.

	**4.3. Were the critical co-exposures balanced across exposure groups?**	Y / PY / PN / N / NI	Yes, the authors used matching methods to create a age and sex-matched sample from the same area.
	4.4. **If NY/PN PY to 4.1, or Y/PY to 4.2, or 4.3:** Were adjustment techniques used that are likely to correct for these issues?	NA / Y / PY / PN / N / NI	Not necessary.
	**Risk of bias judgement**	**Moderate**	**Any deviations from intended exposure reflected usual practice. Due the cross-sectional design it is not possible to analyse changes in exposure status, because assessment of the exposure is measured one point at the time.**
Bias due to missing data	**5.1 Were there missing outcome data?**	Y / PY / PN / N / NI	A detailed description of the material and non-respondents has been given in a previous report*<br><br>* *Hugoson, A., Thorstensson, H., Faltt, H., & Kuylenstierna, J. (1989). Periodontal conditions in insulin-dependent diabetics. Journal of clinical periodontology, 16(4), 215-223.*
	**5.2 Were participants excluded due to missing data on exposure status?**	Y / PY / PN / N / NI	No.
	**5.3 Were participants excluded due to missing data on other variables needed for the analysis?**	Y / PY / PN / N / NI	Yes: reported in the previous report:<br><br>Out of the 14 diabetics (7.2%) who did not participate in the study, one reported edentulousness in the upper jaw. Radiographs from general practitioners showed that one diabetic exhibited advanced and 2 minor periodontal breakdowns. Out of the 16 non-diabetics (15.7%) who did not respond, 2 exhibited minor

			periodontal breakdown as assessed from radiographs obtained from their general practitioners. Many different reasons were given for not taking part in the study.
	**5.4 If Y/PY to 5.1, 5.2 or 5.3: Are the proportion of participants and reasons for missing data similar across exposures?**	NA / Y / PY / PN / N / NI	Yes, fourteen diabetics (7.2%) and 16 non-diabetics (15.7%) did not participate in the study.
	**5.5 If Y/PY to 5.1, 5.2 or 5.3: Were appropriate statistical methods used to account for missing data?**	NA / Y / PY / PN / **N** / NI	No not necessary, people with missing data did not participate in the study and were excluded for any further analysis.
	**Risk of bias judgement**	**Low**	**Data is complete and well described.**
Bias in measurement of outcomes	**6.1 Could the outcome measure have been influenced by knowledge of the exposure received?**	Y / PY / PN / **N** / NI	No.
	**6.2 Was the outcome measure sensitive?**	**Y** / PY / PN / N / NI	It is well known that recording of caries and assessment of periapical conditions show great inter- and intra-examiner variability, but the authors performed analysis of observation errors. The outcome measurements about missing teeth is a surrogate/hard endpoint and not sensitive for change.
	**6.3 Were outcome assessors unaware of the exposure received by study participants?**	**Y** / PY / PN / N / NI	Two dentists carried out the recordings without any knowledge of the group to which the individual belonged.
	**6.4 Were the methods of outcome assessment comparable across exposure groups?**	**Y /** PY / PN / N / NI	Yes, the examiners were calibrated with respect to the diagnostic criteria and a similar full-mouth intraoral radiographic examination was carried out for both groups.
	**6.5 Were any systematic errors in measurement of the outcome unrelated to exposure received?**	**Y** / PY / PN / N / NI	Yes, the examiners were calibrated with respect to the diagnostic criteria.

			Thereby the authors performed a random sample of 20 full-mouth intraoral radiographs and examined twice with regard to caries and periapical status by the examiner who carried out the radiographic analysis.<br>The reliability coefficient and error variance is calculated to compare potential systematic errors.
	**Risk of bias judgement**	**Low**	**The methods of outcome assessment were comparable across exposure groups. The analysis of observation errors indicated good reliability.**
Bias in selection of	Is the reported effect estimate likely to be selected, on the basis of the results, from...?		
the reported result	**7.1. ... multiple outcome *measurements* within the outcome domain?**	**Y** / PY / PN / N / NI	Yes, all the outcome measurements about number of teeth, carious lesions, restoration, endodontically treated teeth and periapical lesions were fully reported.
	**7.2 ... multiple *analyses* of the exposure-outcome relationship?**	Y / PY / PN / N / **NI**	Not applicable.
	**7.3 ... different *subgroups*?**	**Y** / PY / PN / N / NI	Yes, distinction is made in diabetic duration.
	**Risk of bias judgement**	**Low**	**All the data were fully described.**
Overall bias	**Risk of bias judgement**	**Moderate**	**The sample is only representative for type I diabetes. The authors used an age and sex matched random sample from the same area which makes the analysis comparable. Not all the confounding areas were fully described and adjusted.**

Online Appendix S3-1

Meta-analysis, subgroup analysis: forest plots using a random model (REM) and fixed model (FEM) of the performed meta-analysis for the DM patients compared to non-DM on risk of bias.

Low risk of bias (FEM)

	DM		no DM			Risk Ratio
Study or Subgroup	Events	Total	Events	Total	Weight	M–H, Fixed, 95% CI
2.1.1 28 teeth						
Botero et al. 2012	481	1820	112	1652	0.0%	3.90 [3.21, 4.74]
Costa et al. 2011/2013	225	1288	183	1288	0.0%	1.23 [1.03, 1.47]
Falk et al. 1988	1007	4312	416	2156	0.0%	1.21 [1.09, 1.34]
Greenblatt et al. 2016	10140	78176	20733	181412	74.4%	1.13 [1.11, 1.16]
Kaur et al. 2009	3414	9156	28218	110908	25.6%	1.47 [1.42, 1.51]
Patel et al. 2013	3763	10752	11196	46788	0.0%	1.46 [1.42, 1.51]
Patino–Márin et al. 2008	200	980	123	980	0.0%	1.63 [1.32, 2.00]
Shin et al. 2017	7356	36260	26331	303408	0.0%	2.34 [2.28, 2.39]
Subtotal (95% CI)		87332		292320	100.0%	1.22 [1.20, 1.24]
Total events	13554		48951			
Heterogeneity: Chi² = 200.84, df = 1 (P < 0.00001); I² = 100%						
Test for overall effect: Z = 21.83 (P < 0.00001)						
2.1.2 32 teeth						
Backr et al. 1989	2731	7104	1833	6048	0.0%	1.27 [1.21, 1.33]
Sensorn et al. 2012	2414	12128	694	7232	0.0%	2.07 [1.92, 2.25]
Subtotal (95% CI)		0		0		Not estimable
Total events	0		0			
Heterogeneity: Not applicable						
Test for overall effect: Not applicable						
Total (95% CI)		87332		292320	100.0%	1.22 [1.20, 1.24]
Total events	13554		48951			
Heterogeneity: Chi² = 200.84, df = 1 (P < 0.00001); I² = 100%						
Test for overall effect: Z = 21.83 (P < 0.00001)						
Test for subgroup differences: Not applicable						

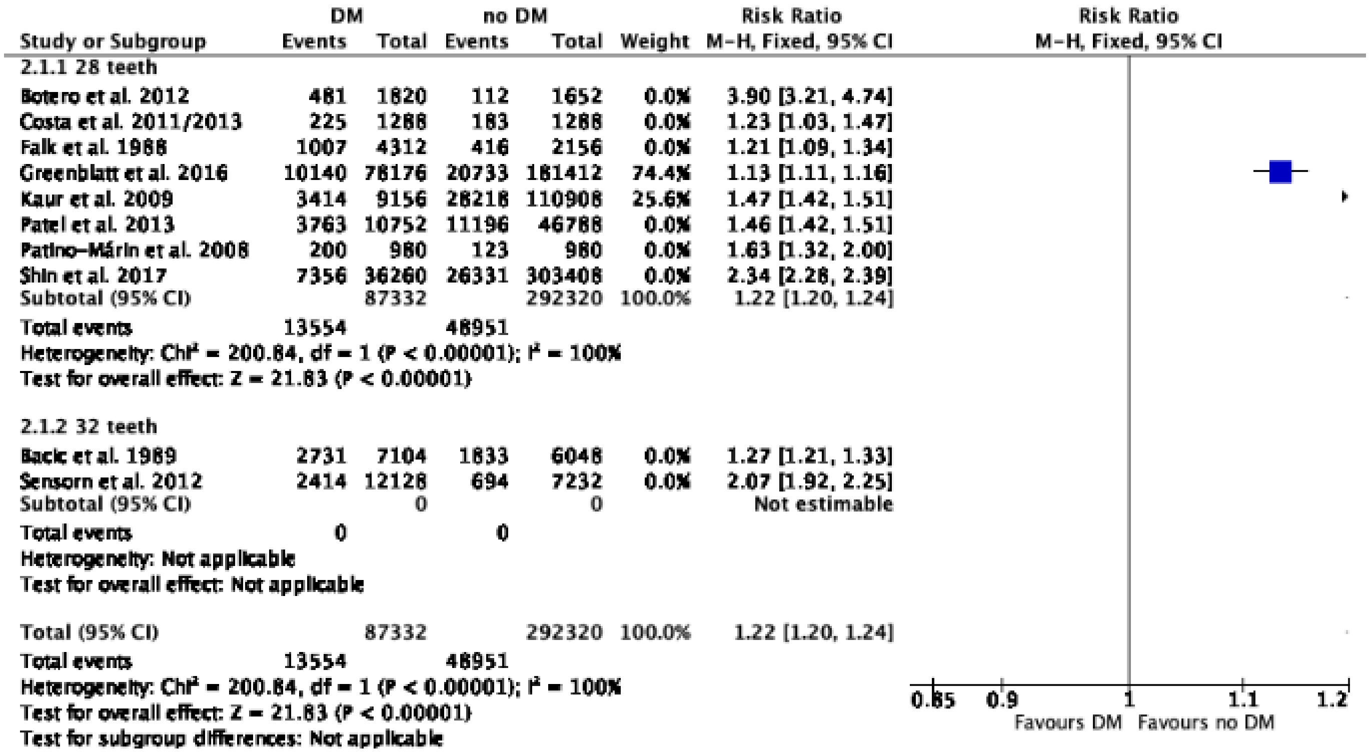

Moderate risk of bias (REM)

Study or Subgroup	DM Events	DM Total	no DM Events	no DM Total	Weight	Risk Ratio M-H, Random, 95% CI
2.1.1 28 teeth						
Botero et al. 2012	481	1820	112	1652	19.6%	3.90 [3.21, 4.74]
Costa et al. 2011/2013	225	1288	183	1288	19.8%	1.23 [1.03, 1.47]
Falk et al. 1988	1007	4312	416	2156	20.4%	1.21 [1.09, 1.34]
Greenblatt et al. 2016	10140	78176	20733	181412	0.0%	1.13 [1.11, 1.16]
Kaur et al. 2009	3414	9156	28218	110908	0.0%	1.47 [1.42, 1.51]
Patel et al. 2013	3763	10752	11196	46788	0.0%	1.46 [1.42, 1.51]
Patino-Márin et al. 2008	200	980	123	980	19.5%	1.63 [1.32, 2.00]
Shin et al. 2017	7356	36260	26331	303408	20.7%	2.34 [2.28, 2.39]
Subtotal (95% CI)		44660		309484	100.0%	1.85 [1.27, 2.71]
Total events	9269		27165			
Heterogeneity: $Tau^2 = 0.18$; $Chi^2 = 237.06$, df = 4 ($P < 0.00001$); $I^2 = 98\%$						
Test for overall effect: Z = 3.18 (P = 0.001)						
2.1.2 32 teeth						
Backi et al. 1989	2731	7104	1833	6048	0.0%	1.27 [1.21, 1.33]
Sensorn et al. 2012	2414	12128	694	7232	0.0%	2.07 [1.92, 2.25]
Subtotal (95% CI)		0		0		Not estimable
Total events	0		0			
Heterogeneity: Not applicable						
Test for overall effect: Not applicable						
Total (95% CI)		44660		309484	100.0%	1.85 [1.27, 2.71]
Total events	9269		27165			
Heterogeneity: $Tau^2 = 0.18$; $Chi^2 = 237.06$, df = 4 ($P < 0.00001$); $I^2 = 98\%$						
Test for overall effect: Z = 3.18 (P = 0.001)						
Test for subgroup differences: Not applicable						

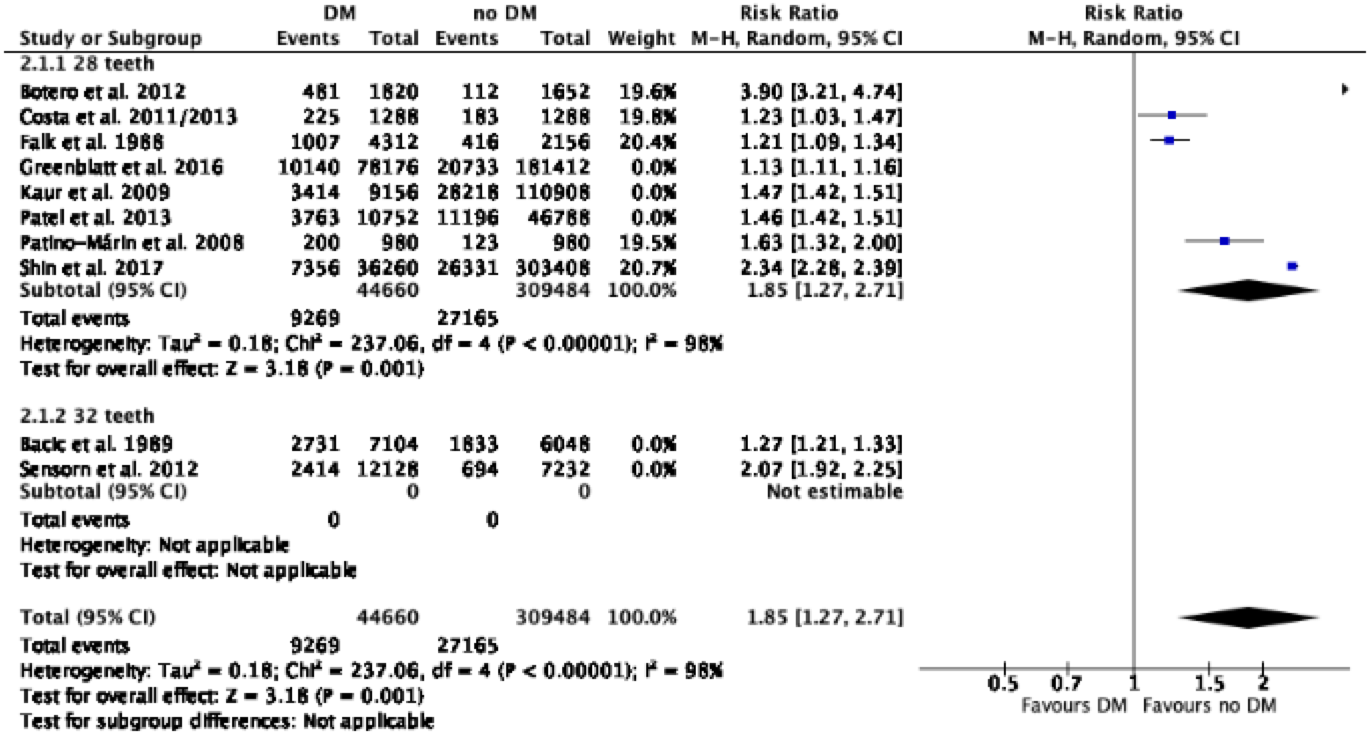

Serious risk of bias (FEM)

Study or Subgroup	DM Events	DM Total	no DM Events	no DM Total	Weight	Risk Ratio M-H, Fixed, 95% CI
2.1.1 28 teeth						
Botero et al. 2012	481	1820	112	1652	0.0%	3.90 [3.21, 4.74]
Costa et al. 2011/2013	225	1288	183	1288	0.0%	1.23 [1.03, 1.47]
Falk et al. 1988	1007	4312	416	2156	0.0%	1.21 [1.09, 1.34]
Greenblatt et al. 2016	10140	78176	20733	181412	0.0%	1.13 [1.11, 1.16]
Kaur et al. 2009	3414	9156	28218	110908	0.0%	1.47 [1.42, 1.51]
Patel et al. 2013	3763	10752	11196	46788	59.5%	1.46 [1.42, 1.51]
Patino–Márin et al. 2008	200	980	123	980	0.0%	1.63 [1.32, 2.00]
Shin et al. 2017	7356	36260	26331	303408	0.0%	2.34 [2.28, 2.39]
Subtotal (95% CI)		10752		46788	59.5%	1.46 [1.42, 1.51]
Total events	3763		11196			
Heterogeneity: Not applicable						
Test for overall effect: Z = 24.51 (P < 0.00001)						
2.1.2 32 teeth						
Bacic et al. 1989	2731	7104	1833	6048	28.2%	1.27 [1.21, 1.33]
Sensorn et al. 2012	2414	12128	694	7232	12.4%	2.07 [1.92, 2.25]
Subtotal (95% CI)		19232		13280	40.5%	1.51 [1.45, 1.58]
Total events	5145		2527			
Heterogeneity: $Chi^2 = 112.39$, df = 1 ($P < 0.00001$); $I^2 = 99\%$						
Test for overall effect: Z = 19.49 (P < 0.00001)						
Total (95% CI)		29984		60068	100.0%	1.48 [1.45, 1.52]
Total events	8908		13723			
Heterogeneity: $Chi^2 = 110.08$, df = 2 ($P < 0.00001$); $I^2 = 98\%$						
Test for overall effect: Z = 31.19 (P < 0.00001)						
Test for subgroup differences: $Chi^2 = 1.74$, df = 1 ($P = 0.19$), $I^2 = 42.5\%$						

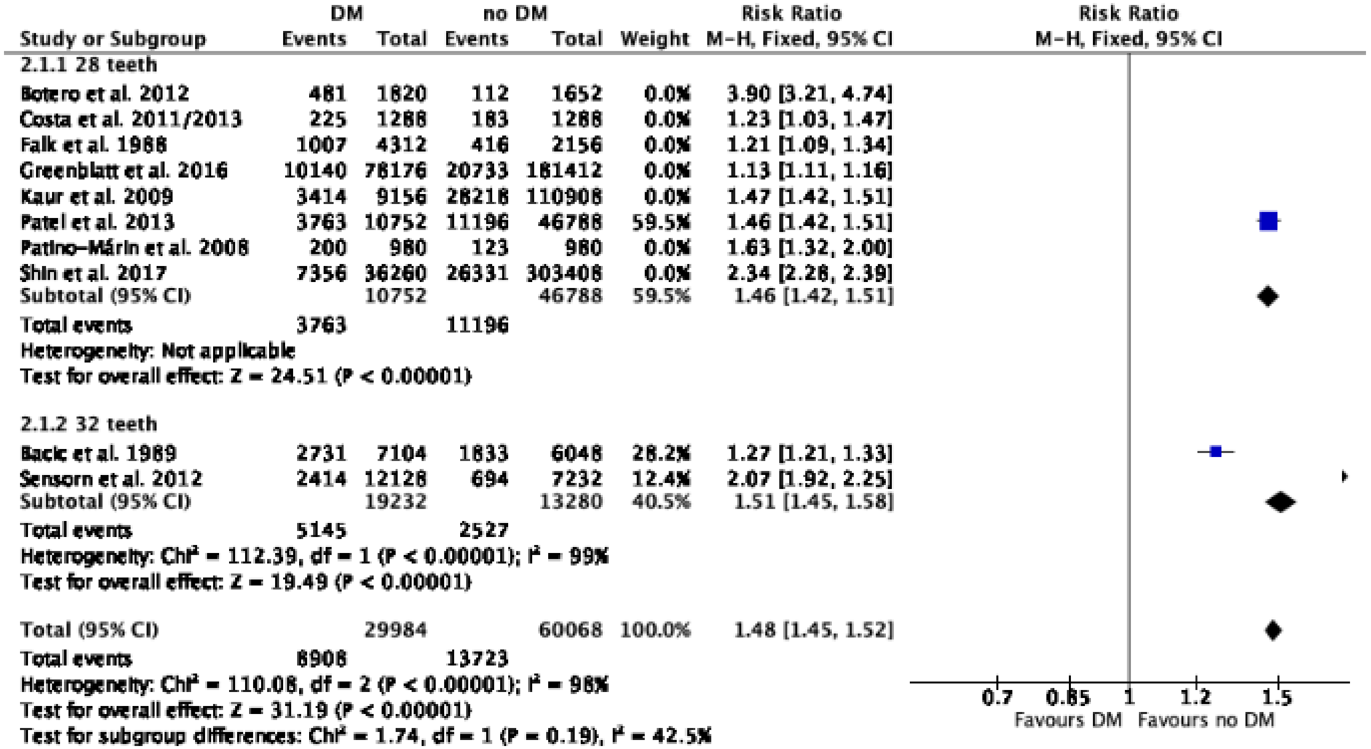

Online Appendix S3-2

Meta-analysis, subgroup analysis: forest plots using a random model of the performed meta-analysis for the DM patients compared to non-DM on cross-sectional study design.

	DM		no DM			Risk Ratio
Study or Subgroup	Events	Total	Events	Total	Weight	M-H, Random, 95% CI
2.1.1 28 teeth						
Botero et al. 2012	481	1820	112	1652	11.6%	3.90 [3.21, 4.74]
Costa et al. 2011/2013	225	1288	183	1288	0.0%	1.23 [1.03, 1.47]
Falk et al. 1988	1007	4312	416	2156	12.6%	1.21 [1.09, 1.34]
Greenblatt et al. 2016	10140	78176	20733	181412	0.0%	1.13 [1.11, 1.16]
Kaur et al. 2009	3414	9156	28218	110908	12.9%	1.47 [1.42, 1.51]
Patel et al. 2013	3763	10752	11196	46788	12.9%	1.46 [1.42, 1.51]
Patino-Márin et al. 2008	200	980	123	980	11.5%	1.63 [1.32, 2.00]
Shin et al. 2017	7356	36260	26331	303408	12.9%	2.34 [2.28, 2.39]
Subtotal (95% CI)		63280		465892	74.4%	1.82 [1.43, 2.32]
Total events	16221		66396			
Heterogeneity: $Tau^2 = 0.09$; $Chi^2 = 978.08$, df = 5 ($P < 0.00001$); $I^2 = 99\%$						
Test for overall effect: Z = 4.85 ($P < 0.00001$)						
2.1.2 32 teeth						
Back et al. 1989	2731	7104	1833	6048	12.9%	1.27 [1.21, 1.33]
Sensorn et al. 2012	2414	12128	694	7232	12.7%	2.07 [1.92, 2.25]
Subtotal (95% CI)		19232		13280	25.6%	1.62 [0.99, 2.65]
Total events	5145		2527			
Heterogeneity: $Tau^2 = 0.12$; $Chi^2 = 112.39$, df = 1 ($P < 0.00001$); $I^2 = 99\%$						
Test for overall effect: Z = 1.92 ($P = 0.05$)						
Total (95% CI)		82512		479172	100.0%	1.77 [1.44, 2.17]
Total events	21366		68923			
Heterogeneity: $Tau^2 = 0.08$; $Chi^2 = 1175.01$, df = 7 ($P < 0.00001$); $I^2 = 99\%$						
Test for overall effect: Z = 5.44 ($P < 0.00001$)						
Test for subgroup differences: $Chi^2 = 0.17$, df = 1 ($P = 0.68$), $I^2 = 0\%$						

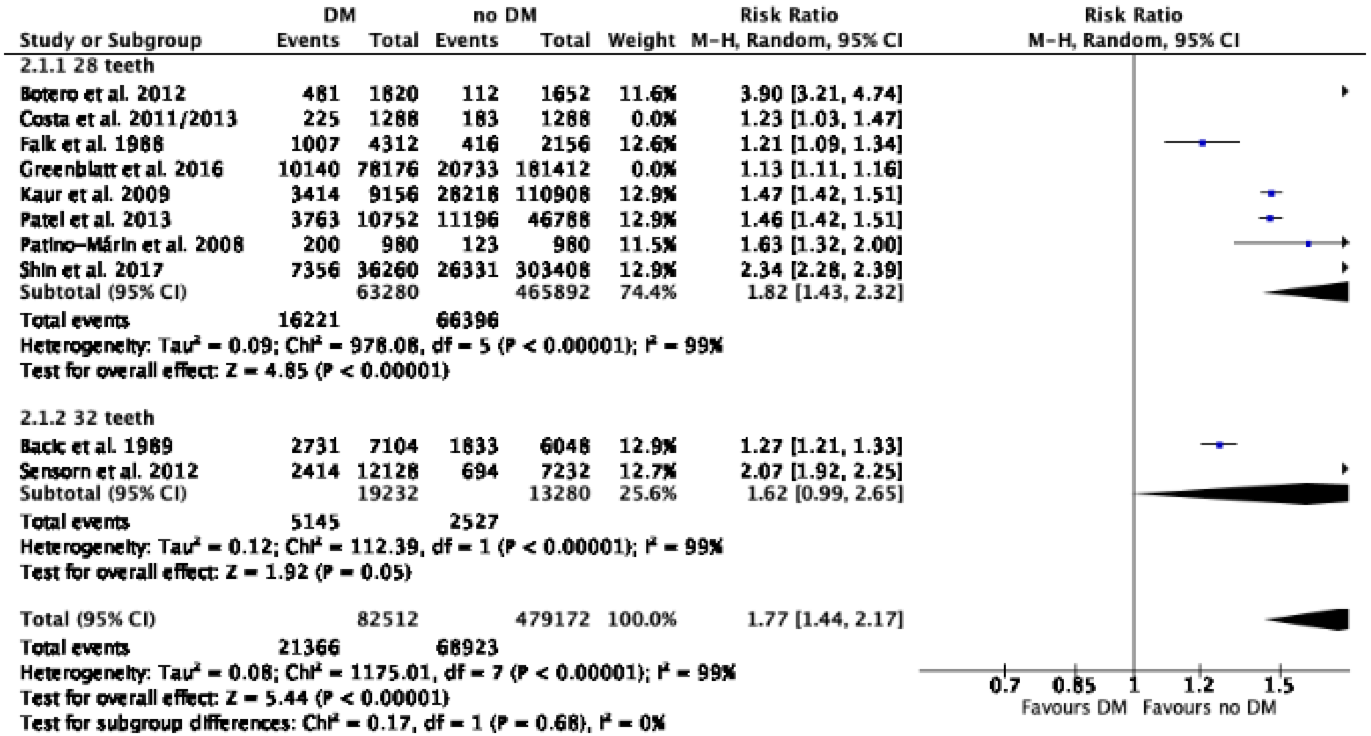

Online Appendix S3-3

Meta-analysis, subgroup analysis: forest plots using a random model and fixed model of the performed meta-analysis for the DM patients compared to non-DM on continent.

Europe

Study or Subgroup	DM Events	DM Total	no DM Events	no DM Total	Weight	Risk Ratio M-H, Fixed, 95% CI
2.1.1 28 teeth						
Botero et al. 2012	481	1820	112	1652	0.0%	3.90 [3.21, 4.74]
Costa et al. 2011/2013	225	1288	183	1288	0.0%	1.23 [1.03, 1.47]
Falk et al. 1988	1007	4312	416	2156	8.1%	1.21 [1.09, 1.34]
Greenblatt et al. 2016	10140	78176	20733	181412	0.0%	1.13 [1.11, 1.16]
Kaur et al. 2009	3414	9156	28218	110908	62.9%	1.47 [1.42, 1.51]
Patel et al. 2013	3763	10752	11196	46788	0.0%	1.46 [1.42, 1.51]
Patino-Márin et al. 2008	200	980	123	980	0.0%	1.63 [1.32, 2.00]
Shin et al. 2017	7356	36260	26331	303408	0.0%	2.34 [2.28, 2.39]
Subtotal (95% CI)		13468		113064	71.0%	1.44 [1.40, 1.48]
Total events	4421		28634			
Heterogeneity: Chi² = 12.77, df = 1 (P = 0.0004); I² = 92%						
Test for overall effect: Z = 25.74 (P < 0.00001)						
2.1.2 32 teeth						
Back et al. 1989	2731	7104	1833	6048	29.0%	1.27 [1.21, 1.33]
Sensorn et al. 2012	2414	12128	694	7232	0.0%	2.07 [1.92, 2.25]
Subtotal (95% CI)		7104		6048	29.0%	1.27 [1.21, 1.33]
Total events	2731		1833			
Heterogeneity: Not applicable						
Test for overall effect: Z = 9.66 (P < 0.00001)						
Total (95% CI)		20572		119112	100.0%	1.39 [1.35, 1.42]
Total events	7152		30467			
Heterogeneity: Chi² = 34.42, df = 2 (P < 0.00001); I² = 94%						
Test for overall effect: Z = 26.77 (P < 0.00001)						
Test for subgroup differences: Chi² = 19.25, df = 1 (P < 0.0001), I² = 94.8%						

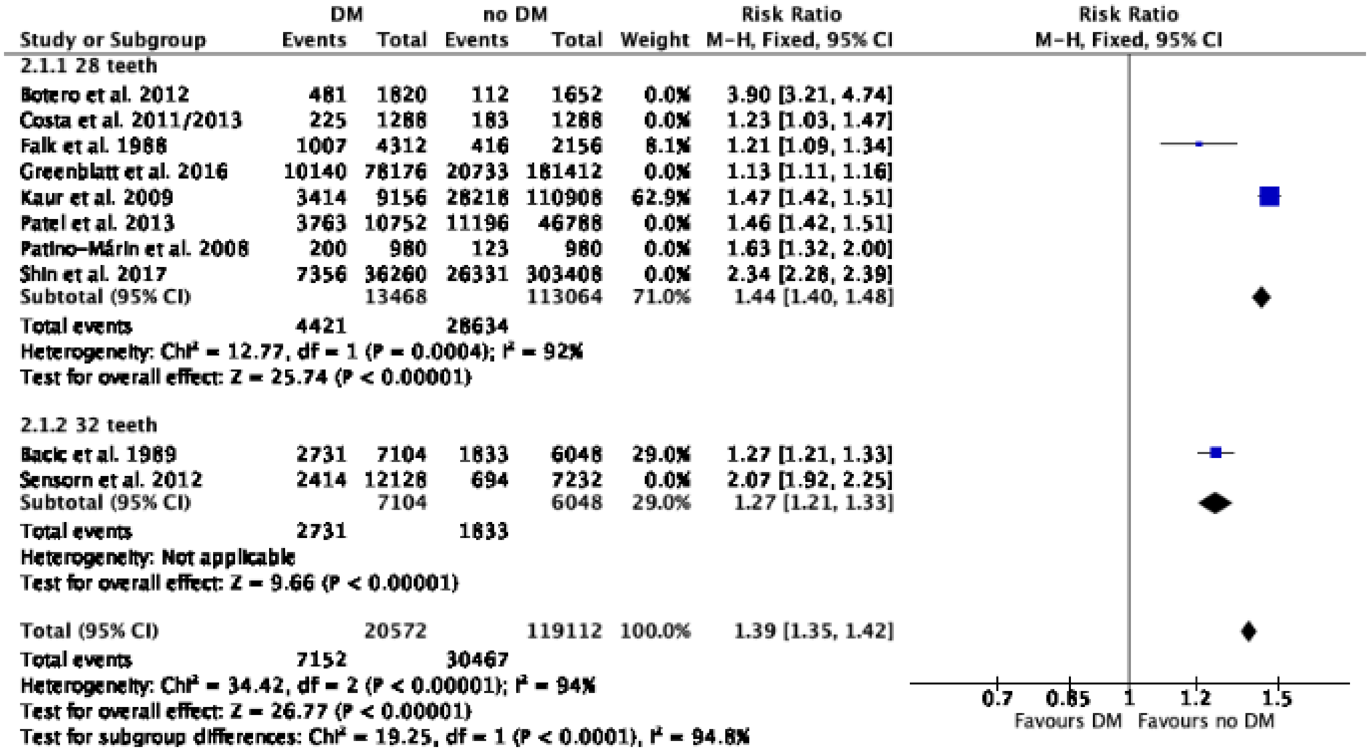

North-America

	DM		no DM			Risk Ratio
Study or Subgroup	Events	Total	Events	Total	Weight	M-H, Fixed, 95% CI
2.1.1 28 teeth						
Botero et al. 2012	481	1820	112	1652	0.0%	3.90 [3.21, 4.74]
Costa et al. 2011/2013	225	1288	183	1288	0.0%	1.23 [1.03, 1.47]
Falk et al. 1988	1007	4312	416	2156	0.0%	1.21 [1.09, 1.34]
Greenblatt et al. 2016	10140	78176	20733	181412	74.4%	1.13 [1.11, 1.16]
Kaur et al. 2009	3414	9156	28218	110908	0.0%	1.47 [1.42, 1.51]
Patel et al. 2013	3763	10752	11196	46788	24.9%	1.46 [1.42, 1.51]
Patino-Márin et al. 2008	200	980	123	980	0.7%	1.63 [1.32, 2.00]
Shin et al. 2017	7356	36260	26331	303408	0.0%	2.34 [2.28, 2.39]
Subtotal (95% CI)		89908		229180	100.0%	1.22 [1.20, 1.24]
Total events	14103		32052			
Heterogeneity: $Chi^2 = 184.67$, df = 2 ($P < 0.00001$); $I^2 = 99\%$						
Test for overall effect: Z = 21.61 ($P < 0.00001$)						
2.1.2 32 teeth						
Back et al. 1989	2731	7104	1833	6048	0.0%	1.27 [1.21, 1.33]
Sensorn et al. 2012	2414	12128	694	7232	0.0%	2.07 [1.92, 2.25]
Subtotal (95% CI)		0		0		Not estimable
Total events	0		0			
Heterogeneity: Not applicable						
Test for overall effect: Not applicable						
Total (95% CI)		89908		229180	100.0%	1.22 [1.20, 1.24]
Total events	14103		32052			
Heterogeneity: $Chi^2 = 184.67$, df = 2 ($P < 0.00001$); $I^2 = 99\%$						
Test for overall effect: Z = 21.61 ($P < 0.00001$)						
Test for subgroup differences: Not applicable						

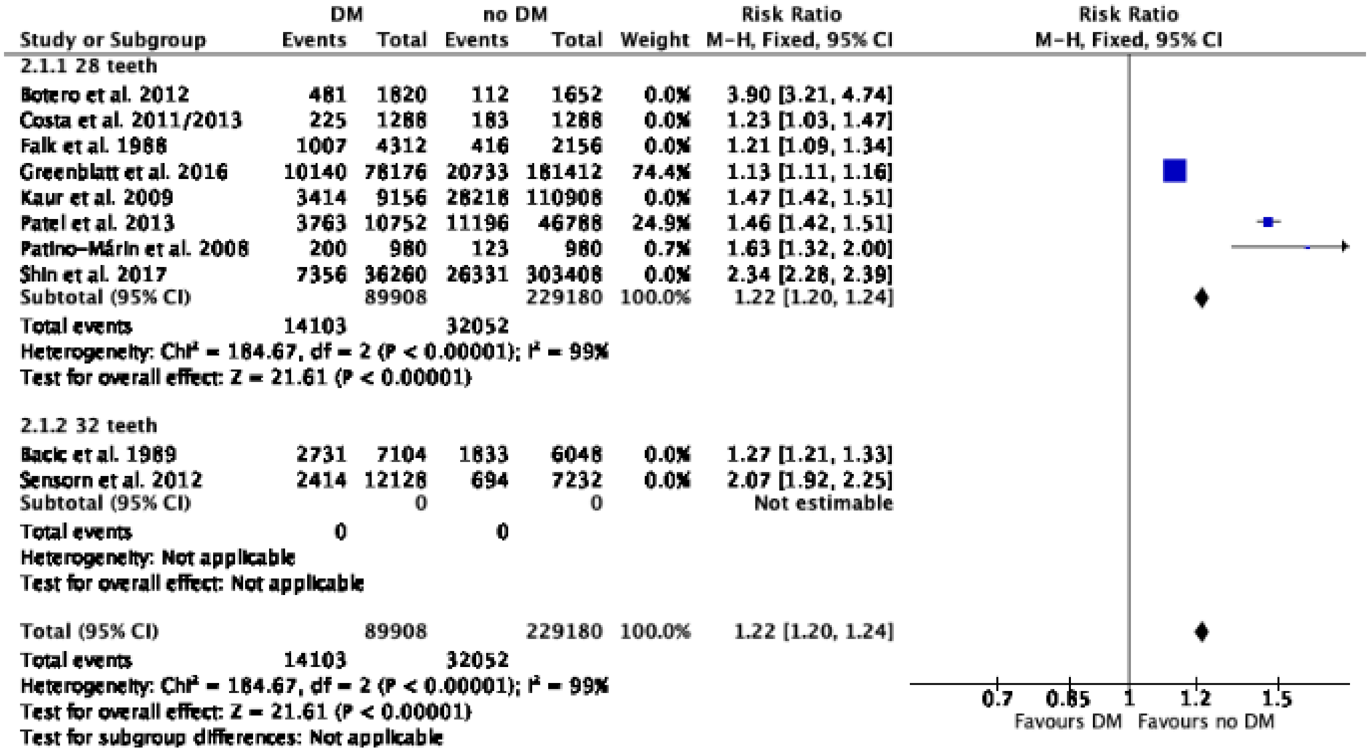

Asia

Study or Subgroup	DM Events	DM Total	no DM Events	no DM Total	Weight	Risk Ratio M-H, Fixed, 95% CI
2.1.1 28 teeth						
Botero et al. 2012	481	1820	112	1652	0.0%	3.90 [3.21, 4.74]
Costa et al. 2011/2013	225	1288	183	1288	0.0%	1.23 [1.03, 1.47]
Falk et al. 1988	1007	4312	416	2156	0.0%	1.21 [1.09, 1.34]
Greenblatt et al. 2016	10140	78176	20733	181412	0.0%	1.13 [1.11, 1.16]
Kaur et al. 2009	3414	9156	28218	110908	0.0%	1.47 [1.42, 1.51]
Patel et al. 2013	3763	10752	11196	46788	0.0%	1.46 [1.42, 1.51]
Patino-Márin et al. 2008	200	980	123	980	0.0%	1.63 [1.32, 2.00]
Shin et al. 2017	7356	36260	26331	303408	86.6%	2.34 [2.28, 2.39]
Subtotal (95% CI)		36260		303408	86.6%	2.34 [2.28, 2.39]
Total events	7356		26331			
Heterogeneity: Not applicable						
Test for overall effect: Z = 71.00 (P < 0.00001)						
2.1.2 32 teeth						
Backe et al. 1989	2731	7104	1833	6048	0.0%	1.27 [1.21, 1.33]
Sensorn et al. 2012	2414	12128	694	7232	13.4%	2.07 [1.92, 2.25]
Subtotal (95% CI)		12128		7232	13.4%	2.07 [1.92, 2.25]
Total events	2414		694			
Heterogeneity: Not applicable						
Test for overall effect: Z = 18.05 (P < 0.00001)						
Total (95% CI)		48388		310640	100.0%	2.30 [2.25, 2.36]
Total events	9770		27025			
Heterogeneity: Chi^2 = 8.28, df = 1 (P = 0.004); I^2 = 88%						
Test for overall effect: Z = 71.68 (P < 0.00001)						
Test for subgroup differences: Chi^2 = 8.04, df = 1 (P = 0.005), I^2 = 87.6%						

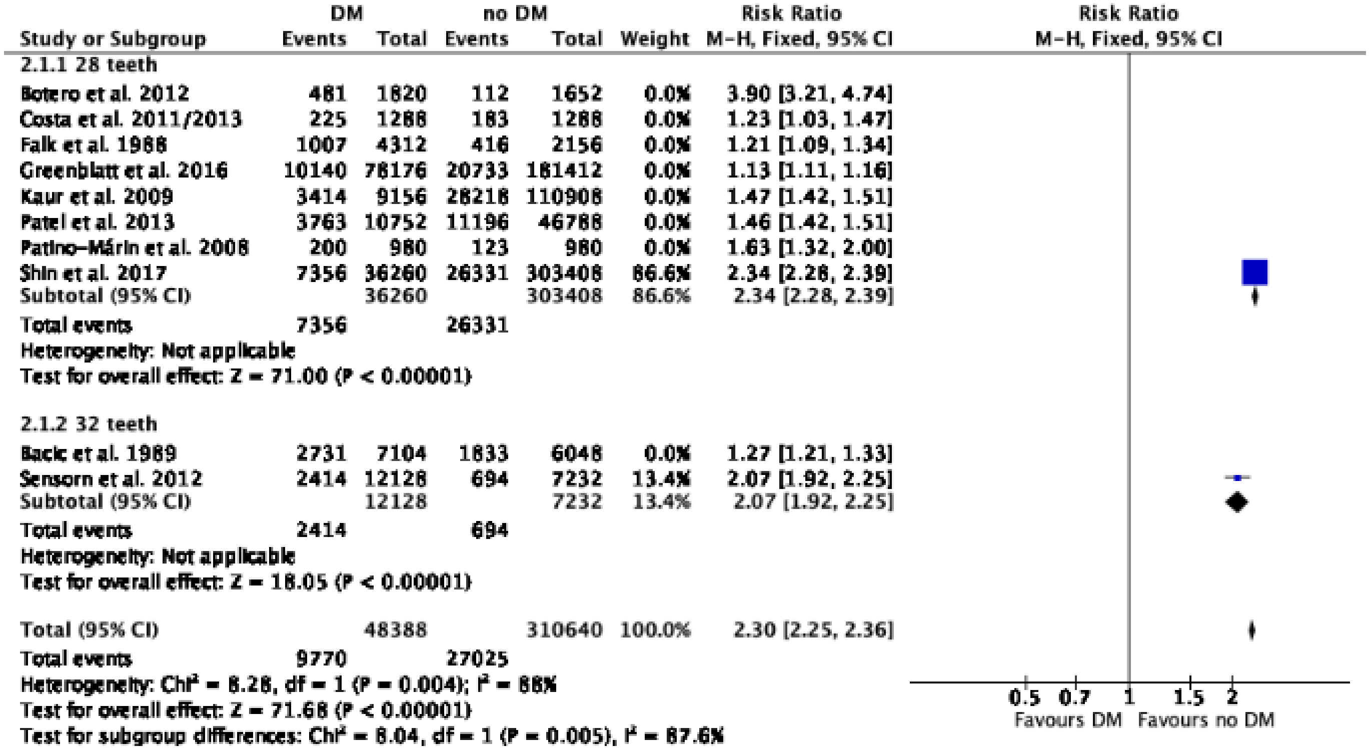

South America

Study or Subgroup	DM Events	DM Total	no DM Events	no DM Total	Weight	Risk Ratio M-H, Fixed, 95% CI
2.1.1 28 teeth						
Botero et al. 2012	481	1820	112	1652	39.1%	3.90 [3.21, 4.74]
Costa et al. 2011/2013	225	1288	183	1288	60.9%	1.23 [1.03, 1.47]
Falk et al. 1988	1007	4312	416	2156	0.0%	1.21 [1.09, 1.34]
Greenblatt et al. 2016	10140	78176	20733	181412	0.0%	1.13 [1.11, 1.16]
Kaur et al. 2009	3414	9156	28218	110908	0.0%	1.47 [1.42, 1.51]
Patel et al. 2013	3763	10752	11196	46788	0.0%	1.46 [1.42, 1.51]
Patino-Márin et al. 2008	200	980	123	980	0.0%	1.63 [1.32, 2.00]
Shin et al. 2017	7356	36260	26331	303408	0.0%	2.34 [2.28, 2.39]
Subtotal (95% CI)		3108		2940	100.0%	2.27 [2.00, 2.58]
Total events	706		295			
Heterogeneity: Chi² = 74.71, df = 1 (P < 0.00001); I² = 99%						
Test for overall effect: Z = 12.58 (P < 0.00001)						
2.1.2 32 teeth						
Backe et al. 1989	2731	7104	1833	6048	0.0%	1.27 [1.21, 1.33]
Sensorn et al. 2012	2414	12128	694	7232	0.0%	2.07 [1.92, 2.25]
Subtotal (95% CI)		0		0		Not estimable
Total events	0		0			
Heterogeneity: Not applicable						
Test for overall effect: Not applicable						
Total (95% CI)		3108		2940	100.0%	2.27 [2.00, 2.58]
Total events	706		295			
Heterogeneity: Chi² = 74.71, df = 1 (P < 0.00001); I² = 99%						
Test for overall effect: Z = 12.58 (P < 0.00001)						
Test for subgroup differences: Not applicable						

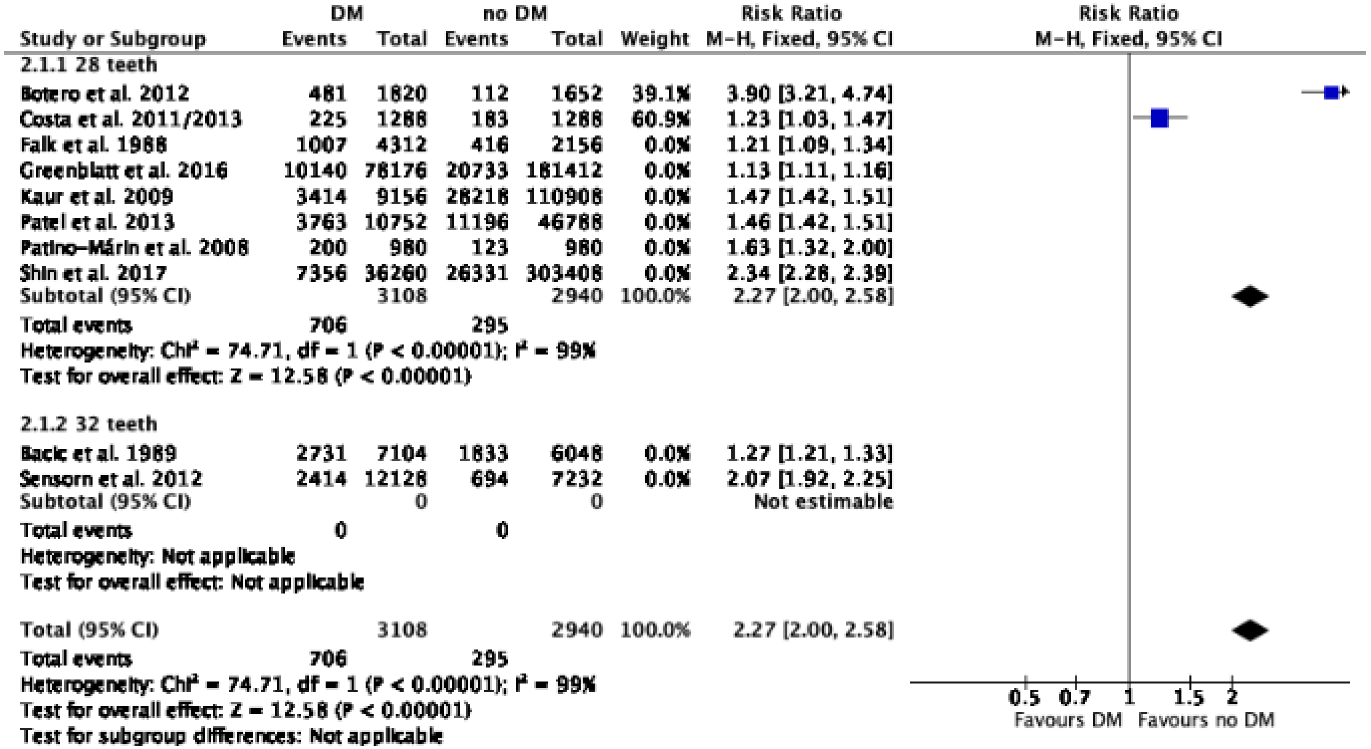

Online Appendix S3-4

Meta-analysis, subgroup analysis: forest plots using a random model the performed meta-analysis for the DM patients compared to non-DM on DM type II.

DM type II

Study or Subgroup	DM Events	DM Total	no DM Events	no DM Total	Weight	Risk Ratio M-H, Random, 95% CI
**3.1.1 28 teeth**						
Costa et al. 2011/2013	225	1288	183	1288	24.5%	1.23 [1.03, 1.47]
Falk et al. 1988	1007	4312	416	2156	0.0%	1.21 [1.09, 1.34]
Greenblatt et al. 2016	10140	78176	20733	181412	0.0%	1.13 [1.11, 1.16]
Kaur et al. 2009	2530	5096	14454	36792	25.6%	1.26 [1.23, 1.30]
Patel et al. 2013	3763	10752	11196	46788	0.0%	1.46 [1.42, 1.51]
Patino-Márin et al. 2008	200	980	123	980	24.2%	1.63 [1.32, 2.00]
Shin et al. 2017	7356	36260	26331	303408	25.6%	2.34 [2.28, 2.39]
Subtotal (95% CI)		43624		342468	100.0%	1.56 [1.02, 2.39]
Total events	10311		41091			
Heterogeneity: $Tau^2 = 0.18$; $Chi^2 = 1017.53$, df = 3 ($P < 0.00001$); $I^2 = 100\%$						
Test for overall effect: Z = 2.05 (P = 0.04)						
**3.1.2 32 teeth**						
Bacic et al. 1989	2731	7104	1833	6048	0.0%	1.27 [1.21, 1.33]
Sensorn et al. 2012	2414	12128	694	7232	0.0%	2.07 [1.92, 2.25]
Subtotal (95% CI)		0		0		Not estimable
Total events	0		0			
Heterogeneity: Not applicable						
Test for overall effect: Not applicable						
**Total (95% CI)**		43624		342468	100.0%	1.56 [1.02, 2.39]
Total events	10311		41091			
Heterogeneity: $Tau^2 = 0.18$; $Chi^2 = 1017.53$, df = 3 ($P < 0.00001$); $I^2 = 100\%$						
Test for overall effect: Z = 2.05 (P = 0.04)						
Test for subgroup differences: Not applicable						

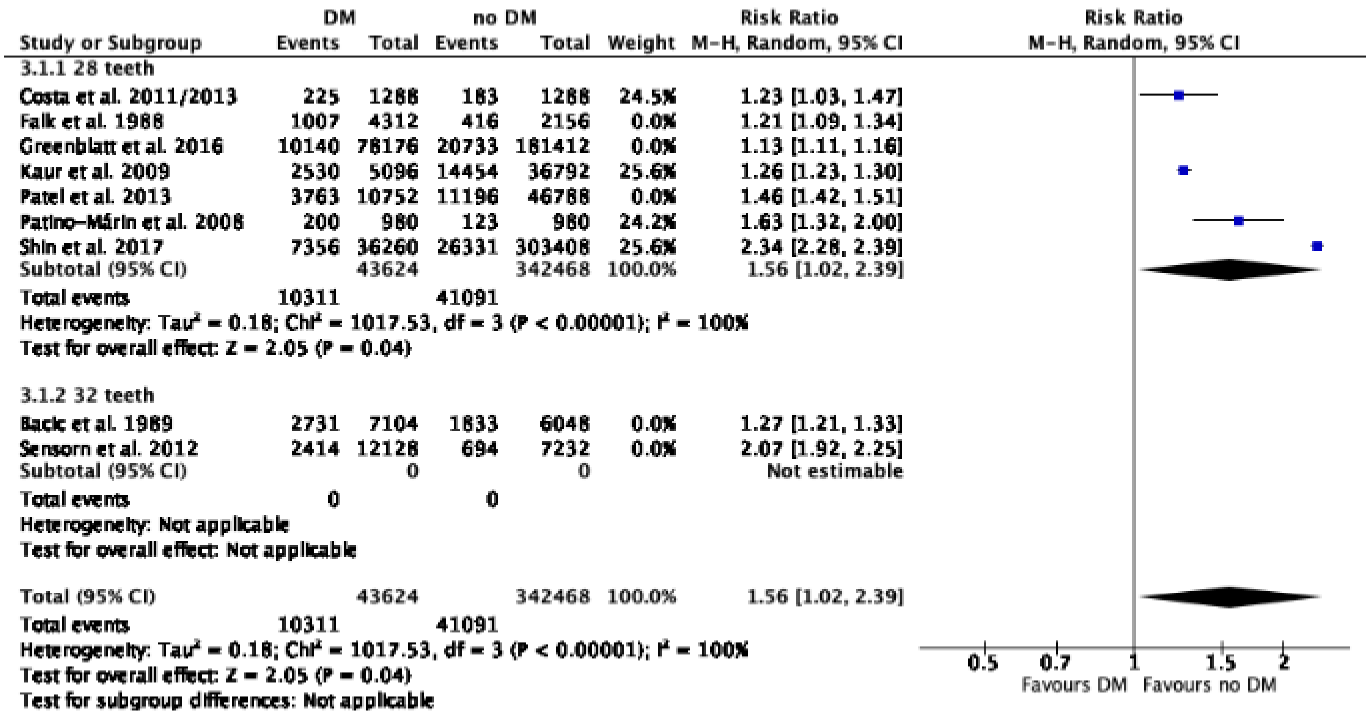

Online Appendix S3-5

Meta-analysis, subgroup analysis: forest plots using a fixed model the performed meta-analysis for the DM patients compared to non-DM on DM status (well/poor controlled, non-DM)

Well versus non-DM

Study or Subgroup	Good controlled DM Events	Good controlled DM Total	No DM Events	No DM Total	Weight	Risk Ratio M-H, Fixed, 95% CI
Costa et al. 2011/2013	96	644	183	1288	1.6%	1.05 [0.84, 1.32]
Greenblatt et al. 2016	4844	41104	20733	181412	98.4%	1.03 [1.00, 1.06]
Total (95% CI)		41748		182700	100.0%	1.03 [1.00, 1.06]
Total events	4940		20916			

Heterogeneity: $Chi^2 = 0.02$, df = 1 (P = 0.88); $I^2 = 0\%$
Test for overall effect: Z = 2.08 (P = 0.04)

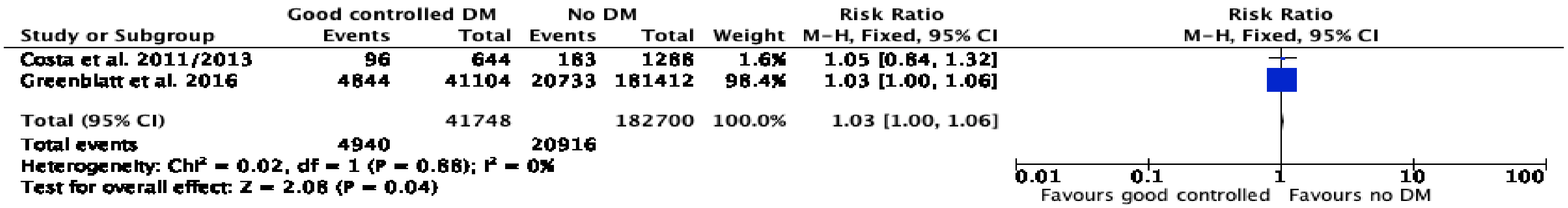

Poor versus non-DM

Study or Subgroup	Poor controlled DM Events	Poor controlled DM Total	No DM Events	No DM Total	Weight	Risk Ratio M-H, Fixed, 95% CI
Costa et al. 2011/2013	129	644	183	1288	1.7%	1.41 [1.15, 1.73]
Greenblatt et al. 2016	5296	37072	20733	181412	98.3%	1.25 [1.22, 1.29]
Total (95% CI)		37716		182700	100.0%	1.25 [1.22, 1.29]
Total events	5425		20916			

Heterogeneity: $Chi^2 = 1.31$, df = 1 (P = 0.25); $I^2 = 23\%$
Test for overall effect: Z = 15.90 (P < 0.00001)

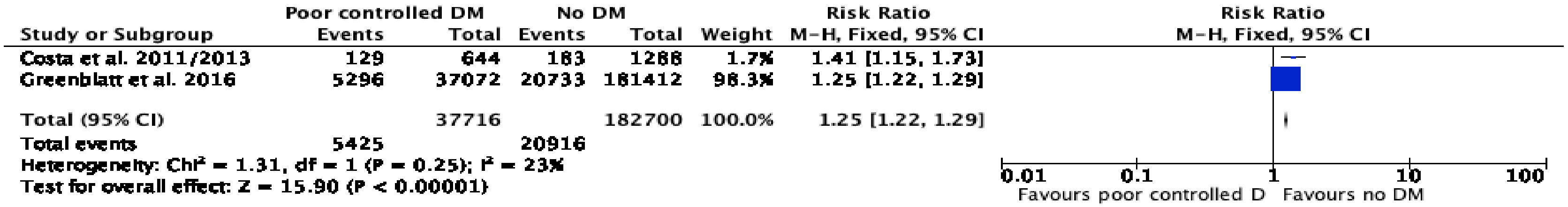

Poor versus well controlled DM

Study or Subgroup	Poor controlled DM Events	Poor controlled DM Total	Good controlled DM Events	Good controlled DM Total	Weight	Risk Ratio M-H, Fixed, 95% CI
Costa et al. 2011/2013	129	644	96	644	2.0%	1.34 [1.06, 1.71]
Greenblatt et al. 2016	5296	37072	4844	41104	98.0%	1.21 [1.17, 1.26]
Total (95% CI)		37716		41748	100.0%	1.21 [1.17, 1.26]
Total events	5425		4940			

Heterogeneity: $Chi^2 = 0.69$, df = 1 (P = 0.41); $I^2 = 0\%$
Test for overall effect: Z = 10.62 (P < 0.00001)

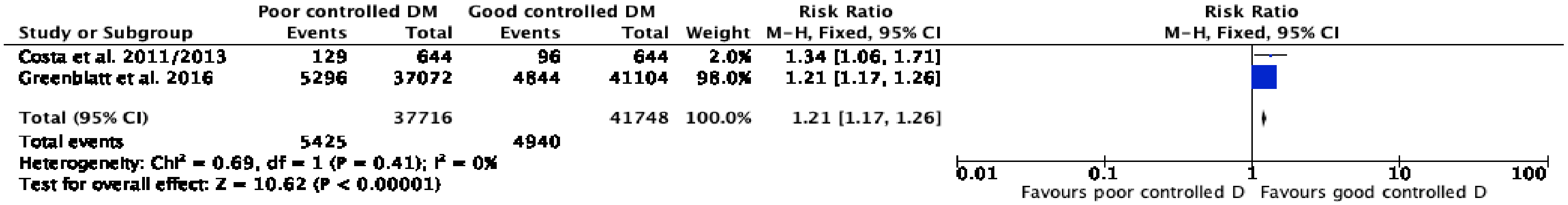

Online Appendix S4

Funnel plot: publication bias

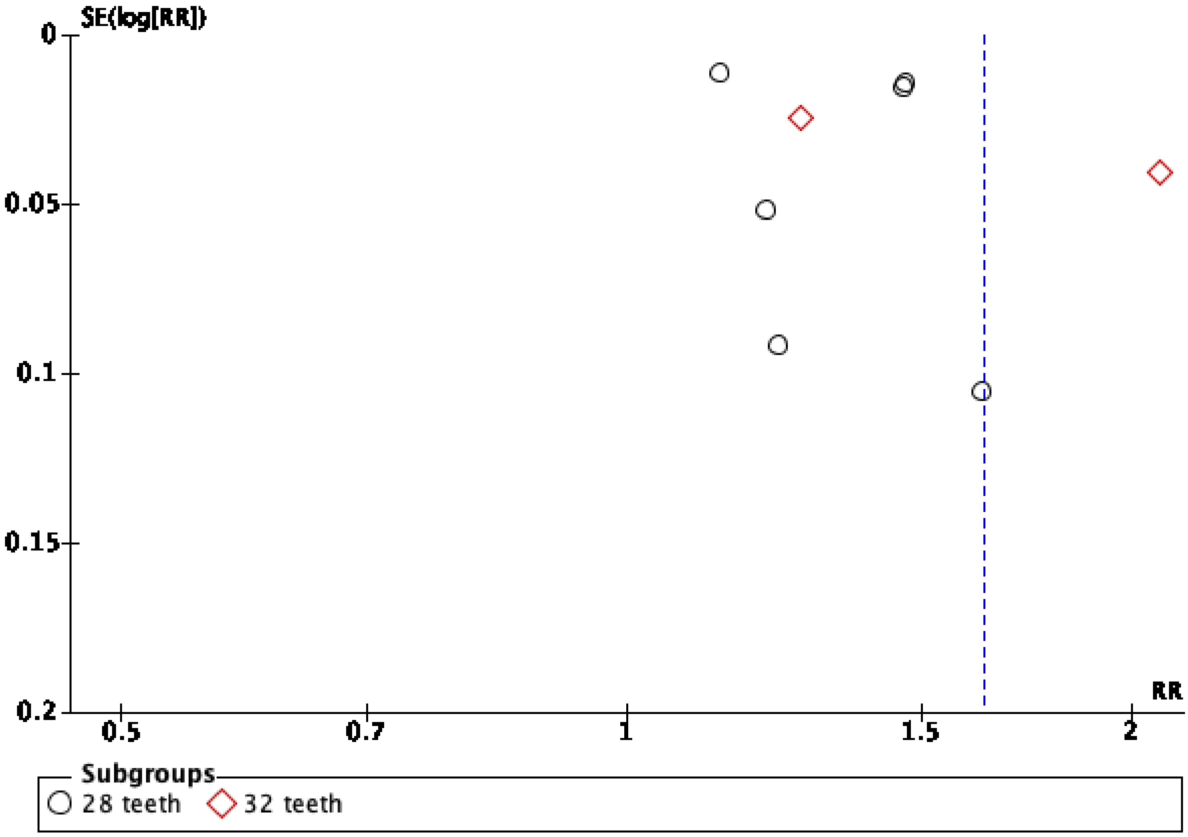

Online Appendix S5

List of abbreviations

Abbreviation	Meaning
ACTA	Academic Center for Dentistry Amsterdam
DES	Dagmar Else Slot; co-author of this paper
DM	Diabetes (mellitus), diabetic (mellitus)
DMFT	Decayed Missed Filled Teeth
EB	Eric Bakker, co-author of this paper
FEM	Fixed Effect Model
FPG	Fasting plasma glucose
GAW	Godefridus August van der Weijden; co-author of this paper
GRADE	Grading of Recommendations Assessment, Development and Evaluation
LPMW	Lotte Phinè Marie Weijdijk; first author of this paper
LZ	Laura Ziukaite, co-author of this paper
MA	Meta-analysis
MOOSE	Meta-Analysis of Observational Studies in Epidemiology
non-DM	No diabetes (mellitus), no diabetic (mellitus), people without diabetes (mellitus)
PRISMA	Preferred Reporting Items for Systematic Reviews and Meta-Analysis
REM	Random Effect Model
ROBINS-E	Risk of bias in observational studies of exposures
ROBINS-I	Risk of bias in non-randomized studies of interventions
RR	Relative risk
SR	Systematic review
QHRQoL	Oral health-related quality of life
QoL	Quality of life

Online Appendix S6

MOOSE (Meta-analyses and Systematic Review of Observational Studies in Epidemiology) Checklist

Reporting Criteria	Reported (Yes/No)	Reported on Page
**Reporting of Background**		
Problem definition	Yes	5-7
Hypothesis statement	Yes	7
Description of Study Outcome(s)	Yes	6,7, 9, 10
Type of exposure or intervention used	Yes	6,7, 9, 10
Type of study design used	Yes	6,7
Study population	Yes	7-9
**Reporting of Search Strategy**		
Qualifications of searchers (e.g., librarians and investigators)	Yes	3, 7
Search strategy, including time period included in the synthesis and keywords	Yes	7,8
Effort to include all available studies, including contact with authors	Yes	7, 8, 49, 50
Databases and registries searched	Yes	7, 8
Search software used, name and version, including special features used (e.g., explosion)	Yes	7, 8, 35
Use of hand searching (e.g., reference lists of obtained articles)	Yes	7, 8, 32
List of citations located and those excluded, including justification	Yes	13, 32, 49, 50
Method for addressing articles published in languages other than English	Yes	8, 49, 50
Method of handling abstracts and unpublished studies	Yes	7, 32
Description of any contact with authors	Yes	13, 49, 50
**Reporting of Methods**		
Description of relevance or appropriateness of studies assembled for assessing the hypothesis to be tested	Yes	8, 9
Rationale for the selection and coding of data (eg, sound clinical principles or convenience)	Yes	8, 9
Documentation of how data were classified and coded (e.g., multiple raters, blinding, and interrater reliability)	Yes	8-11

Assessment of confounding (eg, comparability of cases and controls in studies where appropriate	Yes	9, 12, 43, 48, 51-212
**Reporting Criteria**		
Assessment of study quality, including blinding of quality assessors; stratification or regression on possible predictors of study results	Yes	9, 12, 43, 48, 51-212
Assessment of heterogeneity	Yes	10-12
Description of statistical methods (e.g., complete description of fixed or random effects models, justification of whether the chosen models account for predictors of study results, dose-response models, or cumulative meta-analysis) in sufficient detail to be replicated	Yes	10-12, 45-47
Provision of appropriate tables and graphics	Yes	33, 34, 45-47, 213-222
**Reporting of Results**		
Table giving descriptive information for each study included	Yes	36-41
Results of sensitivity testing (eg, subgroup analysis)	Yes	33, 34, 45-47, 213-222
Indication of statistical uncertainty of findings	Yes	11, 15, 16, 33, 34, 45-47, 213, 222
**Reporting of Discussion**		
Quantitative assessment of bias (e.g., publication bias)	Yes	16, 19, 223
Justification for exclusion (e.g., exclusion of non–English-language citations)	Yes	13, 18, 22, 32, 49, 50
Assessment of quality of included studies	Yes	43, 51-212
**Reporting of Conclusions**		
Consideration of alternative explanations for observed results	Yes	18-22
Generalization of the conclusions (i.e., appropriate for the data presented and within the domain of the literature review)	Yes	16, 17, 22, 23, 48
Guidelines for future research	Yes	22
Disclosure of funding source	Yes	2

Online Appendix S7
PRISMA checklist

PRISMA 2009 Checklist

Section/topic	#	Checklist item	Reported on page #
**TITLE**			
Title	1	Identify the report as a systematic review, meta-analysis, or both.	1, 2
**ABSTRACT**			
Structured summary	2	Provide a structured summary including, as applicable: background; objectives; data sources; study eligibility criteria, participants, and interventions; study appraisal and synthesis methods; results; limitations; conclusions and implications of key findings; systematic review registration number.	4
**INTRODUCTION**			
Rationale	3	Describe the rationale for the review in the context of what is already known.	6
Objectives	4	Provide an explicit statement of questions being addressed with reference to participants, interventions, comparisons, outcomes, and study design (PICOS).	6, 7
**METHODS**			
Protocol and registration	5	Indicate if a review protocol exists, if and where it can be accessed (e.g., Web address), and, if available, provide registration information including registration number.	7
Eligibility criteria	6	Specify study characteristics (e.g., PICOS, length of follow-up) and report characteristics (e.g., years considered, language, publication status) used as criteria for eligibility, giving rationale.	7-11
Information sources	7	Describe all information sources (e.g., databases with dates of coverage, contact with study authors to identify additional studies) in the search and date last searched.	7, 8, 32, 49, 50

Search	8	Present full electronic search strategy for at least one database, including any limits used, such that it could be repeated.	7, 8, 32, 35
Study selection	9	State the process for selecting studies (i.e., screening, eligibility, included in systematic review, and, if applicable, included in the meta-analysis).	8,9
Data collection process	10	Describe method of data extraction from reports (e.g., piloted forms, independently, in duplicate) and any processes for obtaining and confirming data from investigators.	10, 11
Data items	11	List and define all variables for which data were sought (e.g., PICOS, funding sources) and any assumptions and simplifications made.	7-10
Risk of bias in individual studies	12	Describe methods used for assessing risk of bias of individual studies (including specification of whether this was done at the study or outcome level), and how this information is to be used in any data synthesis.	9, 10
Summary measures	13	State the principal summary measures (e.g., risk ratio, difference in means).	11, 12
Synthesis of results	14	Describe the methods of handling data and combining results of studies, if done, including measures of consistency (e.g., I^2) for each meta-analysis.	11, 12

Section/topic	#	Checklist item	Reported on page #
Risk of bias across studies	15	Specify any assessment of risk of bias that may affect the cumulative evidence (e.g., publication bias, selective reporting within studies).	11, 12, 17, 18, 43, 51-212, 223
Additional analyses	16	Describe methods of additional analyses (e.g., sensitivity or subgroup analyses, meta-regression), if done, indicating which were pre-specified.	11, 12, 15, 16
**RESULTS**			
Study selection	17	Give numbers of studies screened, assessed for eligibility, and included in the review, with reasons for exclusions at each stage, ideally with a flow diagram.	13, 32
Study characteristics	18	For each study, present characteristics for which data were extracted (e.g., study size, PICOS, follow-up period) and provide the citations.	13, 14, 36, 41
Risk of bias within studies	19	Present data on risk of bias of each study and, if available, any outcome level assessment (see item 12).	14, 43, 51-212,

Results of individual studies	20	For all outcomes considered (benefits or harms), present, for each study: (a) simple summary data for each intervention group (b) effect estimates and confidence intervals, ideally with a forest plot.	13-16, 33, 34, 36-41, 44-47, 213-222
Synthesis of results	21	Present results of each meta-analysis done, including confidence intervals and measures of consistency.	15, 16, 33, 34, 45, 47, 213, 222
Risk of bias across studies	22	Present results of any assessment of risk of bias across studies (see Item 15).	12, 14, 16, 17, 43, 48, 51-212
Additional analysis	23	Give results of additional analyses, if done (e.g., sensitivity or subgroup analyses, meta-regression [see Item 16]).	15, 16, 33, 34, 44-47, 213, 223
**DISCUSSION**			
Summary of evidence	24	Summarize the main findings including the strength of evidence for each main outcome; consider their relevance to key groups (e.g., healthcare providers, users, and policy makers).	18-21
Limitations	25	Discuss limitations at study and outcome level (e.g., risk of bias), and at review-level (e.g., incomplete retrieval of identified research, reporting bias).	16-23, 48
Conclusions	26	Provide a general interpretation of the results in the context of other evidence, and implications for future research.	18-23
**FUNDING**			
Funding	27	Describe sources of funding for the systematic review and other support (e.g., supply of data); role of funders for the systematic review.	2
